# Recent advances on the transition-metal-catalyzed synthesis of imidazopyridines: an updated coverage

**DOI:** 10.3762/bjoc.15.165

**Published:** 2019-07-19

**Authors:** Gagandeep Kour Reen, Ashok Kumar, Pratibha Sharma

**Affiliations:** 1 School of Chemical Sciences, Devi Ahilya University, Indore, (M. P.), India

**Keywords:** C–H activation/functionalization, coupling reactions, imidazopyridines, multicomponent reactions, transition metal catalysis

## Abstract

A comprehensive account of recent advances in the synthesis of imidazopyridines, assisted through transition-metal-catalyzed multicomponent reactions, C–H activation/functionalization and coupling reactions are highlighted in this review article. The basic illustration of this review comprises of schemes with concise account of explanatory text. The schemes depict the reaction conditions along with a quick look into the mechanism involved to render a deep understanding of the catalytic role. At some instances optimizations of certain features have been illustrated through tables, i.e., selectivity of catalyst, loading of the catalyst and percentage yield with different substrates. Each of the reported examples has been rigorously analyzed for reacting substrates, reaction conditions and transition metals used as the catalyst. This review will be helpful to the chemists in understanding the challenges associated with the reported methods as well as the future possibilities, both in the choice of substrates and catalysts. This review would be quite appealing to a wider range of organic chemists in academia and industrial R&D sectors working in the field of heterocyclic syntheses. In a nutshell, this review will be a guiding torch to envisage: (i) the role of various transition metals in the domain dedicated towards method development and (ii) for the modifications needed thereof in the R&D sector.

## Introduction

**Figure 1:**
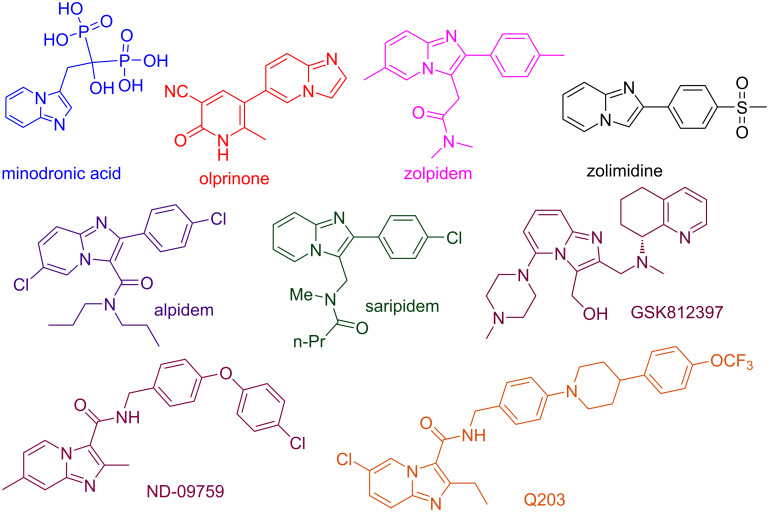
Various drugs having IP nucleus.

The structural diversity and biological importance of nitrogen-containing heterocycles have blossomed in the last many years. These heterocyclic scaffolds occupy a pivotal role in the realm of both natural products and synthetic organic chemistry. Their importance as precursors to many biologically active compounds has created a tremendous amount of focused attention on developing methods to functionalize these systems [1–2]. In one such context, imidazopyridines are one of the fascinating classes of fused N-heterocyclic scaffolds of versatile concern. Their chemistry has drawn substantial attention in last few years owing to their involvement in various medicinal applications viz., anti-inflammatory, anticancer, antibacterial, anxioselective and antiprotozoal [3–5]. Furthermore, a number of drugs embracing the imidazopyridine (IP) skeleton have been commercially marketed with their trade names as minodronic acid (for the treatment of osteoporosis), olprinone (for acute heart failure), zolpidem (for treatment of insomnia), zolimidine (antiulcer drug), alpidem and saripidem (anxiolytic agents) and some are under development, like GSK812397 (for the treatment of HIV), ND-09759 and Q203 (for tuberculosis) (Figure 1) [6–10].

The synthesis of imidazopyridines (IPs) is widely described in the literature encompassing a number of reactions like oxidative cyclizations [11], oxidative coupling reactions [12], Vilsmeier type cyclizations [13], intramolecular aminooxygenation/C–H amination reactions [14–15], Groebke–Blackburn–Bienayme (GBB) reactions [16–18] and many more. It was observed that most of the methods utilize 2-aminopyridines as one of the starting materials due to its binucleophilic nature; associated with exocyclic amino group and endocyclic pyridinium nitrogen [19]. Recently, A^3^ coupling of alkynes, aldehydes, and aminopyridines have been developed for efficient syntheses of IPs [20]. In recent years much attention has been paid towards the exploration of transition metals (TMs) as homogeneous as well as heterogeneous catalytic system like Pd(OAc)_2_ [21], CuI [22], ZnO [23], ZnI_2_ [24], Cu(OTf)_2_ [25], Sc(OTf)_3_ [26], RuCl_3_ [27], [Cp^*^RhCl_2_]_2_ (Cp^*^: pentamethylcyclopentadiene) [28], FeCl_3_ [29], NiFe_2_O_4_ [30], CuO [31], mixed-metal oxides [32] etc. Some of the reactions have utilized metal salts like Cu(OAc)_2_, Ag_2_CO_3_, AgOAc, etc. (reference papers of this review) as an oxidant to carry out the synthesis among which preference was given to the use of oxygen and air as greener oxidants [33].

During the writing of this review, we came across some reviews on IPs, however, their coverage is limited to the imidazo[1,2-*a*]pyridine nucleus deriving from either a particular starting material or to one type of reaction procedure (particularly C–H activation) with literature up to 2015 [34–39]. This review covers a substantial time period of the literature, from the year 2006 to August 2018 (except for the reports mentioned under previously reported reviews), with state-of-the-art methodologies, and reflecting some of the changing approaches and challenges encountered in the field of catalytic synthetic chemistry.

**Figure 2:**
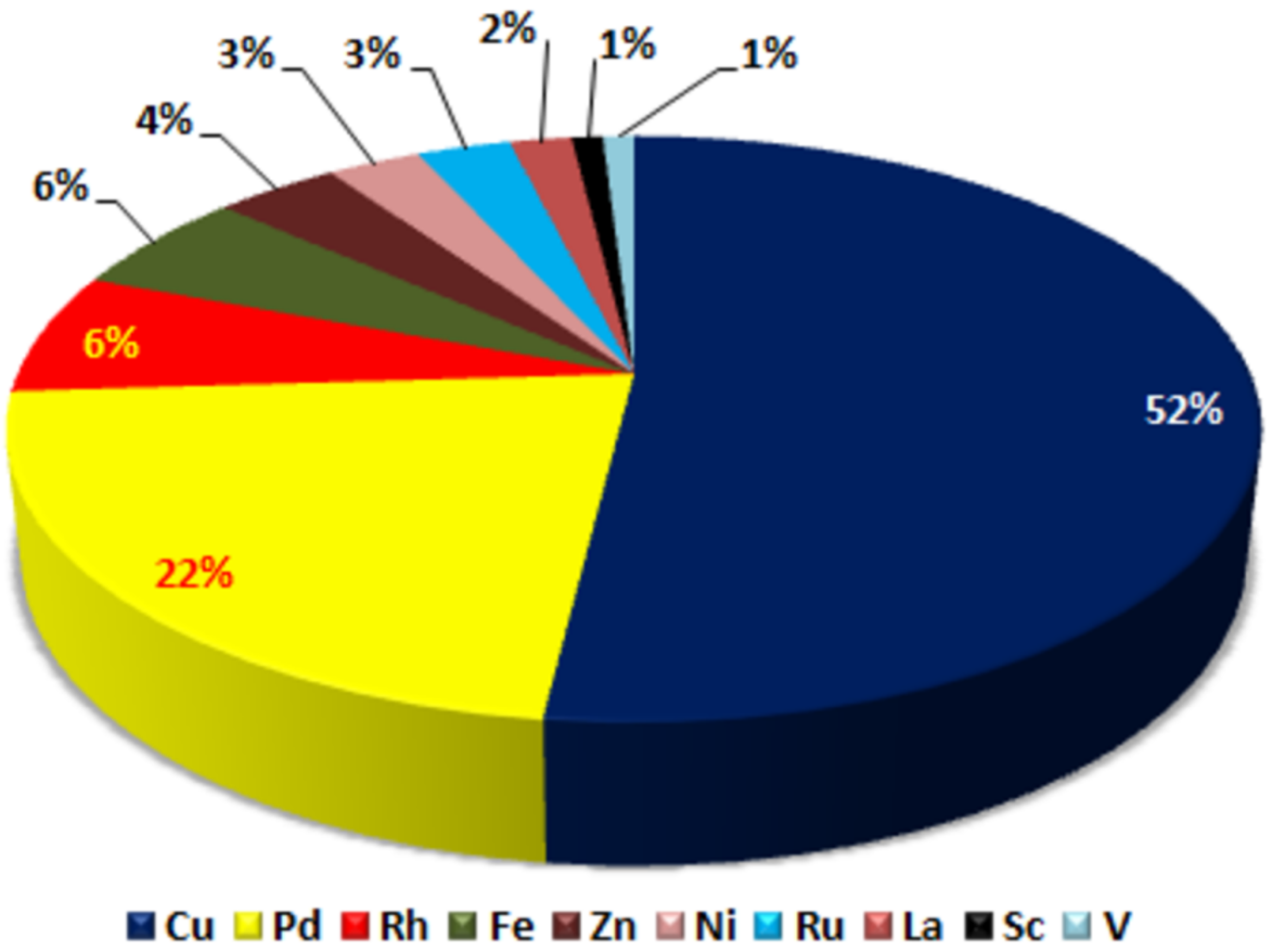
Participation percentage of various TMs for the syntheses of IPs.

The prime focus of this review is on the TM-catalyzed synthesis of differently fused IPs. In this review, a concise account on imidazopyridine (IP) syntheses has been documented for the benefit of the chemists working in the field. This will assist them in unraveling the possibilities for efficient synthesis of different IP derivatives supported by TMs as the catalysts. It will be of great help for them to consider this as ready reckoner of rich literature for TM-catalyzed IP syntheses and to help the chemists to understand some of the challenges associated with the reported methodologies. Amongst various reported TMs, the participation of copper as the catalyst has been used in the largest number of cases followed by the use of palladium, rhodium, and others. It has been further observed that lanthanum, scandium, and vanadium have been very seldomly used. An overview of the participation of various TMs is depicted in Figure 2.

### The role of copper in synthetic chemistry

In comparison with other TMs copper-metal-based catalysts are cheap, nontoxic, abundant and environmentally benign [40]. Chemistry of Cu is extremely rich as it can exist in variable oxidation states (Cu(0), Cu(I), Cu(II), and Cu(III)). Copper has been known for a long time to act as a catalyst for cross-coupling reactions (Ullmann–Goldberg reaction), cyanation of aryl halides (Rosenmund–von Braun reaction), Hurtley reaction and intermolecular oxidative cyclization of haloalkynes [41–44]. Various copper salts have been used as Lewis acid in homogeneous catalysis. CuO nanoparticles (NPs) were used for C–N, C–S, C–O cross-coupling reactions and C-arylation. Recently, exploiting the cross-coupling tendency of CuO NPs, Reddy et al. have reported their use as a heterogeneous, recyclable catalyst in the N-arylation of indoles [45–46]. Copper catalysts have shown exceptional enantioselectivity for reactions such as hydrosilylation, hydroboration, and heterogeneous as well as homogeneous hydrogenation [47–49]. Also, the copper salts found used as oxidants in a number of organic reactions. In the syntheses of IPs, various forms of copper viz., salts, complexes, MOFs, oxides, and nanoparticles (NPs) have been used as the catalytic system in both multicomponent reactions (MCRs) as well as derivatization methodologies.

### The role of palladium in synthetic chemistry

Pd-catalyzed cross-coupling reactions have laid down the foundation of new C–C bond formations [50–51]. A number of Pd-catalyzed organic reactions viz., C–N coupling, amination and intramolecular amidation, cyclization, and Suzuki–Miyaura coupling [52–55] have recently been reported in the literature. The Pd-catalyzed Sonogashira reaction has successfully constructed arylated internal alkynes that are important intermediates in organic synthesis, molecular electronics and polymers [56]. C–N bond forming reactions between aryl halides and amines/amides/sulfonamides have been extensively studied in the past few years [57]. Recently, Buchwald and Castillo have reviewed the exceptional utility of Pd-catalyzed C–N cross-coupling reactions for the preparation of anilines and aniline derivatives [58]. In many of the reactions, palladium was used along with a co-catalyst to enhance its catalytic activity. It is revealed from the literature that next to the copper, palladium catalysts in the form of salts (with or without ligands), complexes, NPs, etc. find their usage on mass scale syntheses of IPs.

### The role of zinc in synthetic chemistry

Zinc salts being inexpensive, environmentally benign, low in toxicity and fits to the tenets of green chemistry. Zinc in the form of its salts, complexes (chelated by mono/di/tri/tetradentate ligands), oxides and sulfides proved to be a promising and active catalyst for organic chemists in both homogeneous as well as heterogeneous reaction systems [23,59–60]. They have been employed for a number of organic reactions viz., hydrosilylation of aldehydes and ketones, acetylation reactions, redox reactions, oxidative esterification, etc. [61–63]. Thus, in concern with increasing demand for sustainable development, a growing number of catalytic systems based on zinc with excellent activity has been designed and used.

### The role of iron in synthetic chemistry

Iron being the most abundant heavy element on earth with low biological toxicity along with cost economy and high reactivity was explored by Kharasch and Fields in the 1940s and Tamura and Kochi in 1970s in the field of catalysis [64–65]. Iron in the form of its salts, oxides, iron–NHC complexes, iron pincer complexes, ferrocenes and half sandwiched iron complexes found to be the center of attraction in the field of catalytic organic synthesis. Variable oxidation states of iron (–2 to +5) allow it to catalyze a number of organic reactions viz., nucleophilic substitutions, addition reactions, hydrogenations/hydrosilylations, cycloisomerizations, electrophilic aromatic substitutions, cross-coupling reactions, oxidative additions and reductive eliminations [66]. Iron–catalyzed C–H functionalization helped in the introduction of various organic groups to numerous substrates [67–68]. Recently attention has been diverted towards the use of magnetic NPs, in this context iron oxide played a pivotal role as a catalyst or as support for some homogeneous or other metal catalysts for the sake of easy recovery and reusability [69].

### The role of rhodium in synthetic chemistry

Rhodium in the form of Wilkinsons catalyst [RhCl(PPh_3_)_3_] played a major role in the field of catalysis [70]. Rhodium is capable of catalyzing a number of organic reactions viz., heterocyclic alkylation and arylation with a high level of functional group compatibility, intramolecular alkylations, inter and intramolecular C–H bond activation and regio/stereoselective homocoupling of alkynes [71–73]. Group of Cho and Chang have unprecedentedly reported a Rh catalytic system for facile addition of heteroarenes to both alkenes and alkynes [74]. Rh(I)/Rh(II)/Rh(III) catalysts have been used in asymmetric syntheses of chiral heterocycles that have been effectively reviewed by Chen and Xu in 2017 [75].

### The role of ruthenium in synthetic chemistry

Over the past decade, Ru and its complexes were used as a catalyst in various organic processes and have attracted considerable interest in this regard. Ru has efficiently catalyzed C–H activation reactions for C–C bond formation, aza-Michael reactions and many more MCRs [27,76–77]. During the writing of this review, we came through the fact that ruthenium catalysts were mostly used for functionalization/derivatization reactions compared to MCRs in the syntheses of IPs.

### The role of lanthanum/scandium/nickel/vanadium in synthetic chemistry

The application of scandium complexes in organic chemistry has been very scarce due to their low availability and difficulties in separation. This problem was resolved by the introduction of scandium triflate (Sc(OTf)_3_) as a promising reusable Lewis acid in Diels–Alder reactions by Kobayashi [78]. However, in recent years scandium(III) trifluoromethanesulfonate (Sc(OTf)_3_) has emerged as an efficient, mild, commercially available, inexpensive, water-tolerant Lewis acid catalyst in the formation of both carbon–carbon and carbon–heteroatom bonds, and thereby the formation of various biologically promising organic compounds [68]. Important advances in scandium-catalyzed chemistry include [4 + 2] and [2 + 2] cycloaddition reactions, Baeyer–Villiger reactions, epoxidations of alkenes, intramolecular ring expansions, hydroaminations, and amination reactions and carbonyl–ene reactions for the formation of C–C bond. Scandium-catalyzed reactions represented remarkable enantioselectivities [79–81]. Recently the group of Rani has developed fly ash-supported Sc(OTf)_3_ for Friedal–Crafts acylation reaction, also the group of Fukuzawa has exploited Sc(OTf)_3_ in the synthesis of *N*-substituted 1,4-DHPs [26,82].

Enhancement in reactivity and selectivity of many reactions, ability to polarize bonds upon coordination and thus altering their electrophilicity made lanthanides a catalyst of choice. Lanthanum complexes are widely used in synthetic chemistry for cycloadditions, reductions, benzimidazole syntheses, Biginelli reactions, hydrophosphinations of unsaturated substrates, double hydrophosphinylations of unactivated nitriles, Grignard additions, C–C bond formations, and ring opening reactions [83–87]. Along with these nickel and vanadium were also well known to catalyze a number of organic reactions. Recent reviews published on the catalytic applications of these metals in various forms have underlined the indispensable contribution in the field of TM catalysis [88]. Vanadium in the form of vanadium oxide and complexes has been reported to participate in numerous organic reactions including aerobic oxidation [89–90]. A report of Carsten Bolm has delineated the use of vanadium complexes in enantioselective oxidation of alcohols and asymmetric sulfide oxidation [91]. Diverse reactivity, cost efficiency and variable oxidation state [Ni(0)–Ni(IV)] associated with nickel led to remarkable developments in the field of catalytic applications [68]. Nickel catalysis involved cycloaddition, cyclization, C–H bond functionalization, and multicomponent coupling reactions [92–93].

## Review

### Multicomponent reactions for the synthesis of imidazopyridines

One of the major challenges in organic synthesis is the creation of diverse and complex molecules from simple and readily available substrates. MCRs constitute one of the most efficient tools in modern synthetic chemistry, with high atom efficiency, quick and simple implementation, ecofriendliness and a diverse target-oriented synthesis [94]. In addition, both waste production and multistep problems resulting in the expenditure of human labor are significantly reduced. MCRs have been given much attention in various research fields, such as the discovery of lead compounds in medicinal chemistry, or combinatorial chemistry [95–96].

#### Copper-catalyzed synthetic protocols

**Scheme 1:**
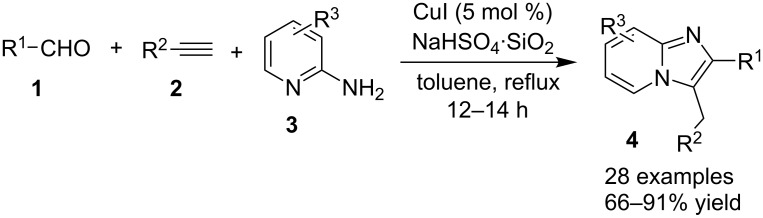
CuI–NaHSO_4_·SiO_2_-catalyzed synthesis of imidazo[1,2-*a*]pyridines.

Ghosh and Mishra [97] have successfully reported the synthesis of imidazo[1,2-*a*]pyridines in a three-component domino reaction of aldehydes **1**, 2-aminopyridines (2-APs) **3** and terminal alkynes **2**, catalyzed by CuI and co-catalyzed by the NaHSO_4_·SiO_2_ system (Scheme 1).

**Scheme 2:**
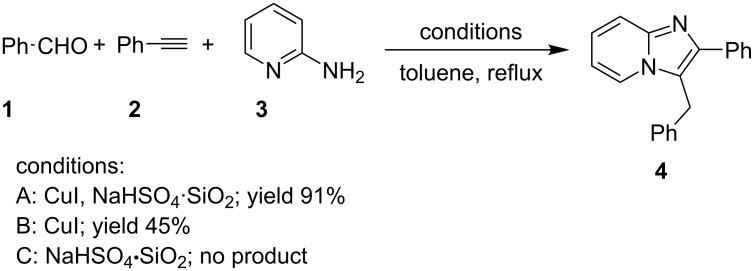
Experimental examination of reaction conditions.

The reaction performed with CuI alone gave a moderate yield of the product (only 45%) whereas NaHSO_4_·SiO_2_ alone was unable to complete the reaction. Therefore, the synergistic effect of the CuI–NaHSO_4_·SiO_2_ system for successful completion of the reaction was proven under optimized conditions. (Scheme 2, A, B and C). In order to ascertain the mechanistic pathways, the reaction was performed in two different ways; in a first way, 2-APs **3** were allowed to react with aldehydes **1** in the presence of NaHSO_4_·SiO_2_ and the product obtained was refluxed with phenylacetylene (as terminal alkyne, **2**) in the presence of CuI to obtain the product, however, in the second pathway, **1** and **2** were taken together in the presence of CuI and the product obtained was treated with **3** in the presence of NaHSO_4_·SiO_2_ to produce the desired IP. Both the routes resulted in the same final product without any substantial change in the yield. The generality of the reaction was studied with differently substituted **1**, **3** and **2** having both electron-withdrawing (EW) and electron-donating groups (EDGs) at different positions on the phenyl ring. However, the use of aliphatic aldehydes such as 3-methylbutanal and cyclohexanecarbaldehyde and aliphatic acetylene viz., hex-1-yne resulted in a comparatively lower yield of 66, 70 and 60%, respectively. Heteroaromatic aldehydes such as thiophene-2-carbaldehyde on the other hand resulted in appreciable yields under similar reaction conditions. The authors have successfully constructed a big library of 28 compounds with varying structure.

**Scheme 3:**
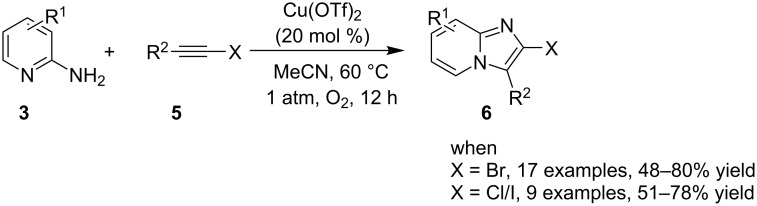
One-pot tandem reaction for the synthesis of 2-haloimidazopyridines.

In 2013, Gao et al. have exploited the TM-catalyzed nucleophilic addition reactions of haloalkynes **5** followed by carbon–nitrogen bond formation, as an efficient methodology for the synthesis of 2-haloimidazopyridines **6** [44] The reaction was catalyzed by copper in an atmosphere of oxygen for approximately 12 h of reaction time. The major challenge before their developed protocol was to retain the reactive halide substituent in the product which was successfully overcome by this protocol along with the synthesis of pyrazines and pyrimidines in high yield (Scheme 3).

**Scheme 4:**
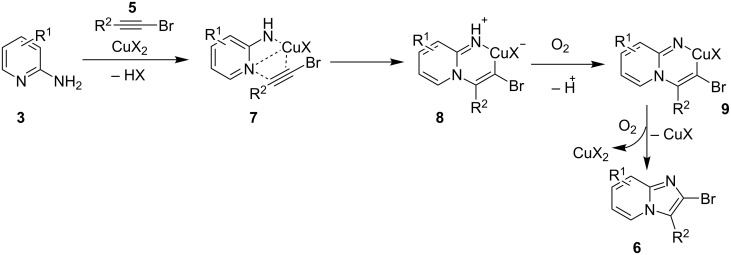
Mechanistic scheme for the synthesis of 2-haloimidazopyridine.

**Scheme 5:**
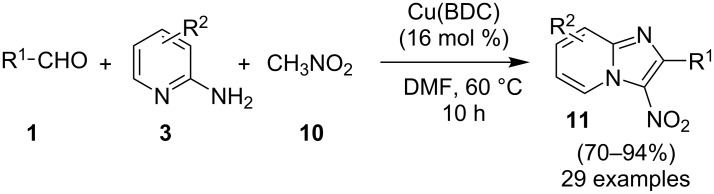
Copper-MOF-catalyzed three-component reaction (3-CR) for imidazo[1,2-*a*]pyridines.

**Scheme 6:**
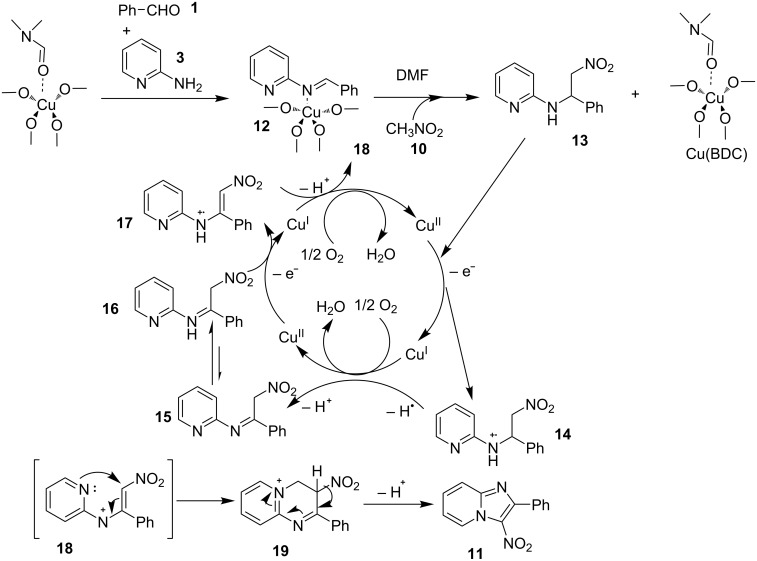
Mechanism for copper-MOF-driven synthesis.

Furthermore, easy functionalization of the products from the viewpoint of reactive halide made them valuable synthons. Molecular oxygen used served the role of oxidant for generating a Cu(III) intermediate during the reaction. The absence of oxygen atmosphere gave the yield in a slightly lesser amount with increased loading of catalyst to almost 1 equivalent. This reaction avoided the use of any additive; moreover, the presence of bases in this methodology resulted in homocoupling of bromoalkynes. Among the examined Cu(I) and Cu(II) salts, Cu(OTf)_2_ was proven to be the optimal one. Both EW and EDGs were well tolerated except 2-amino-6-methylpyridine which might be sterically hindered due to methyl group at 6-position. The use of aliphatic alkynes gave an optimal yield of the final product in the range of 50–78%. Successful formation of the product on application of 2,2,6,6-tetramethylpiperidine-1-oxyl (TEMPO: a radical scavenger) has nullified the probability of radical pathway. It was thought to be initiated by the coordination of 2-AP to Cu(OTf)_2_, forming an intermediate **7**, that was followed by migratory insertion by haloalkyne (Scheme 4). The organocopper species **8** thus formed would undergo deprotonation/oxidation and finally reductive elimination to give the cyclized product **6** (Scheme 4). Along with the unprecedented intermolecular oxidative diamination of haloalkynes, mild reaction conditions and efficient conversion of the alkyl-substituted haloalkynes to the halogenated product were great improvements over existing methods. Stimulated by the work of Luz et al. [98] and Phan et al. [99] with Cu(BDC)MOF (BDC: 1,4-benzenedicarboxylate), Puthiaraj and co-workers have unprecedently discovered the catalytic activity of this metal-organic framework (MOF) for the synthesis of imidazo[1,2-*a*]pyridines [100]. The three-component, one-pot reaction between **1**, **3** and nitromethane (**10**, Scheme 5) involved an intermolecular aza-Michael addition with a subsequent intramolecular cyclization catalyzed by Cu(BDC)MOF. Further, the LC–MS study of the reaction mixture has shown the imine formation to be the major pathway rather than β-nitrostyrene. The reaction involved a two-step reaction mechanism in which aza-Michael adduct **13** was formed in the first step which was converted to the final product via radical cation intermediate **17** in the second step (Scheme 6). This additive-free approach offered an easy separation and reusability of heterogeneous catalyst along with the use of air as environmentally benign oxidant.

**Scheme 7:**
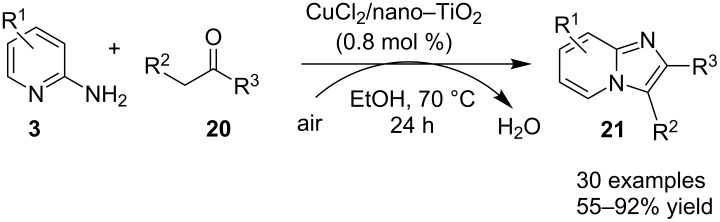
Heterogeneous synthesis via titania-supported CuCl_2_.

**Scheme 8:**
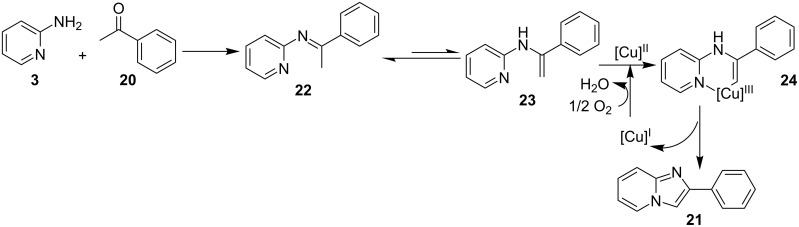
Mechanism involving oxidative C–H functionalization.

The use of zinc and iron MOFs have resulted in lesser yield than Cu-MOF. The reaction has well tolerated different substituent on the reactants except for 4-NO_2_; 3,4-dichlorobenzaldehyde, 2-aminopyrimidine, and 2,4,6-triamino-1,3,5-triazine which failed to give the desired product. Also, benzaldehydes substituted with EDGs at *p*- and *m*-positions gave a higher yield than sterically hindered *o*-substituents and EWGs. Moreover, the absence of metal leaching as tested by atomic absorption spectroscopy (AAS) demonstrated the heterogeneous nature of the used catalyst. Recently, a robust, ligand and additive-free heterogeneously catalyzed synthesis of imidazo[1,2-*a*]pyridines from **3** and ketones **20** has been reported (Scheme 7) [101]. Titania-supported CuCl_2_ (CuCl_2_/nano TiO_2_) played the role of catalyst in this transformation under aerobic conditions. Titania support helped in recyclability of the copper salt to four synthetic cycles without loss in activity. In addition to this, TiO_2_ is more stable, abundant, nontoxic, and economical [102]. Out of different supports examined like Al_2_O_3_, ZrO_2_, CeO_2_, SiO_2_, Al-Ti, OMS-2, C, etc., TiO_2_ was found to be the best for this transformation. Furthermore, a library of compounds was synthesized considering ketones having ED and EWGs, with sensitive –NO_2_ groups along with heteroaromatic ketones as well as α,β-unsaturated ketones. Differently substituted compound **3** (except –NO_2_ substitution) gave moderate to good yields (55–84%). Moreover, the protocol has demonstrated the successful synthesis of drug zolimidine on a gram scale. The mandatory role of oxygen or air as an oxidant for this reaction was proved by reaction failure under the nitrogen atmosphere. This synthesis ruled out the possibility of Ortoleva–King reaction under these conditions as the use of a catalytic amount of iodine in the absence of copper did not result in the desired product keeping the reaction conditions intact. However, based on control experiments and the works of Zhang and Ma’s group [103–104], oxidative C–H functionalization was expected to be more likely involved in the cyclization strategy (Scheme 8).

**Scheme 9:**
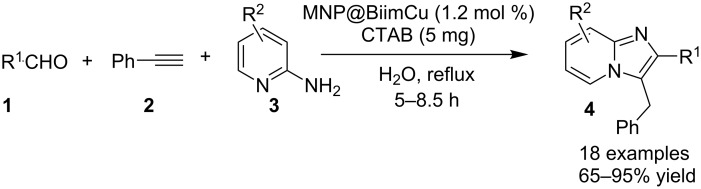
Heterogeneous synthesis of IPs.

Heterogenization of organic catalysts on Fe_3_O_4_ NPs has attracted much attention as these catalysts can be recovered by an external magnetic field and reused in subsequent reactions. Some metal complexes of 2,2-biimidazole (H_2_Biim) found to be effective catalysts in many organic transformations [105–106]. Inspired from the history of IP synthesis Tajbakhsh et al. have utilized the biimidazole Cu(I) complex supported on magnetic Fe_3_O_4_ (MNP@BiimCu) as a heterogenous catalyst for the synthesis of imidazo[1,2-*a*]pyridine in an aqueous medium (Scheme 9) [107]. By their approach, they have avoided the use of toxic reagents and solvents, harsh reaction conditions and long reaction times. The use of a heterogeneous catalyst and water made this method a green and sustainable approach for organic transformations.

**Scheme 10:**
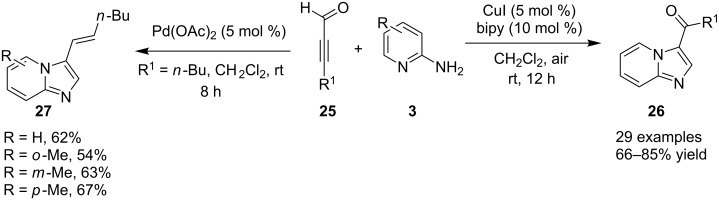
One-pot regiospecific synthesis of imidazo[1,2-*a*]pyridines.

This transformation involved the well known three-component reaction of **3** with **1** and **2** in an aqueous medium (Scheme 9). In order to increase the solubility of reactants in aqueous medium cationic surfactant (cetyl trimethylammonium bromide: CTAB) has been used which has also increased the yield of the product. Aliphatic aldehydes resulted in lower yields (65 and 76%) whereas dimethylamino- or nitro-substituted aldehydes did not result in the target compound that might be due to the deactivation caused by coordination of these groups with the catalyst. Metal–carbene complexes attracted the attention of organic chemists and have become an important branch of organometallic chemistry. Recently in 2014, metal–carbene complexes have been used as catalysts for the synthesis of imidazo[1,2-*a*]pyridines, via Cu(I) and Pd(II)-catalyzed cyclization (Scheme 10) [108]. The remarkable feature of this report was the metal carbene complex-catalyzed, one-pot process for the formation of C=O and C=C bonds to prepare functionalized imidazo[1,2-*a*]pyridines. Screening of the reaction conditions has proven the CuI–bipyridine (bipy) combination as the best catalyst–ligand symbiosis for this transformation at room temperature. In this synthesis, temperature and atmosphere both played the decisive role in product yield, as the reaction carried out at higher temperatures and under anaerobic atmosphere resulted in a trace amount of product. Differently substituted 2-aminopyridines **3** along with aryl and alkyl as well as terminal propiolaldehydes **25** were well tolerated.

**Scheme 11:**
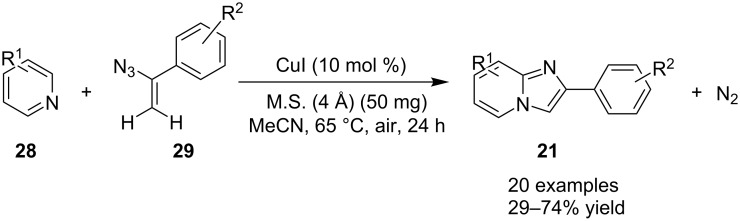
Vinyl azide as an unprecedented substrate for imidazo[1,2-*a*]pyridines.

The yield was >70% except for the products of 3-methyl- and 5-methyl-2-AP with propiolaldehyde. The protocol has also demonstrated a novel Pd(OAc)-catalyzed synthesis of 3-vinylimidazo[1,2-*a*]pyridines via 1,2-H shift of Pd–carbene complexes. This methodology has been considered as an efficient, one-pot regiospecific synthesis for a number of biologically active imidazo[1,2-*a*] pyridine derivatives [108]. Encouraged by the direct synthetic strategies for imidazo[1,2-*a*]pyridines (IPs), Donthiri et al. have reported an efficient Cu-catalyzed C–H functionalization of pyridines with vinyl azide derivatives [109]. Their use of vinyl azide derivatives **29** for the synthesis of IPs was unprecedented. In this strategy, vinyl azide acts as a source of nitrogen with the liberation of N_2_ as a benign byproduct under aerobic and mild reaction conditions (Scheme 11). The protocol has surpassed the use of 2-AP as one of the mostly used reactants and replaced it with pyridine **28**.

**Scheme 12:**
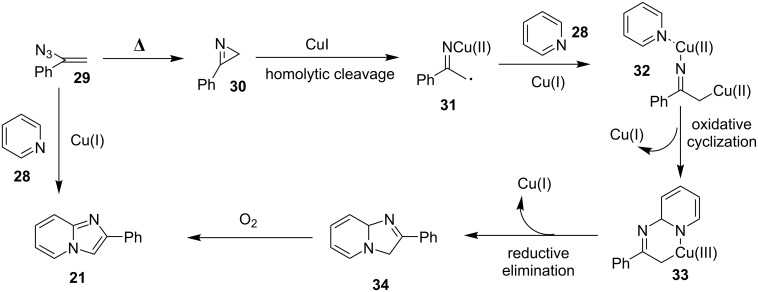
Radical pathway.

**Scheme 13:**
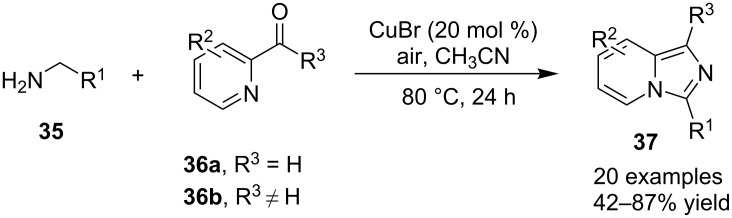
Cu(I)-catalyzed transannulation approach for imidazo[1,5-*a*]pyridines.

**Scheme 14:**
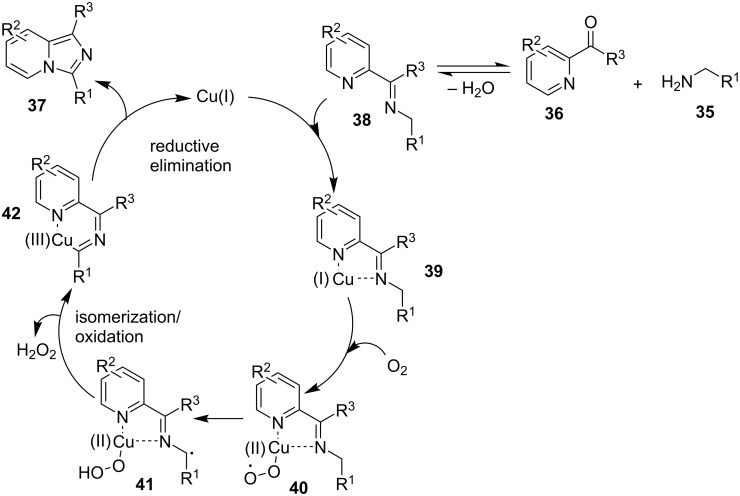
Plausible radical pathway for the synthesis of imidazo[1,5-*a*]pyridines.

The reaction proceeded through both radical and ionic pathways, yielding the products in moderate to good yields. The ionic pathway was similar to that reported by Yu et al. [110] on the other hand the radical pathway was supposed to occur as shown in Scheme 12. This involved generation of iminyl radical intermediate **31** by homolytic cleavage of the C–N bond which was followed by reductive elimination and oxidation to yield final compound **21**. Inspired by the work of Wang et al. [15] who have exploited a Cu(II)-catalyst for the construction of pyrido[1,2-*a*]benzimidazoles Li and Xie have described a Cu(I)-catalyzed direct transannulation of *N*-heteroaryl aldehydes/ketones **36a**,**b** with aryl/alkylamines **35** to form imidazo[1,5-*a*]pyridines **37** [11]. They have utilized aerial oxygen as a greener oxidant for oxidation during the reaction (Scheme 13). Mechanistically, the reaction involved the formation of radical intermediates, as use of TEMPO inhibited intramolecular C(sp^3^)–H amination of imine species. Aerial oxidation of the Cu(I) species bonded to the N-atom of pyridine and imine **39** resulted in Cu(II) superoxo radical intermediate **40**. This was followed by intramolecular hydrogen abstraction from the sp^3^ carbon atom leading to the formation of six-membered Cu(III) species **42**. Furthermore, consecutive isomerization/oxidation/reductive elimination leads to the generation of final compound **37** with regeneration of the Cu(I) catalyst (Scheme 14).

**Scheme 15:**
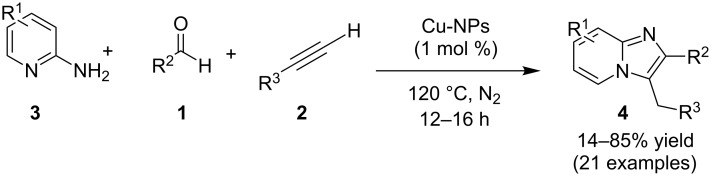
A solvent-free domino reaction for imidazo[1,2-*a*]pyridines.

**Scheme 16:**
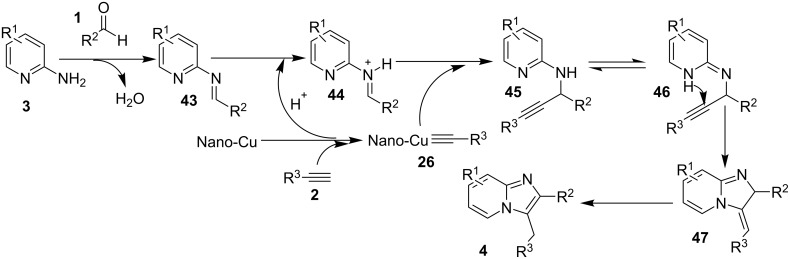
Cu-NPs-mediated synthesis of imidazo[1,2-*a*]pyridines.

**Scheme 17:**
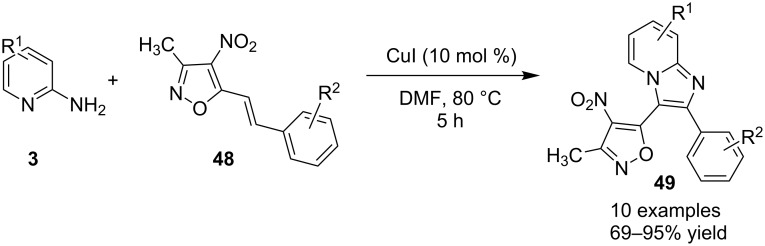
CuI-catalyzed synthesis of isoxazolylimidazo[1,2-*a*]pyridines.

**Scheme 18:**
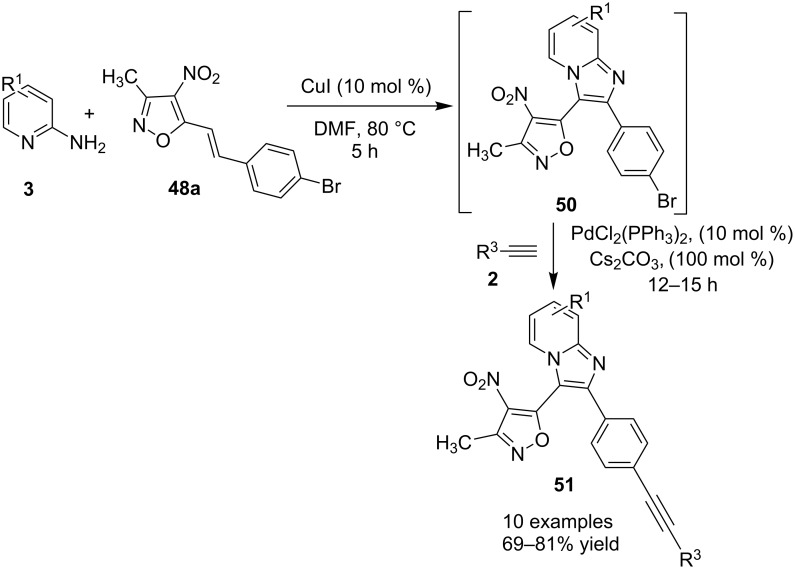
Functionalization of 4-bromo derivative via Sonogashira coupling reaction.

The presence of EDGs as compared to EWGs on N-heteroaryls gave a good yield. On the other hand, benzylamine has well tolerated both the EW and EDGs to give the products in appreciable yields. Moreover, various aliphatic amines irrespective of their steric hinderance resulted in appreciable yields of the product. Inspired from the excellent catalytic activity exhibited by Cu(0) NPs Chenglong et al. have reported an efficient, three-component one-pot reaction for the synthesis of imidazo[1,2-*a*]pyridines [111]. The protocol enjoyed a solvent-free domino reaction between compounds **3**, **1** and **2** under nitrogen atmosphere at 120 °C (Scheme 15). However, the reaction was unsuccessful with aliphatic and low-boiling aldehydes (as the reaction was taking place at high temperature). The reaction mechanism involved a Cu-NPs-mediated C–H bond activation of the alkyne which further reacted with the iminium ion to form propargylamine **45** and Cu NPs were again released into the system. The propargylamine formed went through 5-*exo-dig* cyclization to form IPs (Scheme 16). An unprecedented CuI-catalyzed two-component synthesis of isoxazolylimidazo[1,2-*a*]pyridines **49** was reported by the group of Rajanarendar under aerial conditions. Differently substituted 2-AP **3** and substituted nitrostyrylisoxazole **48** were used as reaction substrates at 80 °C (Scheme 17) [112]. The method has tolerated a variety of functional groups with good yield. Moreover, highly functionalized imidazo[1,2-*a*]pyridines have been synthesized by applying a Sonogashira coupling reaction to the product of 4-bromo-substituted nitrostyrylisoxazole **48a** (Scheme 18). The attractive feature was successful substitution of the bromo atom with terminal alkynes. Different alkynes applied gave a good yield of up to 81%. This reaction provided a direct and efficient method to produce highly functionalized imidazo[1,2-*a*]pyridines.

**Scheme 19:**
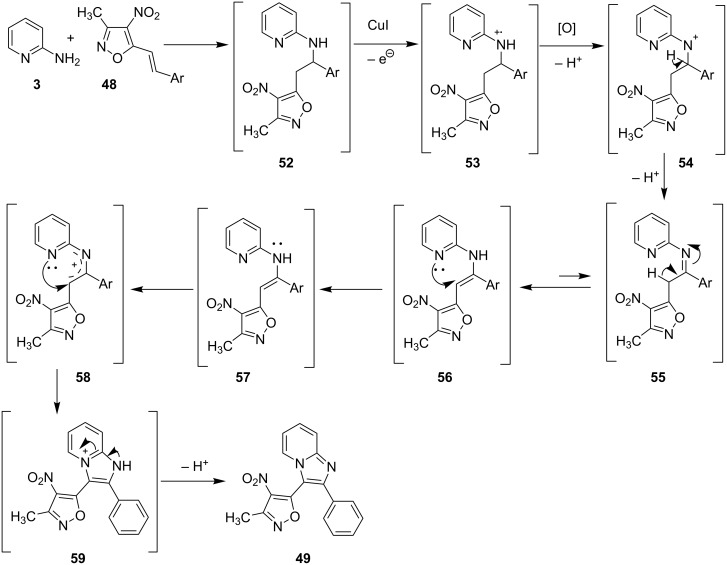
A plausible reaction pathway.

Mechanistically, the reaction proceeded by Michael type addition of 2-AP and nitrostyryl isoxazole. Further, a CuI-promoted one-electron oxidation followed by loss of a hydrogen radical and a proton, led to the formation of enamine **55**. The so formed enamine underwent single-electron transfer (SET) with CuI followed by hydride abstraction/intramolecular nucleophilic addition and loss of a proton forming the desired compound **49** (Scheme 19).

**Scheme 20:**
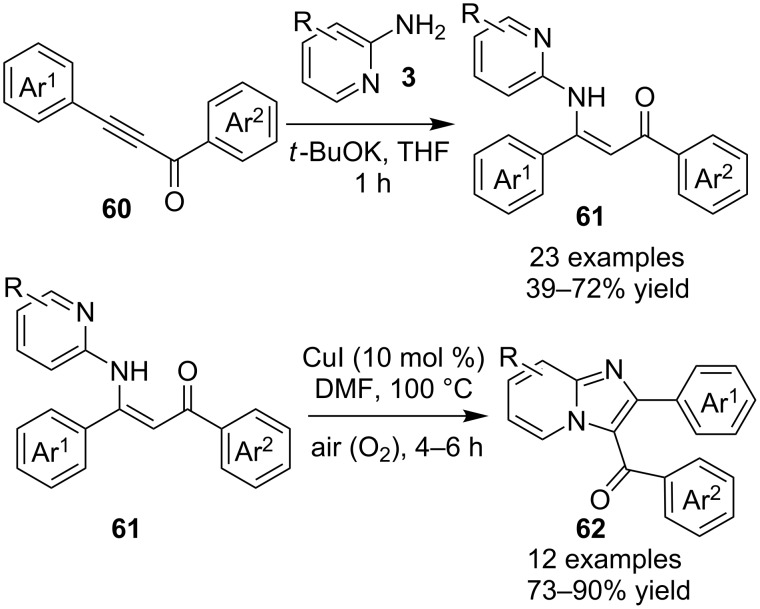
Cu(I)-catalyzed intramolecular oxidative C–H amidation reaction.

**Scheme 21:**
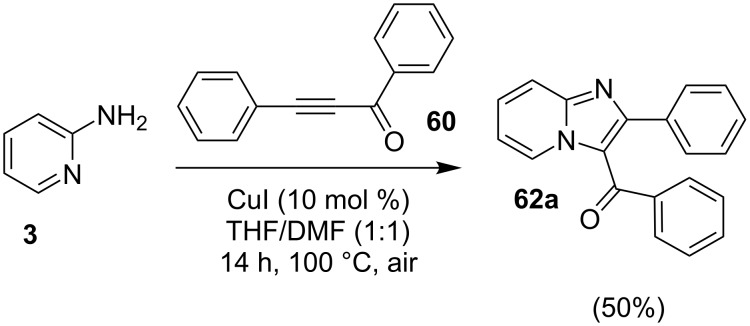
One-pot synthetic reaction for imidazo[1,2-*a*]pyridine.

**Scheme 22:**
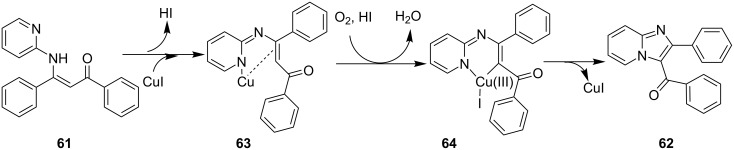
Plausible reaction mechanism.

**Scheme 23:**
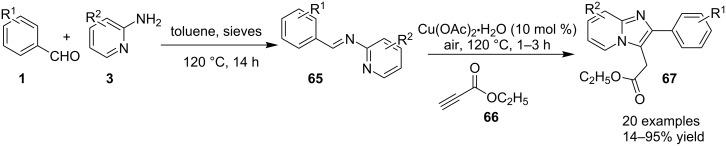
Cu(OAc)_2_-promoted synthesis of imidazo[1,2-*a*]pyridines.

Cu(I)-catalyzed intramolecular oxidative C–H amidation of *N*-pyridylenaminones **61** for the synthesis of imidazo[1,2-*a*]pyridine derivatives have been reported by the group of R. K. Reddy [113]. The protocol offered an open air, ligand- and base-free methodology with an extension towards the synthesis of imidazo[1,2-*a*]pyrazines/pyrimidines and benzo[*d*]imidazo[2,1-*b*]thiazoles. For this reaction, they have synthesized the respective enaminones **61** by conjugative addition of heteroarylamines **3** with α,β-ynones **60** (Scheme 20). Optimization of the reaction inferred that CuI with dimethylformamide (DMF) at 100 °C under air would be the best conditions for a maximum yield. The reaction viability was also tested without the isolation of product **61** in a one-pot fashion, by reacting 2-AP **3** and 1,3-diphenylprop-2-yn-1-one **60** under the given conditions. This one-pot methodology provided the final product with a yield of 50% (Scheme 21). For pyridine synthesis, both *N*-pyridyl as well as enone fragment have well tolerated a large number of functional groups. Also, the effect of steric congestion has been nullified as the product yield was up to 95%. Mechanistically, an intermediate complex was formed by coordination of the pyridine ring with Cu compound **63**. The intermediate thus obtained was further oxidized by oxygen (air) to the Cu(III) complex **64**, which on reductive elimination gave the final product **62** (Scheme 22). Also, Irina V. Rassokhina and others have employed Cu(OAc)_2_ for the synthesis of imidazo[1,2-*a*]pyridines under aerobic conditions (Scheme 23) [114]. They have performed aminomethylation and cycloisomerization of propiolates **66** with imines **65** for the first time, using aerobic conditions with a Cu(II) catalyst. EWGs on aldehydes gave a high yield of the product whereas the yield was decreased with EDGs (2,4-OMe, 4-Me, 5-Br, 5-Me and 4-Et ≤37%). Also, the reaction of unsubstituted benzaldehyde with substituted 2-AP gave poor yields (5-Cl, 5-Me; 14 and 23%, respectively) whereas no target compound was synthesized with unsubstituted 2-AP **3**.

**Scheme 24:**
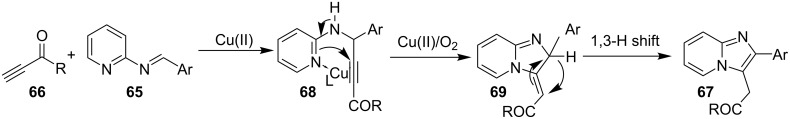
Mechanism for aminomethylation/cycloisomerization of propiolates with imines.

This methodology also provides a simple and concise route for the synthesis of the anxiolytic drug alpidem. The use of a Cu(I) catalyst was not fruitful for this synthesis. This might be due to the formation of Cu(II)-mediated propargylamine **68** from the alkyne and Schiff base-copper complex that was cyclized and aromatized to form the target compound **67** (Scheme 24).

**Scheme 25:**
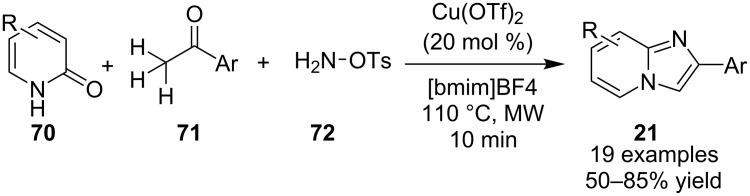
Three-component synthesis of imidazo[1,2-*a*]pyridines.

Kumar et al. have described a Cu(OTf)_2_-catalyzed three-component reaction for the synthesis of imidazo[1,2-*a*]pyridines under microwave irradiation [115]. 1-Butyl-3-methylimidazolium tetrafluoroborate ([bmim]BF_4_) was used as ionic liquid for this three-component reaction of pyridine-2(1*H*)-one **70**, acetophenone **71** and *o*-tosylhydroxylamine (**72**, Scheme 25). The reason behind the use of an ionic liquid as reaction medium was its environmentally benign nature, air stability and significantly its compatibility with various organic compounds.

**Figure 3:**
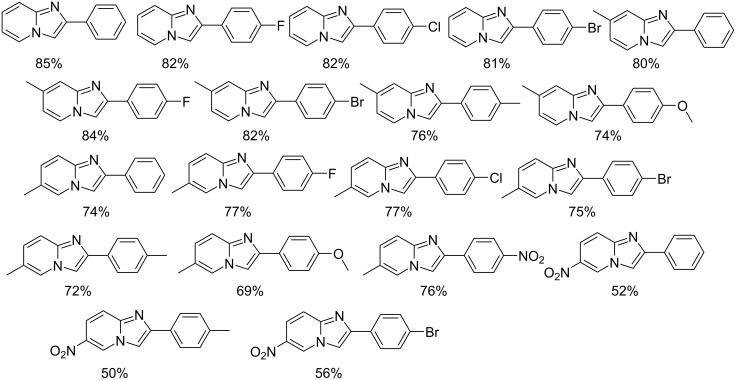
Scope of pyridin-2(1*H*)-ones and acetophenones.

This reaction was first of its kind in using pyridine-2(1*H*)-ones (precursor of 2-AP) directly for the synthesis of IPs. The generality of the present protocol lied in tolerance towards differently substituted reacting compounds. Acetophenones bearing EWGs reacted faster than those with EDGs as these groups might be unfavorable for the nucleophilic addition step during the reaction (Figure 3). The advantageous point of the protocol involves the recovery and reusability of the copper catalyst dissolved in ionic liquid to four synthetic cycles.

**Scheme 26:**
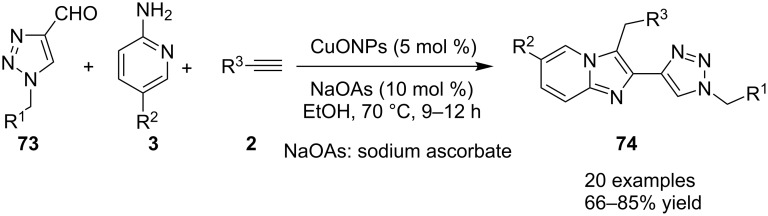
CuO NPS-promoted A^3^ coupling reaction.

Nanotechnology coupled with heterogeneous catalysis has emerged as an efficient field of catalysis for various organic transformations. Inspired from this Bagdi et al. have reported a nano-copper oxide-mediated three-component A^3^ coupling reaction for the synthesis of 2-triazolylimidazo[1,2-*a*]pyridine **74** (Scheme 26) [31].

**Scheme 27:**
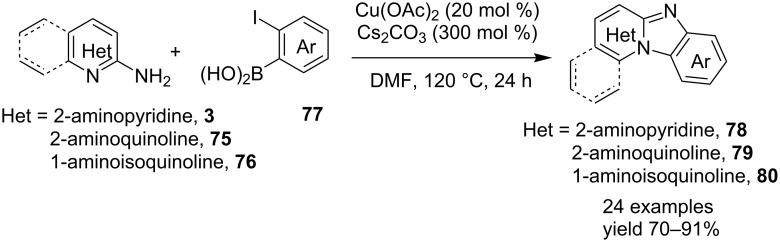
Cu(II)-catalyzed C–N bond formation reaction.

The reaction involved the use of copper oxide as a catalyst and sodium ascorbate as a reducing agent using triazolyl aldehyde **73**, amidine **3** and terminal alkynes **2** as reaction substrates at 70 °C (Scheme 26). Here sodium ascorbate (NaOAs) helped in the reduction of Cu(II) to Cu(I) which then reacted with the alkyne moiety. The species thus formed participated in the reaction by reacting with imine of aldehyde and amidine. The intermediate thus formed undergo 5-*exo-dig* cyclization and 1,3-H shift to form the final product. This was an unprecedented report for the synthesis of a library of targeted moieties using a nano-click catalyst. The triazole precursor was also synthesized by the group using nano-copper oxide in an aqueous medium. The reaction has well tolerated variedly substituted starting materials whether aromatic or aliphatic, only in case of triazoles substituted with an acetate group the final product was obtained in 66% yield. An open-flask, one-pot, Cu(II)-catalyzed ligand-free approach towards C–N bond formation was reported by Rasheed et al. [116]. The reaction was catalyzed by Cu(OAc)_2_ with cesium carbonate as a base under aerobic conditions. Along with the synthesis of pyrido[1,2-*a*]benzimidazoles **78**, they have reported the synthesis of benzimidazo[1,2-*a*]quinoline **79** and benzimidazo[1,2-*a*]isoquinoline **80** in good to excellent yields. They have used differently substituted arylboronic acids **77** as one of the common reactants with 2-aminopyridines/2-aminoquinolines/1-aminoisoquinolines **3**, **75**, **76** to give the desired products, respectively (Scheme 27).

**Scheme 28:**
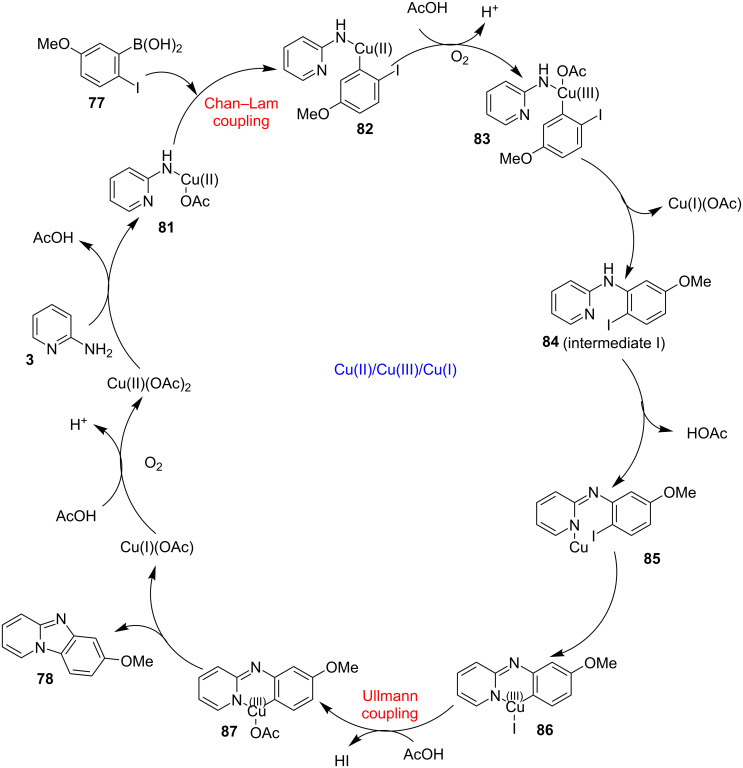
Mechanism involving Chan–Lam/Ullmann coupling.

Both EWGs and EDGs on the reactants resulted in good to excellent yields of the product. This process followed two types of coupling reaction mechanisms, i.e., a Chan–Lam coupling and an Ullmann coupling. The Chan–Lam coupling involved a C–N bond formation (intermediate **I**, **84**) which then entered into the Ullmann coupling to undergo intramolecular cyclization to form final product **78** and release Cu(III) to Cu(I) by reductive elimination. In this process oxygen (air) acts as oxidant which resulted in the oxidation of Cu(II) to Cu(III) and Cu(I) back to Cu(II) (Scheme 28).

**Scheme 29:**
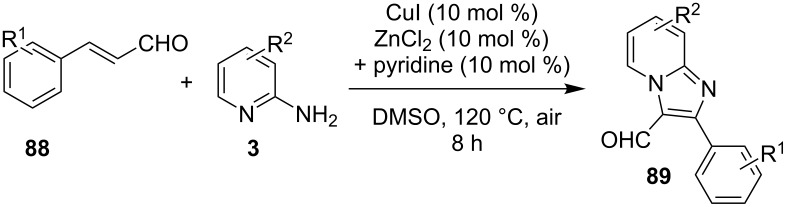
Synthesis of formyl-substituted imidazo[1,2-*a*]pyridines.

**Scheme 30:**
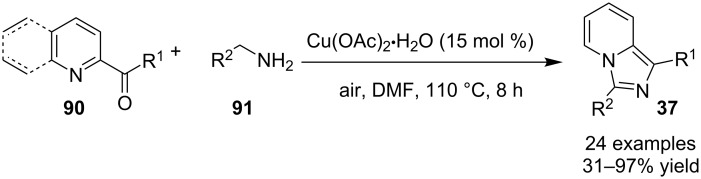
A tandem sp^3^ C–H amination reaction.

A copper-catalyzed oxidative cyclization approach has been used for the synthesis of formyl-substituted imidazo[1,2-*a*]pyridines **89**. The remarkable feature of this method was the preservance of the sensitive aldehyde group in the product structure, which can act as a precursor for the synthesis of some new motifs (Scheme 29) [117]. The reaction was carried out between 2-APs **3** and cinnamaldehyde derivatives **88** under aerobic conditions. ZnCl_2_ and pyridine act as additives, where ZnCl_2_ was supposed to promote the reaction rate by coordinating with the oxygen of the aldehyde. 2-APs with EWGs gave a higher yield of products than those substituted with EDGs while this thing got reversed with cinnamaldehyde (Table 1). 2-Methylbenzaldehyde and 5-chloro-2-aminopyridine resulted in the trace of final product, whereas the reaction was not completed with (*E*)-but-2-enal (entry 17 and 18, Table 1). Wang et al. have developed a Cu(II)-catalyzed tandem reaction between ketonic pyridine **90** and benzylamine **91** using DMF as a solvent at 110 °C, in the presence of oxygen as a clean oxidant (Scheme 30) [118]. Reaction optimization has shown Cu(OAc)_2_ to be suitable for this transformation whereas other Lewis acids like CuI, Cu(OTf)_2_ and FeCl_3_ gave lower yields, whereas Zn(OAc)_2_ did not result in any product. The strategy has well tolerated different substituents on pyridine and benzylamine as well, giving the corresponding products in excellent yields. This protocol served as a novel route for the synthesis of imidazo[1,5-*a*]pyridines **37** via oxidative amination of sp^3^ C–H bonds in the aerial atmosphere.

**Table 1:** Effect of substituents on product yield.

Entry	R^1^	R^2^	Yield (%)	Entry	R^1^	R^2^	Yield (%)

1	H	H	80	10	H	4-COOEt	78
2	H	3-Me	32	11	H	5-Br, 6-Me	52
3	H	5-Me	42	12	2-Me	H	66
4	H	6-Me	trace	13	3-Me	H	44
5	H	3-OCH_2_Ph	38	14	4-Cl	H	36
6	H	4-Cl	52	15	4-Br	H	32
7	H	5-Cl	88	16	2-NO_2_	H	38
8	H	5-Br	79	17	2-Me	5-Cl	trace
9	H	5-F	95	18	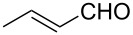	H	–

**Scheme 31:**
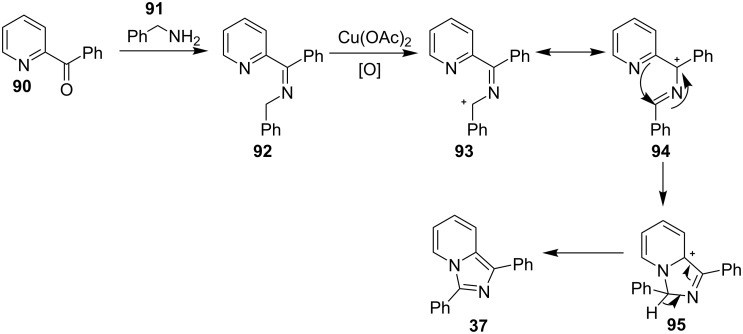
Probable mechanistic approach.

**Scheme 32:**
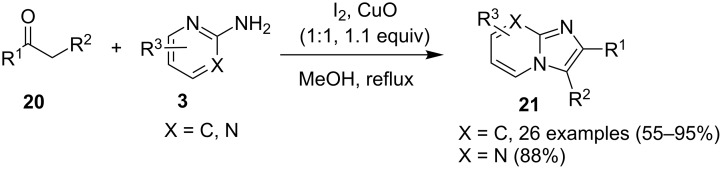
Dual catalytic system for imidazo[1,2-*a*]pyridines.

**Scheme 33:**
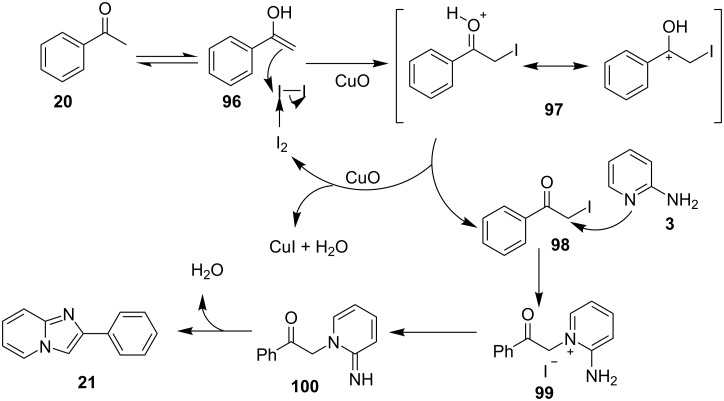
Tentative mechanism.

**Scheme 34:**
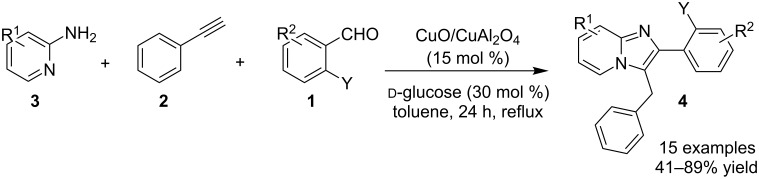
CuO/CuAl_2_O_4_/ᴅ-glucose-promoted 3-CCR.

The reaction involved oxidative dehydrogenation of benzylamine intermediate **92** to form **93** which underwent resonance to give **94**. This was followed by intramolecular amination, oxidative dehydrogenation, and rearrangement to yield the final product **37** (Scheme 31). A one-pot, tandem reaction promoted by a I_2_/CuO system to synthesize imidazo[1,2-*a*]pyridines was reported by Cai et al. (Scheme 32) [119]. In this reaction, iodine promoted the formation of iodo-intermediate **98** with a carbonyl compound that underwent nucleophilic substitution with 2-AP **3**. CuO played multiple roles in this reaction. Firstly it acts as an oxidizing agent to convert molecular iodine to the reactive iodonium ion (I^+^) species, secondly as a weak base to neutralize HI and next to reoxidize the iodide ion (I^−^) to molecular iodine (I_2_, Scheme 33). The reaction has enjoyed a broad substrate scope including arylmethyl, heteroaryl, α,β-unsaturated methyl ketones, β-ketone esters and 2-APs-substituted with EW and EDGs. The protocol also provided a concise route for the synthesis of the antiulcer drug zolimidine in 95% yield. A three-component coupling reaction (3-CCR) for the synthesis of N-fused pyridines was reported by Balijapalli and Iyer [40]. The reaction was catalyzed by CuO/CuAl_2_O_4_ and ᴅ-glucose. The reaction had been tried with various Cu(I)- and Cu(II)-based catalysts but the use of a mixed nano-CuO/CuAl_2_O_4_ and ᴅ-glucose system has resulted in appreciable yields. This three-component one-pot reaction involved 2-AP, phenylacetylene and substituted benzaldehydes as the starting substrates (Scheme 34).

**Scheme 35:**
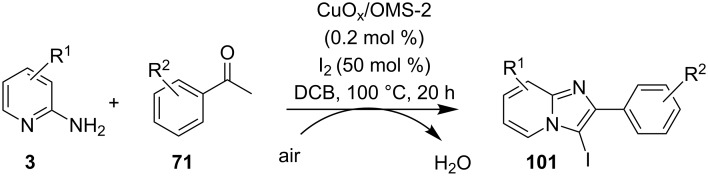
A tandem CuO*_x_*/OMS-2-based synthetic strategy.

The reaction mechanism comprises of an in situ generation of the imine from the aldehyde and 2-AP. Further, the addition of alkyne and activation by CuO(II)/Cu_2_O(I) yielded the A^3^-coupled propargyl amine [40]. Tautomerization/5-*exo-dig* cyclization led to the formation of the final product. The reaction enjoyed a rich library of compounds with good yields except 5-bromo, 4-methyl and 3-methyl substitution on 2-AP with 41, 45 and 49% yield, respectively. Meng et al. have used copper supported on manganese oxide-based octahedral molecular sieves OMS-2 (CuO*_x_*/OMS-2) as a heterogeneous catalytic system for a tandem synthesis of 3-iodoimidazo[1,2-*a*]pyridines (Scheme 35) [120]. The synthesis was similar to that reported by Kumar and co-workers with the difference of a heterogeneous catalytic system and iodination of the product [121]. Molecular iodine was used as an iodinating agent in the reaction.

**Figure 4:**
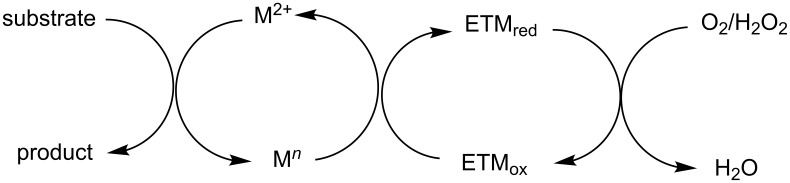
Biomimetic catalytic oxidation in the presence of electron-transfer mediators (ETMs).

In this reaction, OMS-2 acted as support and an electron transfer mediator for copper in order to generate a low energy pathway for rapid electron transfer thereby minimizing the catalyst loading (Figure 4). The reaction was supposed to proceed through iodine-catalyzed Ortoleva–King reaction with the assistance of Cu followed by CuO*_x_*/OMS-2-catalyzed electrophilic oxidative iodination.

**Scheme 36:**
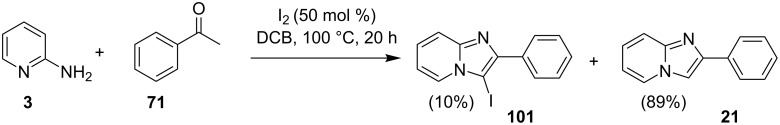
Control experiment.

The importance of catalyst for iodination was ascertained by the reaction in Scheme 36, which depicted that absence of CuO*_x_*/OMS-2 leads to imidazo[1,2-*a*]pyridine **21** as the major product rather than 3-iodo derivative **101**. The reaction has well tolerated differently substituted ketones, with lack of product in case of *ortho*-substitution due to steric hinderance. Similarly, substituted 2-APs provided a library of compounds with god yield, however, α,β-unsaturated ketones, such as benzalacetone, did not afford any desired product, perhaps because the α,β-unsaturated double bond affected the iodination.

**Scheme 37:**
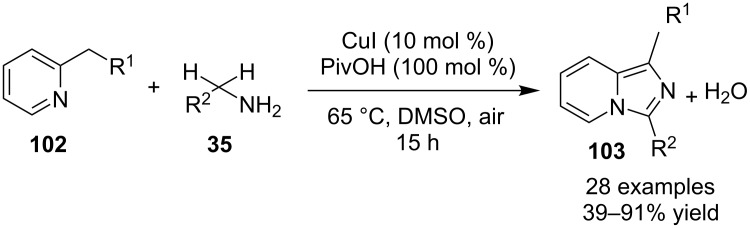
Copper-catalyzed C(sp^3^)–H aminatin reaction.

Mohan et al. successfully developed an efficient copper-catalyzed aerobic oxidative amination of C(sp^3^)–H bonds to synthesize imidazo[1,5-*a*]pyridine derivatives [122]. The reaction was also applicable to amino acid derivatives, as ethyl glycinate resulted in a 53% yield of the corresponding IP. The method was more atom economic and sustainable than others as it utilized air as a sole oxidant and generated water as the only byproduct. The reaction was carried out between pyridyl esters **102** and benzylamine (**35**) at 65 °C using pivalic acid as an additive in DMSO under open air (Scheme 37). Although the product yield was decreased when the reaction was performed in oxygen (balloon) atmosphere, might be due to the conversion of benzylamine to imine.

**Scheme 38:**
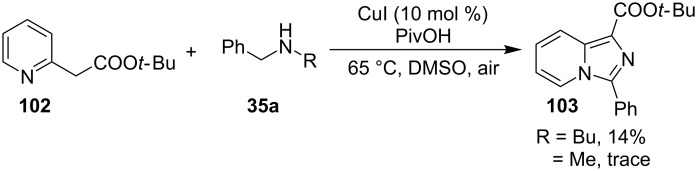
Reaction of secondary amines.

The reaction has beautifully tolerated a huge number of substrates with differently substituted pyridyl esters along with aromatic/heteroaromatic/aliphatic amines to give a good yield of products. However, steric hinderance was observed with 2-OMe-substituted benzylamine giving a trace amount of product. Moreover, 2-(pyridine-2-yl)acetonitrile and 2-picolylamine did not produce the desired product. The reaction with secondary amines **35a** also resulted in a trace of the final product (Scheme 38).

**Scheme 39:**
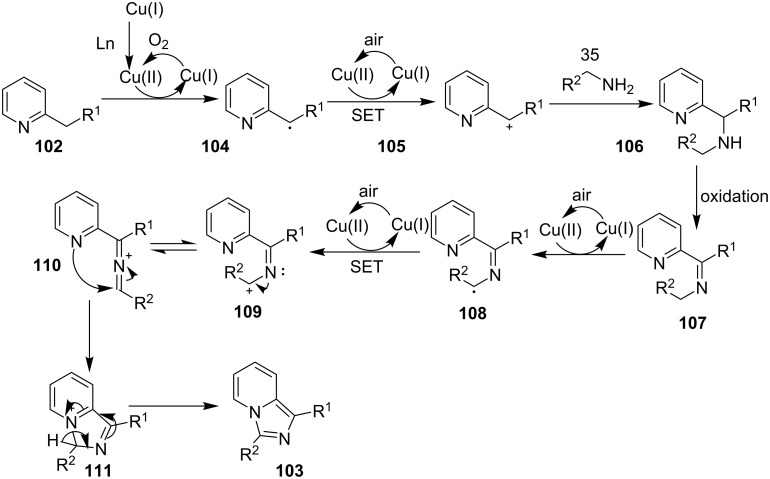
Probable mechanistic pathway.

**Scheme 40:**
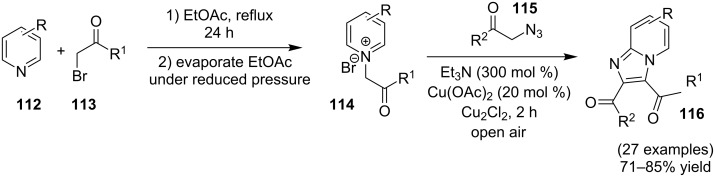
Coupling reaction of α-azidoketones.

**Scheme 41:**
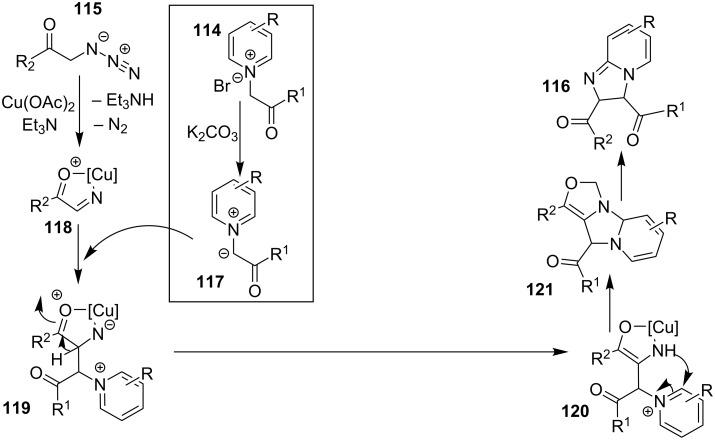
Probable pathway.

The use of TEMPO (a radical scavenger) has shown that the reaction might proceed through a radical pathway as depicted in Scheme 39. In the presence of TEMPO the desired product was not formed. Atmospheric oxygen has oxidized Cu(I) to Cu(II). This Cu(II) acts as an initiator for a single electron transfer (SET) process, to proceed the reaction with benzylamine. A regioselective synthesis of imidazo[1,2-*a*]pyridines was reported by the group of Kamal and Reddy [123]. They have performed a Cu(OAc)_2_-Et_3_N-mediated coupling reaction of α-azido ketones **115** with pyridinium ylides **114** using oxygen as a green oxidant (Scheme 40). The oxo-functionality present in α-azido ketones increased its acidity thus making it a good organic synthon. Optimization of the reaction conditions revealed that the presence of copper salt is mandatory for the formation of the fused heterocycle as its absence resulted in the formation of the enaminone only. The imines **118**, generated in situ via the loss of nitrogen from azide derivatives, were found to be reactive towards 1,3-dipoles **117** to form diverse heterocycles. Pyridinium ylides used as 1,3-dipoles in this protocol were synthesized by the reaction of pyridines with α-bromo ketones (Scheme 41). The protocol was applicable to a series of EW and EDGs on phenacyl azides. However, the reaction was not successful with ethyl azidoacetate. On the other hand, EW and EDGs were also well tolerated by pyridinium ylides.

The group of Bharate and Abbat have successfully reported a simple, efficient and excellent C–N bond formation catalyzed by CuBr [124]. The protocol involved the aerobic oxidative coupling of 2-APs with cinnamaldehyde to form 3-formyl-2-phenylimidazo[1,2-*a*]pyridines (Table 2). Before their work, only three reports of such a formylation have been reported in the literature which suffered from certain drawbacks like difficult to prepare starting materials, multistep reaction, high temperature and the use of expensive catalyst [125–127]. The reaction was initially tried with different catalysts like triflic acid, I_2_, salts of Pd, Fe, Co and Cu which did not lead to the formation of the final product except CuSO_4_ and CuCl_2_ which gave a moderate yield of the products. Further studies revealed CuBr to be the most effective with a maximum yield of 90% in ethanol at 60 °C with optimization reaction between 2-AP and cinnamaldehyde (Table 2, entry 1).

**Table 2:** Substrate scope for the synthesis of 3-formyl-2-phenylimidazo[1,2-*a*]pyridines.

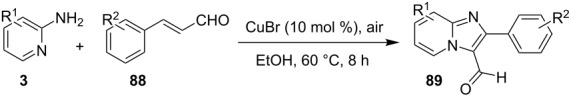							

Entry	R^1^	R^2^	Yield (%)	Entry	R^1^	R^2^	Yield (%)

1	H	H	90	8	5-Cl	H	80
2	3-Me	H	85	9	5-Me	4-OMe	78
3	H	4-Br	82	10	H	2-NO_2_	70
4	5-Me	4-Br	78	11	3-Me	2-NO_2_	72
5	5-Me	4-Cl	80	12	5-Me	2-NO_2_	75
6	H	4-OMe	82	13	5-Me	H	85
7	3-Me	4-OMe	75				

The reaction was carried out successfully under an air atmosphere rather than an inert atmosphere of nitrogen. This might be due to the oxidative nature of oxygen present in the air which was thought to be involved in hydrogen abstraction and oxidation of CuBr to Cu_2_O.

**Scheme 42:**
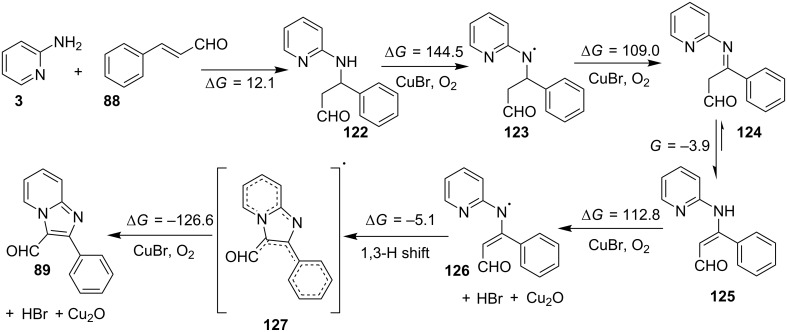
Probable mechanism with free energy calculations.

**Scheme 43:**
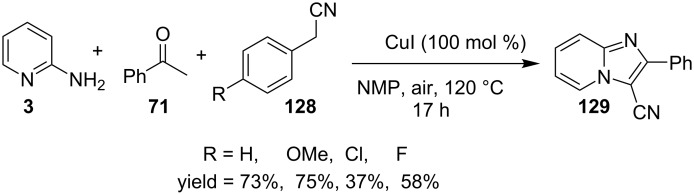
MCR for cyanated IP synthesis.

**Scheme 44:**
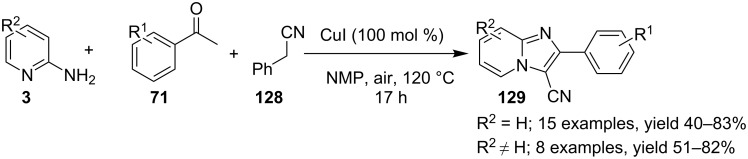
Substrate scope for the reaction.

The presence of EW and EDGs on both 2-APs and cinnamaldehyde supported the forward reaction whereas the reaction got failed with aliphatic aldehydes. The reaction mechanism involved the formation of radical intermediates and Cu_2_O as a byproduct which was marked by the change of color from initial green to red. The theoretical study was carried out to check the feasibility of the reaction by calculating Gibbs free energy difference for each step (Scheme 42). The capability of copper to catalyze the cyanation reaction was well exploited by Wen and Lu for a one-pot MCR for the synthesis of cyanoimidazo[1,2-*a*]pyridines **129** [128]. A variety of copper salts including CuI, CuBr, CuCl, Cu(OAc)_2_ and Cu_2_O were tried but only CuI gave some appreciable results. The product yield was improved by using *N*-methyl-2-pyrrolidone (NMP) as a solvent without any additive (Scheme 43). A good yield of the product was obtained by using benzyl cyanide substituted with EDG (4-OMe) rather than with an EWG due to the favourable oxidative release of cyanide anions from benzyl cyanide that favored the substitution of the iodide ion. Substituted aryl ketones, heteroaryl methyl ketones, and α,β-unsaturated methyl ketones were also well tolerated by the reaction (Scheme 44). The reaction was also successful in a two-step procedure in which the product of 2-AP and ketone can also be subjected to cyanation with benzyl cyanide thereby proving the viability of the reaction. In this reaction, benzyl cyanide was used as the source of cyanide ions.

**Scheme 45:**
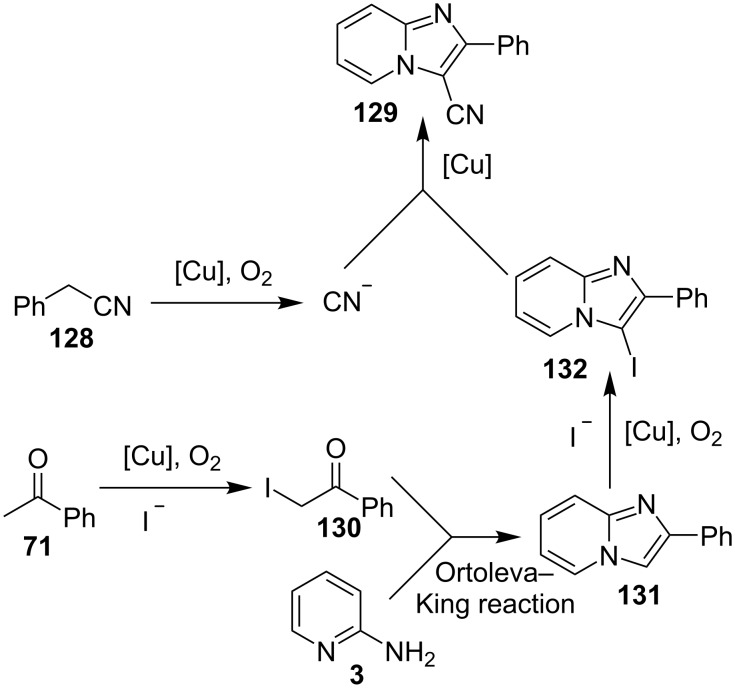
Reaction mechanism.

Mechanistically the reaction involved the simultaneous release of cyanide ions and the α-iodination of acetophenone catalyzed by CuI. Further, the Ortoleva–King reaction of the iodinated ketone **130** intermediate took place with 2-AP to afford imidazo[1,2-*a*]pyridine followed by iodination and cyanation to yield the final product **129** (Scheme 45). This method was superior in terms of high conversion yields, the formation of C–N bonds and C–CN bond in a concerted manner. Moreover, this approach was found to have commercial value since it is a good synthetic procedure for the synthesis of the drugs saripidem and necopidem in 62% and 59% yields, respectively. Cu supported on Zinc aluminate (ZnAl_2_O_4_) was unprecedentedly used for the three-component coupling reaction of aromatic aldehydes, 2-AP and phenylacetylene under nitrogen atmosphere (Table 3) [20]. In this protocol Cu was the active synthetic site and ZnAl_2_O_4_ acted as a support to provide a larger surface area and reduced the aggregation of Cu NPs. This protocol was better than reported by Chenglong et al. [111] in terms of low reaction temperature and lesser reaction time.

**Table 3:** Cu/ZnAl_2_O_4_-catalyzed 3-CCR.

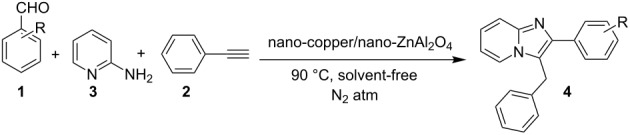			

Entry	R	Time (h)	Yield (%)

1	H	6	90
2	4-Me	4	89
3	4-OMe	4	89
4	4-Cl	4	94
5	4-F	4	94
6	2-F	4.3	/90

**Scheme 46:**
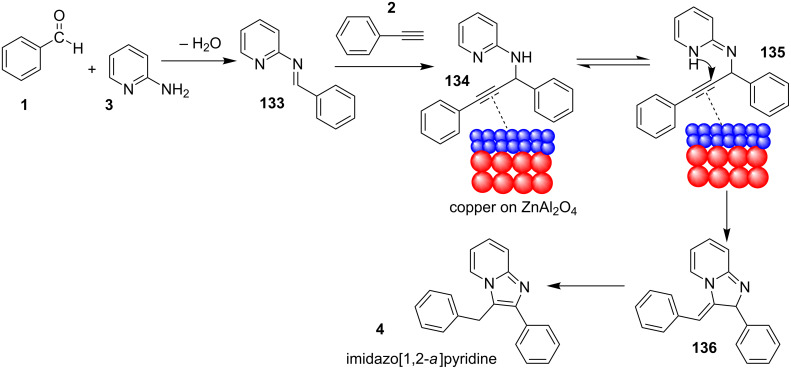
Probable mechanistic pathway for Cu/ZnAl_2_O_4_-catalyzed reaction.

Leaching of the metal ion was absent as tested by AAS, which ensured the recyclability of the catalyst to 5 synthetic cycles. Mechanistically the reaction proceeded by a nucleophilic attack of the alkyne on imine **133**, resulting in the formation of propargylamine **134**. The activation of the C–H bond in the alkyne by copper on ZnAl_2_O_4_ initiated an intramolecular nucleophilic attack of pyridine nitrogen to the triple bond (**135**). This was followed by aromatic isomerization to form imidazo[1,2-*a*]pyridines (Scheme 46). The importance of Cu for this reaction is mentioned in Table 4, where the absence of copper did not lead to the product formation (Table 4, entry 1).

**Table 4:** Scope of catalyst.

Entry	Catalyst	Yield (%)

1	ZnAl_2_O_4_	0
2	CuO	61
3	Cu powder	69
4	8 wt % Cu/nano-ZnAl_2_O_4_	90
5	8 wt % Ni/nano-ZnAl_2_O_4_	51
6	8 wt% Cu-Ni/nano-ZnAl_2_O_4_	55

**Scheme 47:**
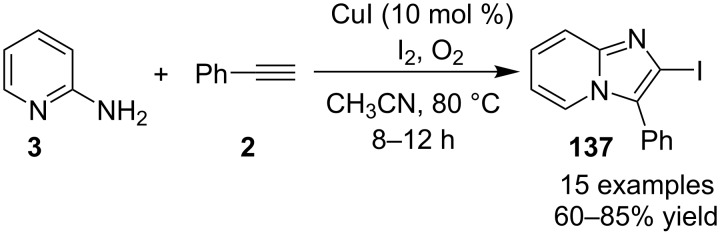
Copper-catalyzed double oxidative C–H amination reaction.

A double oxidative C–H amination reaction for the synthesis of 2-iodoimidazo[1,2-*a*]pyridines **137** was reported by Dheer et al. using a copper catalyst (Scheme 47) [129]. The synthesis of this moiety was also reported by the group of Gao in 2013, but it has the disadvantage of using special halogenating reagents for the synthesis of haloalkynes and also the tedious work-up procedure associated with it [44]. Application of another iodinating reagent like NIS proved to be futile under the reported conditions and also the absence of either CuI or I_2_ from the reaction did not result in the product formation.

**Scheme 48:**
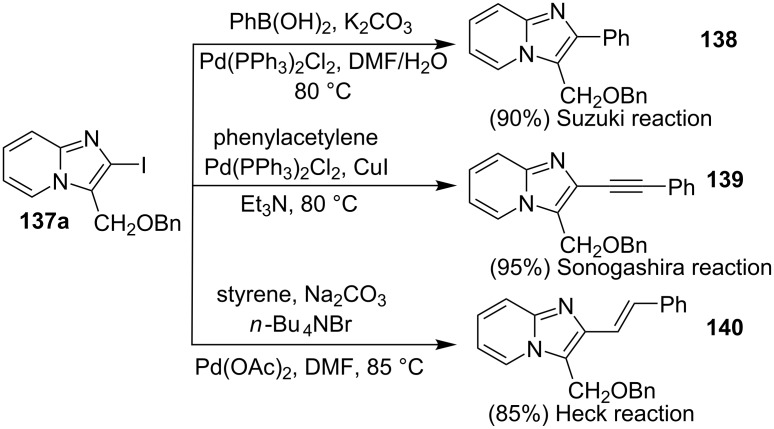
Application towards different coupling reactions.

All the reactions were performed under an oxygen atmosphere. Differently substituted 2-APs and terminal alkynes resulted in an appreciable yield of the product except 6-methyl-substituted 2-AP which did not form any product possibly due to steric hinderance. The advantageous part of this protocol was the synthesis of active pharmaceutical ingredients (API) that could be converted to commercial drugs sarpidem and nicopidem. Also, the compounds synthesized by this methodology could be successfully subjected to Suzuki, Sonogashira and Heck coupling reactions (Scheme 48).

**Scheme 49:**
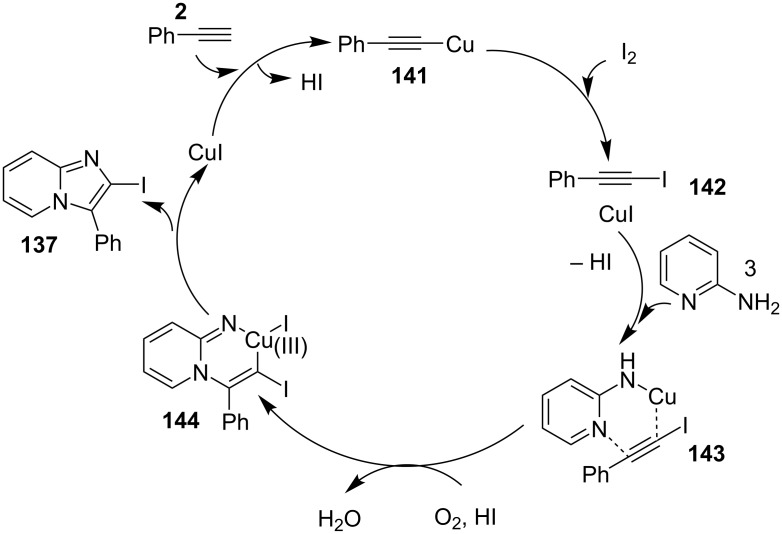
Reaction mechanism.

**Scheme 50:**
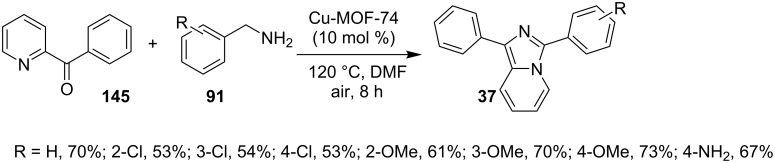
Condensation–cyclization approach for the synthesis of 1,3-diarylated imidazo[1,5-*a*]pyridines.

**Scheme 51:**
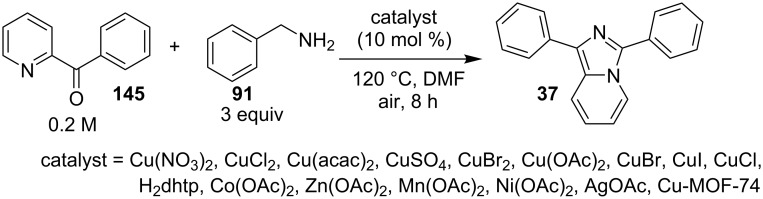
Optimized reaction conditions.

**Scheme 52:**
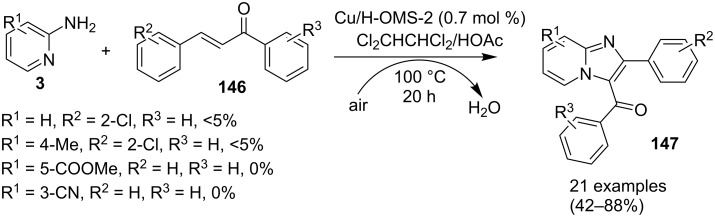
One-pot 2-CR.

**Scheme 53:**
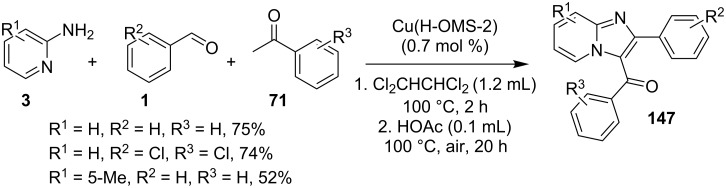
One-pot 3-CR without the isolation of chalcone.

The reaction proceeded by the formation of 1-iodoalkyne **142** which was characterized by mass and ^1^H NMR spetra. This iodoalkyne then formed a complex of Cu(I) with 2-AP. This was followed by migratory insertion of haloalkyne to form reactive Cu(III) intermediate **144** which underwent reductive elimination to give the desired product **137** and Cu(I) species (Scheme 49). The mechanism has shown that molecular iodine (I_2_) used in the reaction was the source of iodine in the final product rather than CuI. A condensation–cyclization reaction between 2-benzoylpyridine (**145**) and different benzylamines was carried out to synthesize 1,3-diarylated imidazo[1,5-*a*]pyridines (Scheme 50). The reaction took place under aerobic conditions utilizing Cu-MOF-74 as a catalyst [130]. The reaction was unprecedented in terms of oxidative amination of C(sp^3^)–H bond catalyzed by Cu-MOF-74. The group of Nguyen have deeply investigated the reaction and found 10 mol % loading of the catalyst with 3 equiv of benzylamine and 0.2 M 2-benzoylpyridine to be optimal for appreciable yield (Scheme 51). The reaction was optimized with different salts and Cu(OAc)_2_ was found to be effective for this transformation with a 78% yield, but it lacked reusability. They have experimentally demonstrated the importance of solid Cu-MOF for the reaction as there was not any contribution of leached active copper species to the formation of the desired compounds. Encouraged by their previous work Meng et al. have reported the aerobic synthesis of 3-aroylimidazo[1,2-*a*]pyridines **147** catalyzed by copper on H^+^-modified OMS-2 as a heterogeneous biomimetic catalyst [120,131]. They have successfully synthesized the desired compound by two systems viz., one-pot two-component reaction (2-CR) by using 2-AP and chalcone **146** (Scheme 52) and another by using a one-pot three-component reaction (3-CR) of 2-AP, aldehydes, and ketones (without the isolation of chalcone) (Scheme 53). The method has utilized a mixed solvent system comprising of Cl_2_CHCHCl_2_ (1,1,2,2-tetrachloroethane) and HOAc (acetic acid). Where HOAc was suitable for the formation of the Michael adduct and enhanced the electrophilicity of copper while Cl_2_CHCHCl_2_ helped in oxidative cyclization. However, the use of HOAc was not good for –COOMe and –CN substituted 2-AP as it led to their decomposition. A big library of compounds was constructed in which *ortho*-substituents gave lesser yields, possibly due to steric hinderance (Scheme 52). The advantageous feature of the process lied in the absence of base and ligand which makes it cost-efficient.

**Scheme 54:**
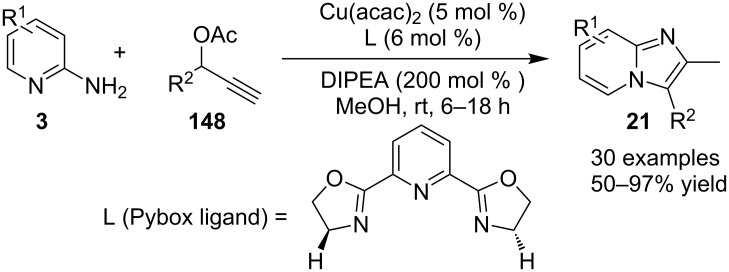
Copper–Pybox-catalyzed cyclization reaction.

Cheng et al. have reported a Cu(II)–Pybox-catalyzed reaction between 2-aminopyridines (2-APs) and propargyl alcohol derivatives bearing terminal alkyne substrates **148** to synthesize imidazo[1,2-*a*]pyridines (Scheme 54) [132]. The Pybox ligand was introduced by Nishiyama in the year 1989 [133]. The ligand consists of a pyridine ring surrounded by two oxazoline groups. Pybox ligands have the merit of the big binding site which could complex even with lanthanide cations and the increased rigidity associated with the tridentate Pybox scaffold [134]. 2-APs with EDGs like methyl and methoxy substitution at either *meta* or *para-*position gave good yield in relatively shorter reaction time (6 h) than those with EWGs like Cl/Br or CF_3_ (18 h). Moreover, the presence of EW/EDGs at 6-position of 2-AP did not afford the desired product due to the steric effect. In the case of propargyl alcohol derivatives, those with aliphatic substituent did not lead to the desired product. This reaction strategy has worked well up to gram scale level.

**Scheme 55:**
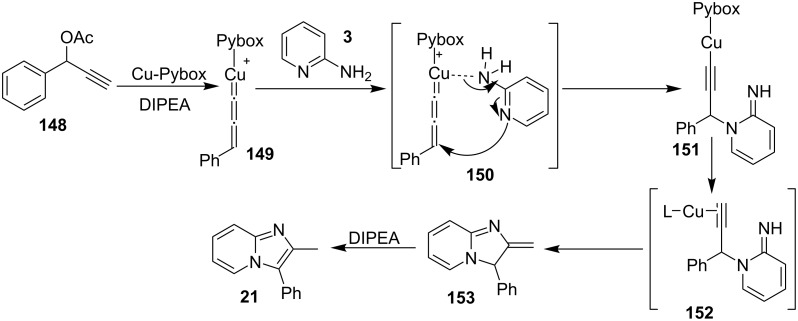
Mechanistic pathway catalyzed by Cu–Pybox complex.

**Scheme 56:**
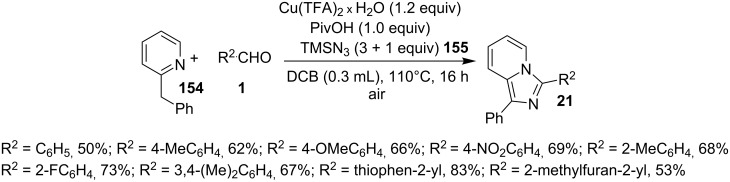
Cu(II)-promoted C(sp^3^)-H amination reaction.

**Scheme 57:**
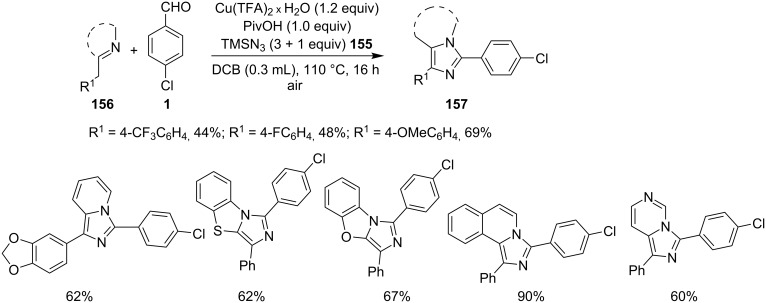
Wider substrate applicability for the reaction.

This reaction protocol has avoided the harsh reaction conditions like the use of high temperature and polar-aprotic solvents of a high boiling point such as DMF and DMSO. The added advantage of the method was regioselective propargylation at pyridine nitrogen **151**, followed by an intramolecular cyclization catalyzed by Cu–Pybox complex **152** (Scheme 55). Furthermore, the reaction was also utilized for gram scale synthesis of 2-methyl-3-phenyl-2,3-dihydroimidazo[1,2-*a*]pyridine, followed by its bromination and nucleophillic coupling for further derivatization. Xie et al. have reported a novel methodology via amination of the C(sp^3^)–H bond with TMSN_3_, catalyzed by Cu(II) salt to form imidazo[1,5-*a*]pyridines [135]. This three-component reaction involved benzyl-substituted N-heterocycles **154**, aldehydes **1** and TMSN_3 _**155** (Scheme 56), where heterocyclic nitrogen acted as both a directing group and an intramolecular nucleophile. The reaction was carried out under an inert atmosphere of argon with a total of 4 equiv of TMSN_3_ (3 + 1) to obtain the maximum yield of the desired product. Differently substituted benzaldehydes have been examined which showed good tolerance to EW as well as EDGs present at different positions. However, aliphatic aldehydes were failed to produce the desired product. Also, 2-benzylpyridines with EDGs afforded the product in higher yield than those substituted with EWGs. Other than pyridines this methodology gave good results with 2-benzylthiazole, 2-benzylbenzoxazole, and isoquinolines (Scheme 57).

**Scheme 58:**
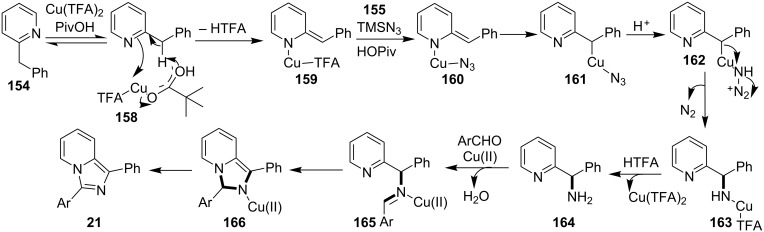
Plausible reaction mechanism.

**Scheme 59:**
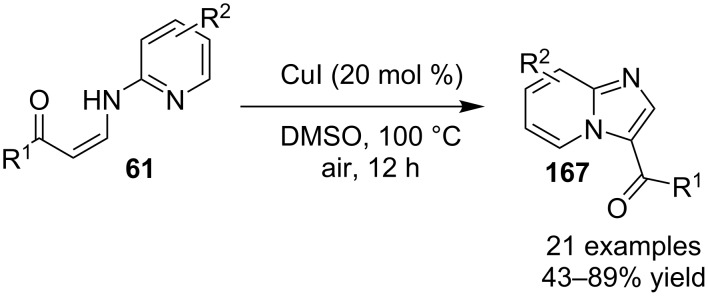
CuI assisted C–N cross-coupling reaction.

The authors were not sure about the exact mechanism thus based on previous reports it was believed to be propagated by the coordination of Cu(II) with the pyridinyl nitrogen **158** that facilitated deprotonation by the pivaloate anion to give an intermediate **159** [136–137]. This intermediate then involved the replacement of TFA by the azido group. The reaction was then expected to follow C–N bond formation, condensation with aldehydes, cycloaddition and finally, oxidative aromatization by Cu(II) to produce the desired product (Scheme 58). The C–N cross-coupling activity of copper was utilized by the group of Wan and Hu who have described a novel route for the synthesis of 2-unsubstituted imidazo[1,2-*a*]pyridines via intramolecular C–N bond forming cross-coupling reaction [138]. The reaction involved a copper-catalyzed C(sp^2^)–H amination. The reaction was carried out with *N*-pyridinyl secondary enaminones (**61**) [139] in the presence of CuI (20 mol %) in DMSO at 100 °C under open atmosphere (Scheme 59). The literature revealed that β-enaminones containing a free NH group and an additional β-substituent have been employed for the synthesis of 2,3-disubstituted imidazo[1,2-*a*]pyridines [140]. These enaminone-based protocols were not viable for the synthesis of imidazo[1,2-*a*]pyridine without 2-substitution. However, the protocol designed by Wan and Wen has successfully reported the synthesis of IPs without any substitution at the 2-position. The aryl ring associated with enaminones here has shown good functional group tolerance with moderate to excellent yields.

**Scheme 60:**
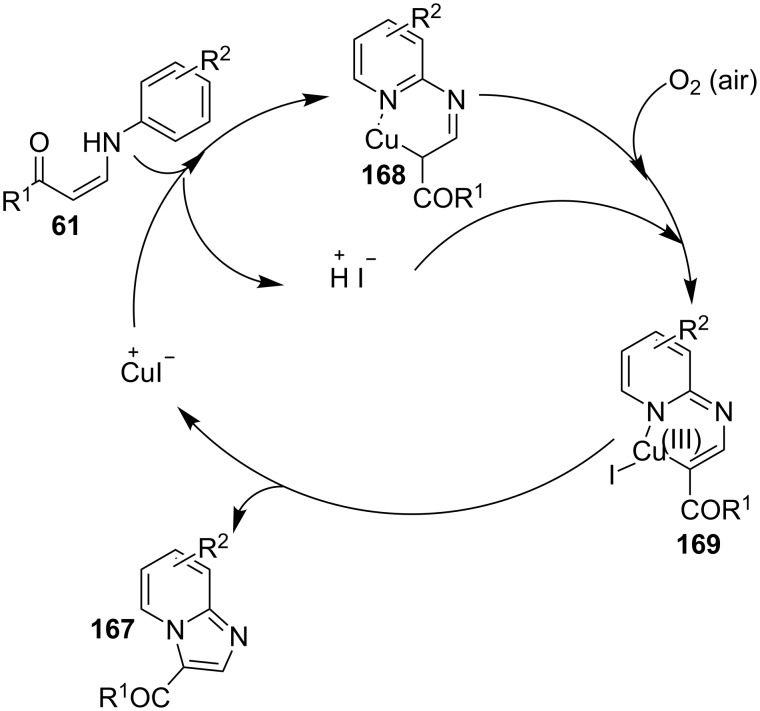
Probable reaction mechanism involving sp^3^ C–H amination.

**Scheme 61:**
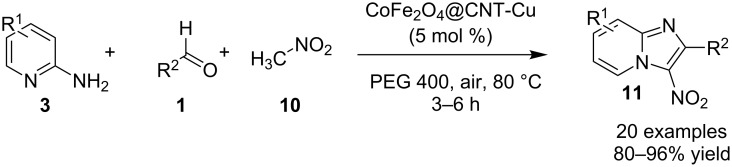
One-pot MCR-catalyzed by CoFe_2_O_4_/CNT-Cu.

**Scheme 62:**
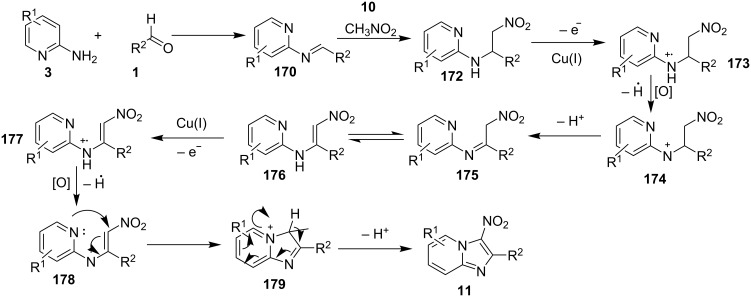
Mechanistic pathway.

On the other hand, the product yield was reduced with EWGs on the pyridine ring. Mechanistically, the pyridine nitrogen from enaminone acted as a chelating site to form a cuprous intermediate **168**. Oxidative addition of an iodide ion (I^−^) to this cuprous intermediate resulted in the Cu(III) intermediate **169** in the presence of air (O_2_). Reductive elimination of this intermediate resulted in the target compound with the release of Cu(I) which further participated in the catalytic process (Scheme 60). Inspired from these findings Zhang et al. have described the synthesis of imidazo[1,2-*a*]pyridines as a one-pot-3-CR [141]. A similar kind of reaction was also reported by Puthiaraj and co-workers, with Cu(BDC) as a heterogeneous catalyst and DMF as solvent [100]. The reaction reported by Zhang et al. between **3**, **1** and **10** was catalyzed by magnetic carbon nanotube supported Cu NPs (CoFe_2_O_4_/CNT-Cu) as an efficient magnetic heterogeneous catalyst (Scheme 61). Previously, the group has reported CuFeO_2_-catalyzed syntheses of imidazo[1,2-*a*]pyridines [142]. The reaction system has well tolerated EW and EDGs giving a good product yield of 80–96%. The Cu was inferred to play a major role in catalyzing the reaction as was explained by the mechanistic approach of the reaction. The reaction involved a Michael addition of nitromethane to imine intermediate **170**, which underwent loss of an electron and hydride ion twice, to form a cationic intermediate **178**. This cation then suffered nucleophilic addition and proton elimination to produce the final compound (Scheme 62).

**Scheme 63:**
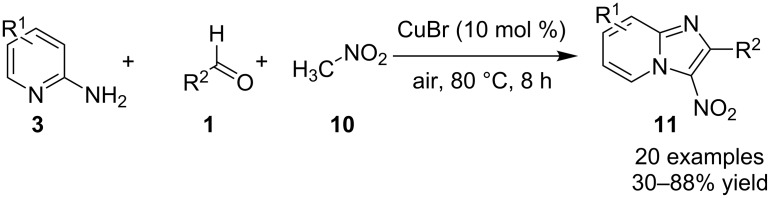
Synthetic scheme for 3-nitroimidazo[1,2-*a*]pyridines.

**Scheme 64:**
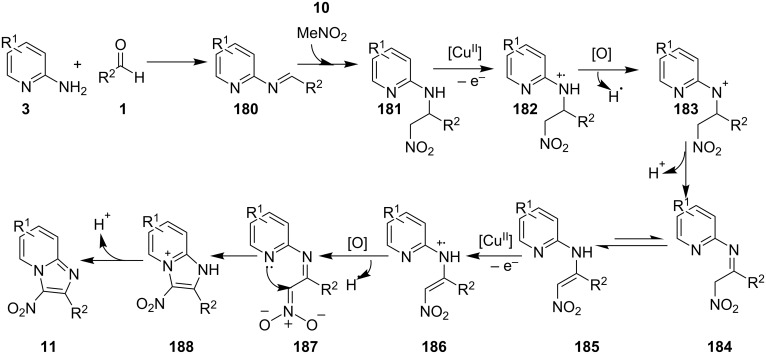
Plausible mechanism for CuBr-catalyzed reaction.

The beauty of the process lies in the reusability of the catalyst up to eight synthetic cycles along with the reusability of the PEG-400 which was found to be an optimal solvent for the reaction. A similar reaction was reported initially by the group of Yan and Yan who have utilized CuBr as a catalyst at 80 °C under aerobic conditions (Scheme 63) [143]. This reaction has tolerated a limited number of substituents where aldehydes with EDGs gave good yields as compared to EWGs, moreover, groups such as *o*-NO_2_, *p*-NO_2_, and CN resulted in a complex mixture and not the desired compounds. 2-APs also gave a similar outcome with a low yield of about 58% with the presence of an *ortho* substituent on it. However different 2-aminoheterocycles (2-aminopyrimidine/2-aminobenzimidazole) and aliphatic aldehydes viz., *n*-butyraldehyde and isobutyraldehyde were not found to be suitable substrates for this reaction. The reaction was thought to proceed via subsequent radical-cation intermediates, hydride abstraction and finally nucleophilic addition to give the desired product (Scheme 64). In the reported mechanism, the Cu catalyst was supposed to help in the formation of radical cation by taking up an electron in each of the two intermediate stages.

**Scheme 65:**
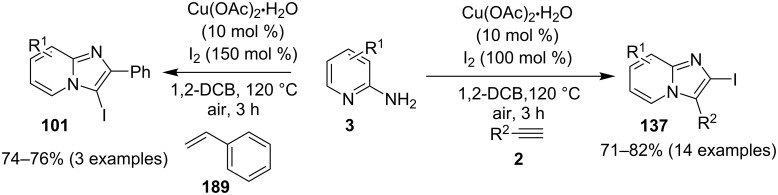
Regioselective synthesis of halo-substituted imidazo[1,2-*a*]pyridines.

In order to develop an easy and economic method for regio-divergent synthesis of halosubstituted imidazo[1,2-*a*]pyridines, Samanta et al. have reported a copper-catalyzed regioselective synthesis of 2- and 3-iodoimidazo[1,2-*a*]pyridines **137** and **101** [144]. Binucleophilic 2-AP and alkenes **189**/alkynes **2** in the presence of iodine and air were utilized as starting substrates (Scheme 65).

**Scheme 66:**
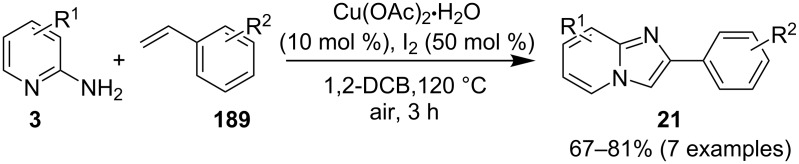
Synthesis of 2-phenylimidazo[1,2-*a*]pyridines.

**Scheme 67:**
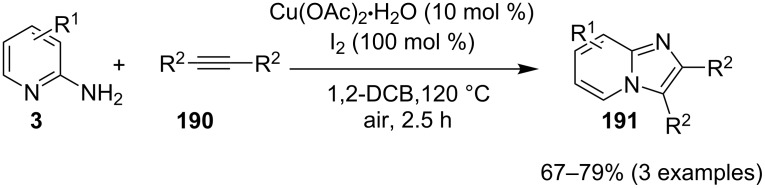
Synthesis of diarylated compounds.

Coupling of 2-APs with terminal alkynes resulted in 2-iodoimidazo[1,2-*a*]pyridines as the only products, the yield was quite appreciable with aromatic/aliphatic as well as heteroaromatic alkynes. However, coupling with alkene (styrene) resulted in 3-iodoimidazo[1,2-*a*]pyridines (150 mol % I_2_) and by varying the amount of iodine to 50 mol % 2-phenylimidazo[1,2-*a*]pyridines were obtained as the final product without iodine substitution (Scheme 66). The beauty of the process lied in the syntheses of different products just by varying the amount of iodine. Interestingly the use of symmetrical internal alkynes **190** resulted in the formation of 2,3-diarylimidazo[1,2-*a*]pyridines in good yield **191** (Scheme 67). Whereas, the inseparable regioisomeric mixture was obtained with unsymmetrical internal alkynes and no product was obtained with 4-octyne and 1-phenyl-1-propyne.

**Scheme 68:**
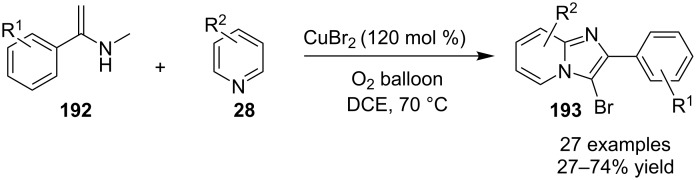
CuBr_2_-mediated one-pot two-component oxidative coupling reaction.

A copper-mediated aerobic oxidative coupling of pyridines **28** and enamides **192** was carried out in a one-pot fashion for the synthesis of 3-bromo-imidazo[1,2-*a*]pyridines **193** [145]. These 3-bromo substrates can be used as versatile synthetic blocks for further transformations. In this protocol CuBr_2_ act as a catalyst as well as a brominating agent. The reaction was carried out at 70 °C using dichloroethane (DCE) as a solvent under the atmosphere of oxygen which acted as an oxidant (Scheme 68).

**Scheme 69:**
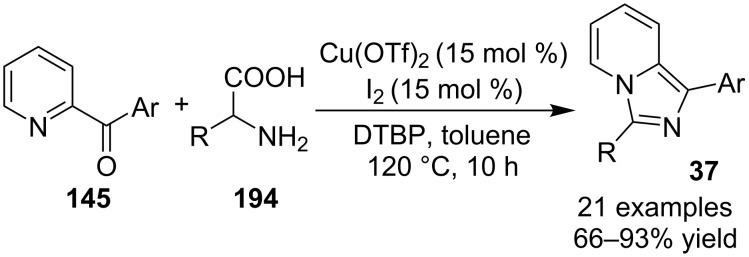
Decarboxylative cyclization route to synthesize 1,3-diarylimidazo[1,5-*a*]pyridines.

**Scheme 70:**
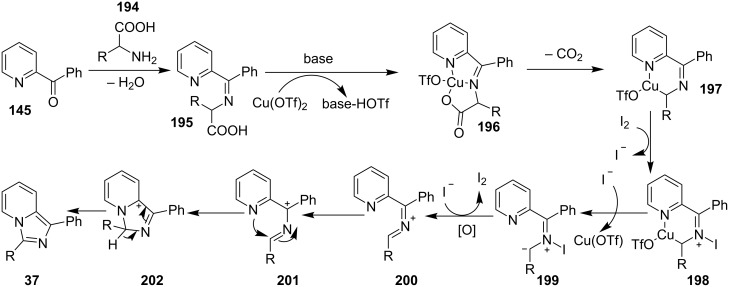
Mechanistic pathway.

This strategy displayed high functional group tolerance at the aromatic ring of the enamides, however, aliphatic enamides were not found to be suitable. The presence of a furan and thiophene ring in place of the benzene ring gave a product yield of only 27 and 44%, respectively. EW as well as EDGs present at *meta*-position of pyridine, gave good results as compared to the methyl group present at *o*- and *p*-positions which did not result in product formation. The overall yield of the products in this reaction was not much appreciable, also the product of *meta*-methyl- and *meta*-fluoro-substituted pyridines were obtained in an 1:1 isomeric mixture. Application of TEMPO (a radical scavenger) did not yield the desired product indicating the existence of a radical intermediate in the mechanism. This radical intermediate resulted in the formation of a carbocation which undergoes a nucleophilic attack by pyridine followed by isomerization of the so formed intermediate. The reaction then proceeded through a number of subsequent steps viz., intramolecular cycloaddition, hydrolysis, oxidative aromatization, and bromination thus yielding the final product. A copper/iodine co-catalytic system has been found to synthesize 1,3-diarylimidazo[1,5-*a*]pyridines **37** by decarboxylative cyclization of α-amino acids **194** with 2-benzoylpyridines **145** (Scheme 69) [146]. The reaction was supposed to obey an ionic pathway rather than a radical pathway. Mechanistically, decarboxylation step **196** was followed by oxidative iodination which was followed by elimination of iodine, intramolecular amination, dehydrogenation and rearrangement to yield the final product **37** (Scheme 70).

The reaction remained unaffected by the nature of substrates on the phenyl ring of 2-benzoylpyridines. Both EW and EDGs demonstrated excellent efficiency, only the *o*-substitution led to a decrease in the yield. However, with 1-(pyridin-2-yl)ethanone as the substrate, no corresponding product was detected. Assessment of the amino acids has shown that linear, branched and α-aryl-substituted amino acids were excellent reaction substrates. This method provided an effective alternative for the synthesis of 1,3-diaryl and 3-alkyl-1-arylimidazo[1,5-*a*]pyridines which were difficult to synthesize by the existing methods.

**Scheme 71:**
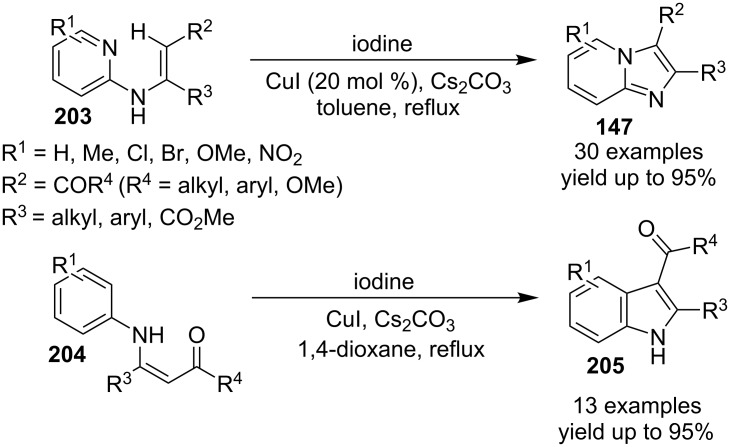
C–H functionalization reaction of enamines to produce diversified heterocycles.

**Scheme 72:**
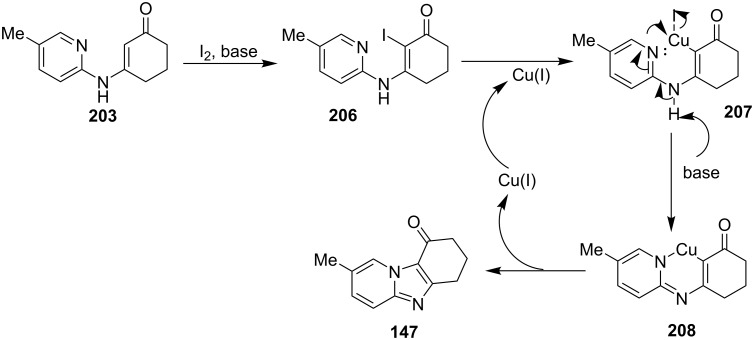
A plausible mechanism.

**Scheme 73:**
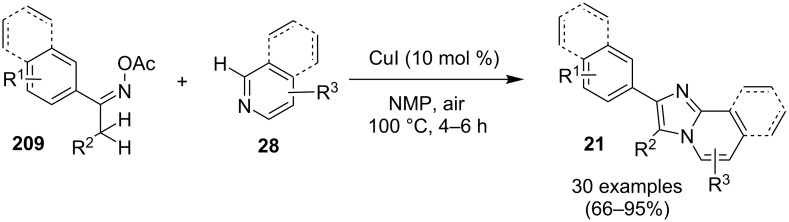
CuI-promoted aerobic oxidative cyclization reaction of ketoxime acetates and pyridines.

An efficient CuI/I_2_-mediated direct C–H functionalization reaction, compatible with both N-heteroaryl and N-aryl-substituted enamines **203** and **204** has been established to produce imidazo[1,2-*a*]pyridine **147** and indole derivatives **205** (Scheme 71) [147]. The reaction was feasible with both EDGs and EWGs present on the pyridine ring. The strategy was applicable in the synthesis of 2-methyl-3-acetyl derivatives of IP from 5-methylpyridin-2-amine and acetylacetone with no isolation of unstable condensation intermediate. Gratifyingly, the reaction worked well without the purification of crude enamines, produced by the reaction of arylamines and ketones. Moreover, no requirement of preliminary activation of the reaction centers, initiation of substrates, and reduced waste product generation are among some of the associated benefits of this strategy. In this reaction, I_2_ resulted in the formation of β-iodoenamide **206**, that underwent oxidative addition with Cu(I) followed by cyclization to form a metal complex **208** followed by reductive elimination to yield the final product (Scheme 72). A synthetic methodology for imidazo[1,2-*a*]pyridines by aerobic oxidative cyclization of ketoxime acetates **209** with pyridines or fused pyridines have been developed by the group of Ren and Zhao [148]. The reaction was catalyzed by CuI under mild conditions (Scheme 73). Ketoximes are valuable chemicals that are readily accessible through condensation of ketones with hydroxylammonium salts under mild conditions. Different copper salts have been tested for this reaction and Cu(II) salts were found to be inactive. With this methodology, they have successfully constructed a large library of compounds having both electron rich as well as deficient ketoxime acetates bearing methyl/methoxy/halogen substituents along with substituted pyridines. However, with 3-methyl and 3-halo-substituted pyridines two regioisomers were obtained in each case where 8-substituted imidazopyridines were the major isomer.

**Scheme 74:**
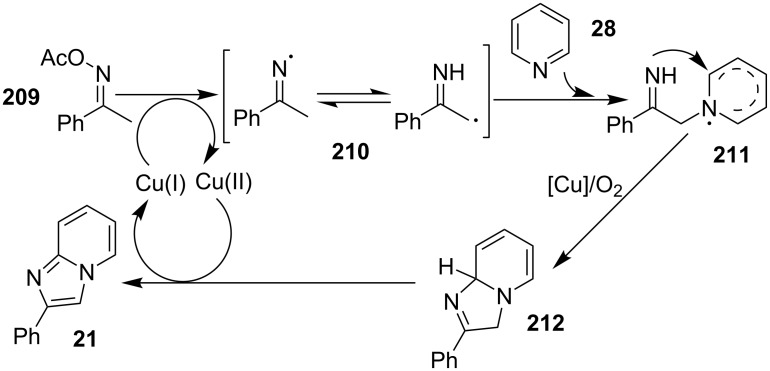
CuI-catalyzed pathway for the formation of imidazo[1,2-*a*]pyridine.

This protocol was also successful in synthesizing the antiulcer drug zolimidine in 81% yield on 10 mmol scale. The group has proposed a tentative mechanism, where CuI was involved in the reduction of ketoxime ester to the iminium radical **210**, which rapidly isomerized to the α-carbon radical. Subsequently, the radical formed get coupled with pyridine followed by intramolecular cyclization and oxidation in the presence of Cu/O_2_ to generate the final product (Scheme 74).

**Scheme 75:**
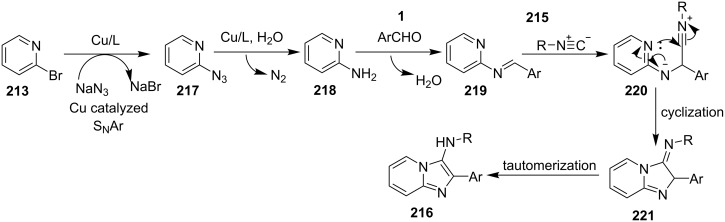
Mechanistic pathway.

An efficient copper-catalyzed four-component reaction was reported by Allahabadi et al. for the synthesis of imidazo[1,2-*a*]pyridines [149]. The novelty of the methodology lied in their use of 2-bromopyridine as one of the starting reactants rather than 2-AP (Table 5). Optimization of the reaction conditions has shown ʟ-proline to be the best out of different ligands used viz. 1,10-phenanthroline, pipecolic acid and ʟ-proline. To develop the best catalytic reaction conditions different copper salts were screened and CuI was found to be most effective using DMSO as solvent at 110 °C. The reaction mechanism involved Cu-catalyzed reductive amination of 2-bromopyridine with sodium azide to gave 2-AP. This 2-AP underwent imine formation with aldehyde which was followed by nucleophilic addition of the isocyanide. This step was followed by intramolecular cyclization **220** and tautomerization to produce the targeted molecule (Scheme 75). An unprecedented work for the synthesis of 2-haloimidazo[1,2-*a*]pyridines in terms of alkynoic acid as one of the reaction substrate was reported in the year 2017 by the group of Liu and Wang [150]. They have successfully reported the two-component, Cu(II)-promoted one-pot reaction of 2-AP and alkynoic acid **221** for the synthesis of IPs (Table 6). Before this report, three synthetic routes have been reported which suffered from certain bottlenecks like the use of prefunctionalized haloalkynes, limited substituent type at the C-3 position and limited halogenated starting material [44,129,144,151]. The two-component reaction did not require the presence of any base, as initial use of basic reagents like pyridine, Et_3_N, K_2_CO_3_ or Cs_2_CO_3_ resulted in a trace amount of final compounds. Moreover, the reaction did not proceed in the absence of CuBr_2_ and also the use of NaBr as brominating agent (to reduce catalyst loading) did not give satisfactory results. Air or oxygen atmosphere was required for successful completion of the reaction whereas use of oxidants such as TBHP/DTBP resulted in a decrease in the product yield. Alkynoic acids were easier to handle than the corresponding terminal alkynes and haloalkynes, the reason is their high boiling point. The reaction was not affected by steric and electronic factors as a wide variety of substituents along with aliphatic acids resulted in moderate to good yields of the products. Apart from copper bromide, its chloride salt also acted as good halogenating agent for synthesizing 2-chlorinated compounds (Table 7).

**Table 5:** Copper-catalyzed 4-CR for the synthesis of imidazo[1,2-*a*]pyridine.

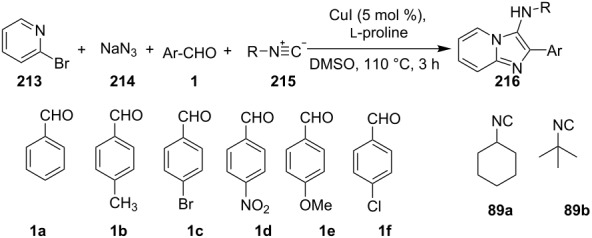					

Entry	Substrates	Yield (%)	Entry	Substrates	Yield (%)

1	**3a** + **4a**	78	6	**3f** + **4a**	81
2	**3b** + **4a**	81	7	**3a** + **4b**	88
3	**3c** + **4a**	79	8	**3f** + **4b**	84
4	**3d** + **4a**	82	9	**3b** + **4b**	84
5	**3e** + **4a**	76	10	**3c** + **4b**	85

**Table 6:** Cu(II)-promoted one-pot reaction for synthesizing IP.

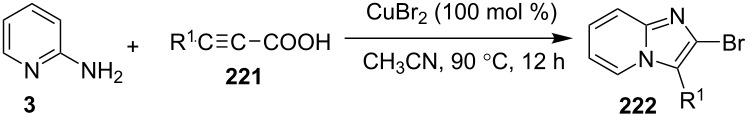					

Entry	R^1^	Yield (%)	Entry	R^1^	Yield (%)

1	C_6_H_5_	78	7	3-OMeC_6_H_4_	83
2	4-FC_6_H_4_	72	8	2-ClC_6_H_4_	70
3	4-ClC_6_H_4_	75	9	H	46
4	4-MeC_6_H_4_	85	10	Me	50
5	4-EtC_6_H_4_	82	11	Et	56
6	4-OMeC_6_H_4_	86	12	*n*-Pr	63

**Table 7:** Use of copper chloride as halogenating agent.

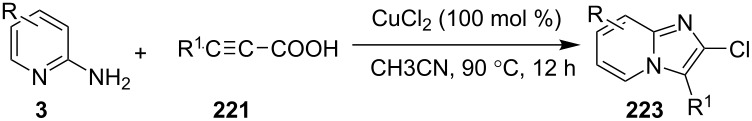							

Entry	R	R^1^	Yield (%)	Entry	R	R^1^	Yield (%)

1	H	C_6_H_5_	68	5	2-aminoquinoline	C_6_H_5_	72
2	3-F	C_6_H_5_	75	6	H	Et	50
3	4-Cl	C_6_H_5_	70	7	5-Cl	Et	55
4	5-Cl	C_6_H_5_	71	8	H	*n*-Pr	62

**Scheme 76:**
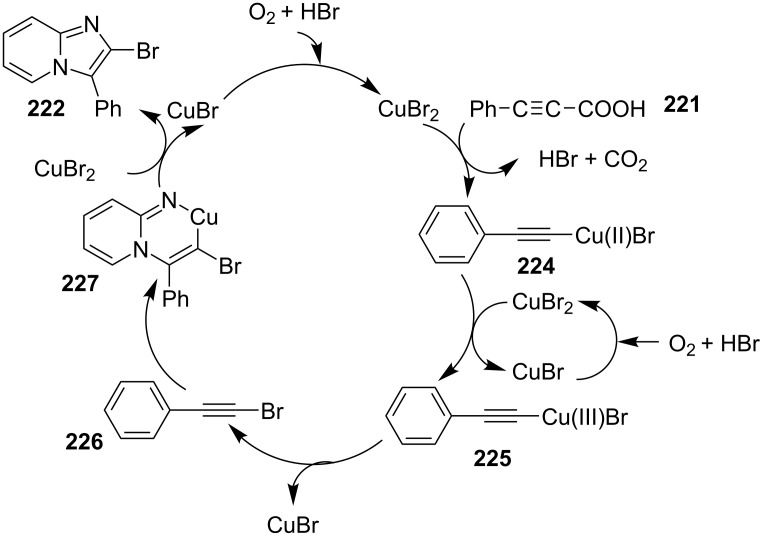
Mechanistic rationale for the synthesis of products.

Control experiments performed for the reaction displayed that alkynoic acid formed a Cu(II) intermediate **224** with CuBr_2_ followed by reductive elimination to generate an in situ bromoalkyne **226**. Diamination of this bromoalkyne with 2-AP in the presence of CuBr_2_ resulted in the final product (Scheme 76). These reaction conditions were also feasible for gram-scale synthesis.

**Scheme 77:**
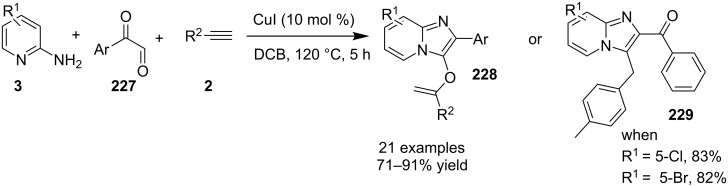
Copper-catalyzed synthesis of vinyloxy-IP.

**Scheme 78:**
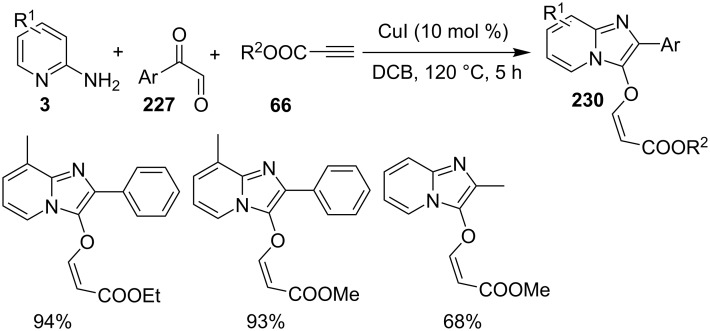
Regioselective product formation with propiolates.

**Scheme 79:**
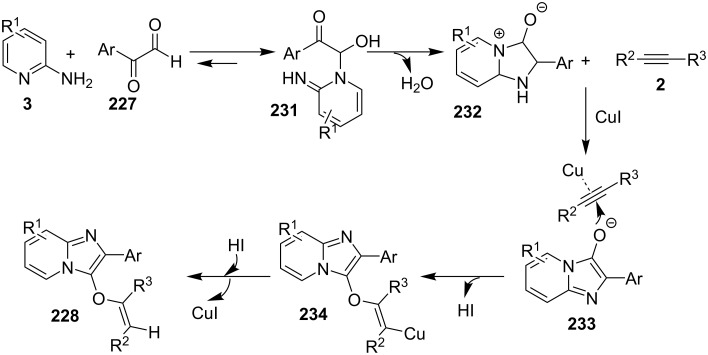
Proposed mechanism for vinyloxy-IP formation.

A novel regio- and stereoselective approach for the synthesis of vinyloxy-IP **228** catalyzed by CuI has been reported. The reaction has utilized 2-APs, 2-oxoaldehydes **227** and alkynes as reactive substrate (Scheme 77). This methodology was successful only in the presence of Cu(I) salt and no reaction occurred in the presence of Cu(II) salts [152]. The library of compounds designed by this methodology was quite interesting as aliphatic/aromatic/heteroaromatic alkynes substituted with both EW and EDGs were well tolerated under the optimized conditions. 2-AP moiety with EDGs (3-CH_3_/4-CH_3_) gave excellent yields whereas EWGs (5-Cl, 5-Br) resulted in 2-aroyl-IPs **229** than expected vinyloxy-IPs (Scheme 77). The reaction with propiolate **66** resulted in regioselective products due to the formation of a new C–O bond at the C-3 position of 1-alkyne (Scheme 78). Moreover, the use of internal alkynes like methyl 3-phenylpropiolate and dimethyl/diethyl acetylenedicarboxylate resulted in products with *Z*-selectivity. Use of radical scavenger BHT did not affect the product yield thereby showing no involvement of radical intermediate in the mechanism as depicted in Scheme 79.

**Scheme 80:**
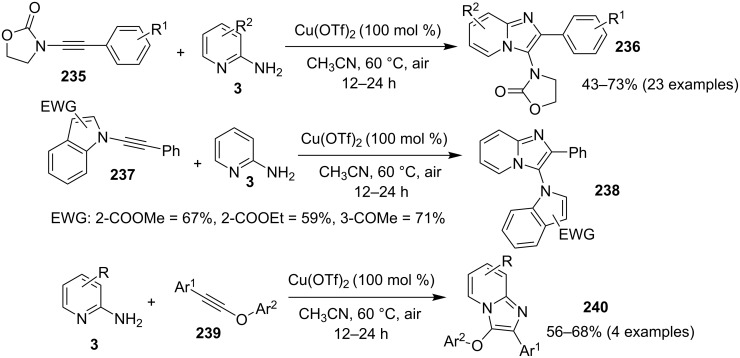
Regioselective synthesis of 3-hetero-substituted imidazo[1,2-*a*]pyridines with different reaction substrates.

The pyridone imine intermediate formed by the reaction between aminopyridine and oxoaldehyde undergoes dehydration to form imidazo[1,2-*a*]pyridinium-3-olates. Next, the alkyne activated by the copper(I) catalyst reacted with this zwitter ion, to form another reaction intermediate. Finally, the protonolysis of this intermediate with HI resulted in the regeneration of Cu(I) species and the final product. But in case of Cl- and Br- substituted 2-aminopyridines, the corresponding pyridone imine intermediate directly reacted with an alkyne to produce 2-aroylimidazopyridines via A^3^-coupling followed by 5-*exo-dig* cyclization instead of the formation of imidazo[1,2-*a*]pyridinium-3-olates due to the presence of electron-withdrawing substituents on the pyridine ring. Inspired from the work done by the groups of Gao et al., Zeng et al. and Garzón et al. [44,153–154] for the regioselective synthesis of IPs by using electronically highly biased internal alkynes, Dwivedi et al. have reported a facile and highly regioselective synthesis of 3-hetero-substituted imidazo[1,2-*a*]pyridines (Scheme 80) [155]. The synthesis involved the reaction between 2-APs and internal alkynes using copper triflate (Cu(OTf)_2_) as catalyst. The methodology involved an inverse regioselection for synthesizing IPs with amino functionality at C-3 position. Application of other copper salts did not give better results whereas no product was obtained with Cu(OAc)_2_. This synthesis was effective in air whereas the use of acid-base additives and other oxidants proved to be detrimental.

**Scheme 81:**
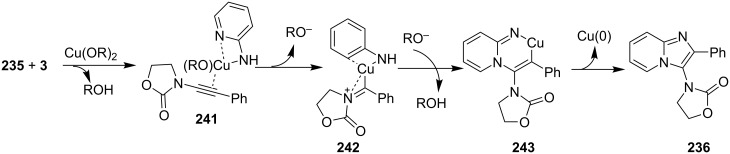
Mechanistic pathway.

The reaction was successful with both electron-rich as well as electron-deficient arylynamides with moderate and good results, respectively. The reaction conditions were so versatile that the use of indole-tethered alkynes **237** and oxygen counterparts **239** of these internal alkynes, that are, ynol ethers, were also found to be effective with differently substituted 2-APs (Scheme 80). Among diversified products synthesized by this methodology, 2-APs with EWGs have not been reported. Mechanistically, copper was expected to be involved in the formation of Cu-ketenimine complex assisted by *N*-ynamide nucleophilic displacement (Scheme 81). This was followed by the formation of a 6-membered cuprous metallocycle **243** which undergoes reductive elimination of Cu(0) and led to the formation of the final product.

**Scheme 82:**
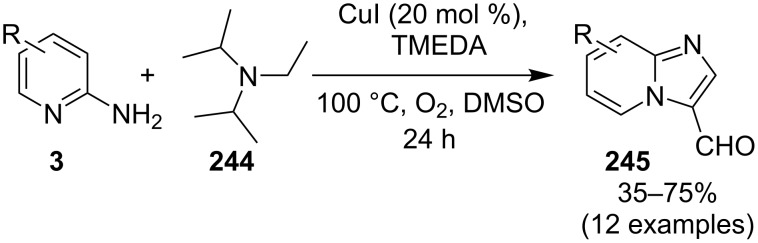
CuI-mediated synthesis of 3-formylimidazo[1,2-*a*]pyridines.

Rao et al. have described a Cu-catalyzed simultaneous C–C and C–N bond cleavage of tertiary ethylamines **244** with 2-AP to synthesize 3-formylimidazo[1,2-*a*]pyridines [156]. The reaction was carried out using CuI as a catalyst with tetramethylethane-1,2-diamine (TMEDA) as ligand under the atmosphere of oxygen (Scheme 82). Replacement of 2-AP with 2-aminobenzimidazoles under similar reaction conditions resulted in a successful synthesis of imidazo[1,2-*a*]pyrimidines. Ethyl containing tertiary (3°) amines were considered as most stable nitrogen containing compounds and were found to give better results than secondary (2°) amines, also the presence of an ethyl group was a prerequisite for this transformation. However, use of DIPEA as 3° amine found to increase the yield over Et_3_N.

**Scheme 83:**
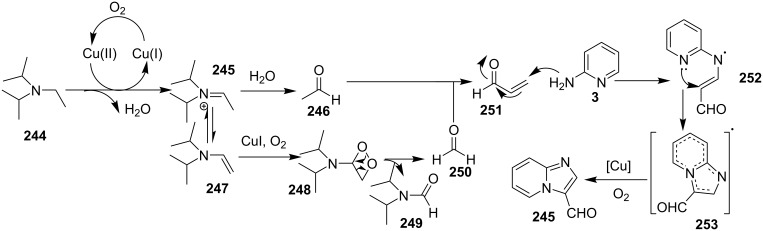
Radical pathway for 3-formylated IP synthesis.

The reaction was viable also with quinolin-2-amine and 3-aminopyridazine giving the product although in moderate and low yield, respectively. Inhibition of product formation on the application of TEMPO has revealed a radical pathway to be followed in the reaction (Scheme 83). Initially, 3° amine formed an immonium salt **245** that further gets converted to acetaldehyde (**246**) and formaldehyde (**250**), from the condensation of these two substrates formation of acrolein (**251**), took place. The acrolein thus formed reacted with 2-AP to form the target compound. This final conversion step operated through a radical pathway with the aid of a copper catalyst.

#### Palladium-catalyzed synthetic protocols

**Scheme 84:**
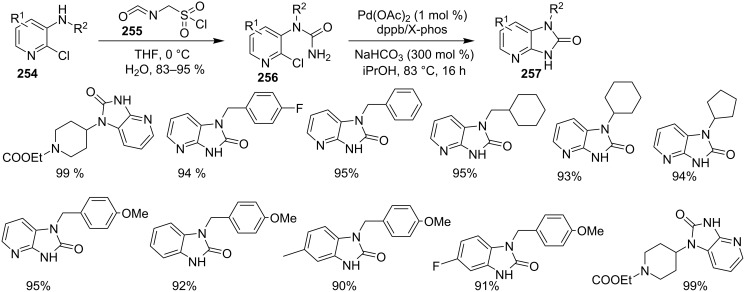
Pd-catalyzed urea-cyclization reaction for IPs.

A novel method for the efficient conversion of *o*-chloro-*N*-substituted pyridines to imidazo[4,5-*b*]pyridines was reported by the group of McLaughlin [54]. In this methodology first of all the ureas were synthesized by the reaction of *o*-chloroaryl/heteroarylamines **254** with *N*-chlorosulfonyl isocyanate **255** at 0 °C. The so formed ureas **256** undergo cyclization in the presence of Pd(OAc)_2_ as a catalyst, NaHCO_3_ as base and 1,4-bis(diphenylphosphino)butane (dppb) as a ligand for *o*-chloroaminopyridines (Scheme 84). However, with anilines such as less reactive *o*-chloroaniline more active X-Phos was used as a ligand to give an appreciable yield of the desired compound. This approach provided a rapid and efficient elaboration of the inexpensive starting materials into more complex heterocyclic structures with excellent yields.

**Scheme 85:**
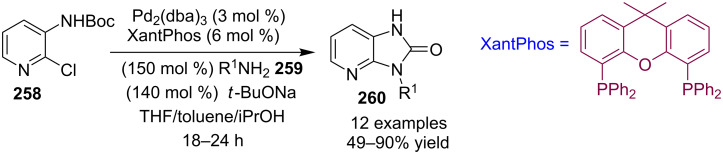
Pd-catalyzed one-pot-tandem amination and intramolecular amidation reaction.

A one-pot tandem amination and intramolecular amidative cyclization reaction for the synthesis of 3-arylated imidazopyridinones **260** was put forth by Scott from commercially available 3-amino-2-chloropyridine and anilines [53]. This methodology was quite similar to that reported previously by the group of McLaughlin, where a Pd(OAc)_2_-catalyzed cyclization of ureas was reported [54]. The amine group of 3-amino-2-chloropyridine was first protected (**258**) by carbamate group followed by TM-catalyzed amination of halide substituent with aniline **259**. This was the first report on transition-metal-catalyzed amination of an aryl halide having carbamate protected amine in the *ortho-*position. The reaction utilized Pd_2_(dba)_3_ as a precatalyst with XantPhos as most promising bidentate ligand for this Pd-catalyzed amination reaction. Refluxing tetrahydrofuran (THF) and toluene/iPrOH (4:1) found to be the best solvent systems (Scheme 85).

**Figure 5:**
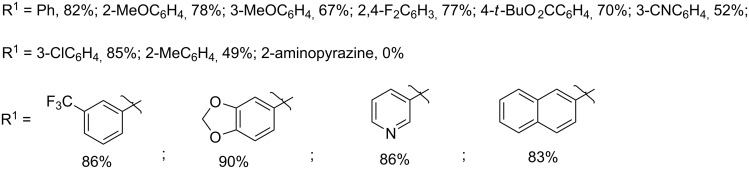
Scope of aniline nucleophiles.

Aniline nucleophile has tolerated a wide range of both EW and EDGs with *ortho*, *meta* and *para*-substitution along with heterocyclic amines, although in the case of 2-methylaniline was formed in 49% yield, a significant level of intermediate remained even after 24 hours. The use of heterocyclic amine viz., 3-aminopyridine was effective whereas, 2-aminopyrazine was unreactive (Figure 5).

**Scheme 86:**
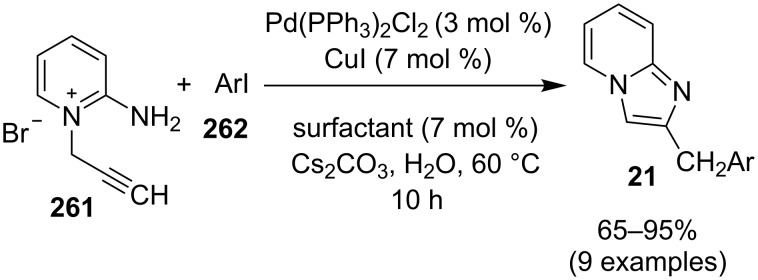
Pd–Cu-catalyzed Sonogashira coupling reaction.

Bakherad et al. have developed a Pd–Cu-catalyzed Sonogashira coupling reaction between 2-amino-1-(2-propynyl)pyrdinium bromide (**261**) with various aryl iodides **262** in the presence of a minimal amount of palladium catalytic system with copper iodide as co-catalyst using water as ecofriendly solvent (Scheme 86) [56]. The reaction was carried out under an inert atmosphere of argon.

To perform the reaction in aqueous medium sodium lauryl sulfate was used as a phase transfer reagent, in the absence of which a noticeable decrease in the yield was observed (entry 8, Table 8). Presence of EWGs on aryl iodide such as –NO_2_, –Cl and –CN found to be essential for good yield whereas iodobenzene and *p*-iodoanisole could not afford the desired compound.

**Table 8:** Effects of catalyst, co-catalyst, and surfactants on the heterocyclization reaction.

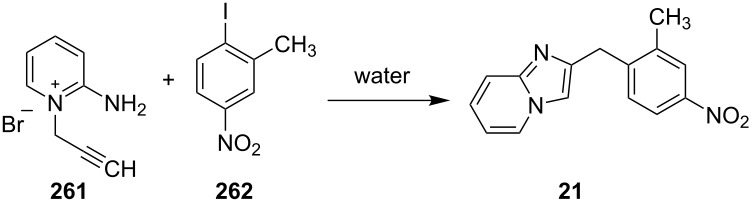				

Entry	Pd(PPh_3_)_2_Cl_2_	CuI	Sodium lauryl sulfate	Yield (%)

1	2 mol %	5 mol %	7 mol %	75
2	3 mol %	7 mol %	7 mol %	95
3	5 mol %	10 mol %	7 mol %	90
4	3 mol %	–	7 mol %	Nr
5	–	7 mol %	7 mol %	Nr
6	3 mol %	7 mol %	3 mol %	50
7	3 mol %	7 mol %	12 mol %	60
8	**3 mol %**	**7 mol %**	**–**	**10**

**Scheme 87:**
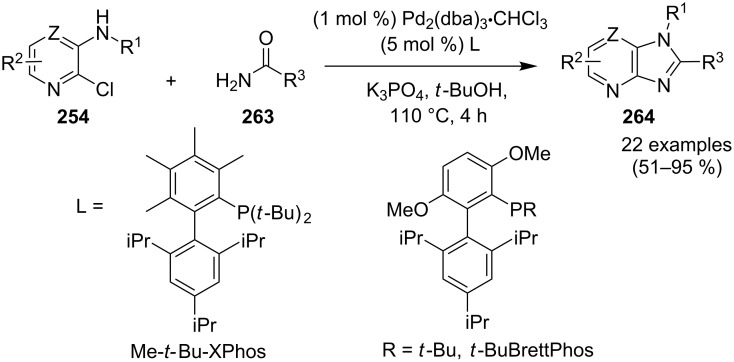
One-pot amide coupling reaction for the synthesis of imidazo[4,5-*b*]pyridines.

**Scheme 88:**
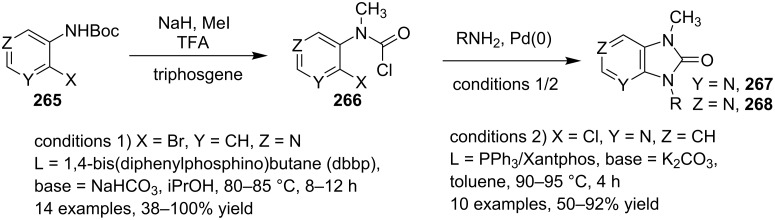
Urea cyclization reaction for the synthesis of two series of pyridines.

It is interesting to note that imidazo[4,5-*b*]pyridine derivatives behave as biological mimics for a number of benzimidazole scaffolds. This has increased the interest of scientific concern to develop a regioselective synthesis of its *N*-1-substituted derivatives. In this respect Rosenberg et al. have reported a facile synthesis of *N*-1-substituted imidazo[4,5-*b*]pyridines **264** using a palladium-catalyzed amide coupling reaction (Scheme 87) [157]. They have performed a one-pot reaction by coupling protected 2-chloro-3-aminopyridine **254** with the primary amide **263**, followed by in situ cyclization and dehydration to provide the final product with EDGs at N-1 position in appreciable yields. Lach and Koza have developed a one-pot tandem carbamoyl chloride amination followed by intramolecular urea cyclization to synthesize imidazo[4,5-*b*]pyridine-2-one **267** and imidazo[4,5-*c*]pyridine-2-one **268** [158]. The process was catalyzed by palladium in an inert atmosphere (Scheme 88). The methodology was very similar to that reported by the group of McLaughlin [54] with slight modification in product structure and conditions. Using this protocol two series of pyridines, for the synthesis of imidazo[4,5-*b*]pyridine-2-one were successfully synthesized. Xantphos was used as a ligand with Cs_2_CO_3_ as a base in 1,4-dioxane and for imidazo[4,5-*c*]pyridine-2-one, dppb was used as a ligand with NaHCO_3_ as a base in iPrOH as a solvent. Primary anilines used as one of the reactants delivered cyclized products in good yields except for *o*-substituted or moderately electron deficient aryl/heteroarylamines which resulted in a sluggish yield.

**Scheme 89:**
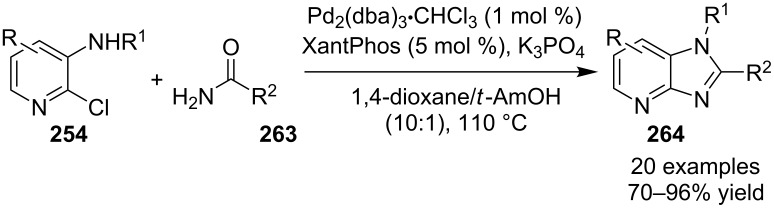
Amidation reaction for the synthesis of imidazo[4,5-*b*]pyridines.

The previous work of Rosenberg et al. has certain limitations like the high cost of the biarylphosphine ligand, the ineffectiveness of electron-deficient benzyl moiety and incompatibility of unhindered chlorides [157]. Inspired from this Rosenberg et al. have described a palladium-catalyzed amidation reaction using XantPhos as a ligand to synthesize imidazo[4,5-*b*]pyridines (Scheme 89) [159].

**Figure 6:**
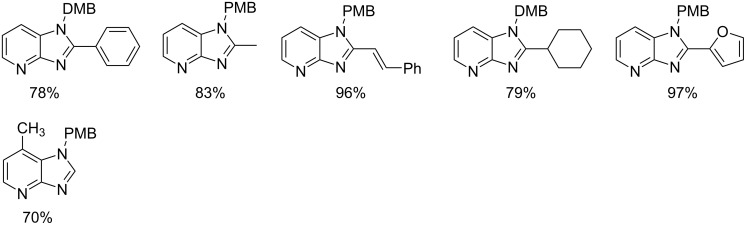
Amide scope.

The formation of the product faced competition with the pyridine byproduct **269** (Table 9). The use of protic solvents would minimize the byproduct formation but hindered the product formation in presence of bidentate ligand. In order to increase the yield of the desired product and to avoid the formation of the pyridine byproduct, a binary solvent system [(1,4-dioxane and t-amylalcohol (*t*-AmOH)] was used. The reaction enjoyed differently substituted *N*-aryl as well as *N*-alkyl substrates with product yields up to 96%. Only, *meta*-nitrobenzene gave a little lower yield of 57%. Moreover, the use of benzamide, acetamide, *trans*-cinnamide, cyclohexancarboxamide, 2-furanamide, and formamide as amide coupling partners also resulted in significantly higher product yields (Figure 6).

**Table 9:** Optimization of the solvent system.

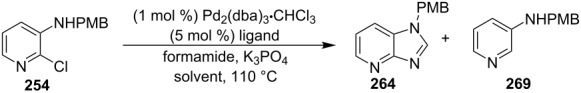			

Entry	Solvent (10:1)	Yield (%) **128a** (**128b**)	Conversion of **128a** (%)

1	1,4-dioxane/*t*-AmOH	93 (0)	100
2	1,4-dioxane/H_2_O	0 (74)	100
3	1,4-dioxane/MeOH	13 (18)	31
4	1,4-dioxane/iBuOH	30 (20)	84
5	1,4-dioxane/iPrOH	69 (0)	78

**Scheme 90:**
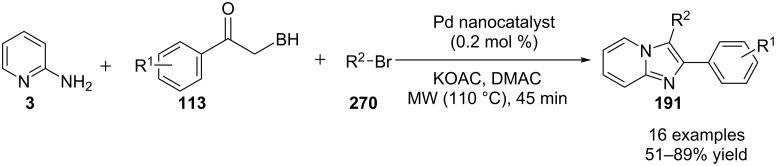
Pd NPs-catalyzed 3-component reaction for the synthesis of 2,3-diarylated IPs.

The group of Abarghooei and Mohebat have reported a three-component reaction catalyzed by Pd NPs for the synthesis of 2,3-diarylimidazo[1,2-*a*]pyridines **191** (Scheme 90) [160]. The reaction was performed under microwave irradiation thereby reducing the time from 18 h to 45 min analogous to conventional heating. The noteworthy feature of this work was reusability of Pd NPs supported on nano silica anchored with aminopropyl and 2-AP groups, thus reducing the problem associated with toxicity and high cost of this precious metal. The group has efficiently performed the reaction between 2-AP, 2-bromophenylethanone **113** and aryl bromide **270** using potassium acetate (KOAc) as the most effective base with dimethylacetamide (DMAc) as a solvent.

DMAc was the preferred choice over DMF because of its high stability, low toxicity, low corrosion, and good solubility. The added advantage of the procedure was minimal loading of the catalyst of just about 0.2 mol %. The reaction has accountable functional group tolerance; good yields were obtained with aryl bromides bearing EWGs than with EDGs along with high yields from bromobenzene, biphenyl bromide or bromopyridine (Table 10, entries 6, 10 and 11).

**Table 10:** Accountability of various substrates.

Entry	R^1^	R^2^	Yield (%)	Entry	R^1^	R^2^	Yield (%)

1	H	4-NO_2_C_6_H_4_	87	9	H	4-CH_2_CNC_6_H_4_	68
2	H	4-CNC_6_H_4_	89	10	H	4-C_6_H_5_C_6_H_4_	71
3	H	3-CNC_6_H_4_	84	11	H	Pyr	84
4	H	4-FC_6_H_4_	82	12	4-NO_2_	4-NO_2_C_6_H_4_	51
5	H	3,4-Cl_2_C_6_H_4_	78	13	4-NO_2_	4-ClC_6_H_4_	63
6	H	C_6_H_5_	77	14	4-CH_3_	4-NO_2_C_6_H_4_	69
7	H	4-CH_3_C_6_H_4_	66	15	4-CH_3_	4-CNC_6_H_4_	71
8	H	3-CH_3_C_6_H_4_	67	16	4-CH_3_	4-ClC_6_H_4_	68

**Scheme 91:**
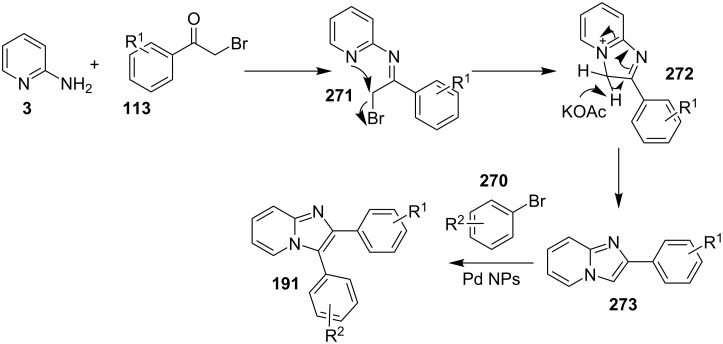
Plausible mechanistic pathway for Pd NPs-catalyzed MCR.

Bromoketones substituted with a nitro or a methyl group at the *para*-position were also found to give a good yield of the products. Mechanistically, the reaction was thought to proceed via formation of imine **132** by the reaction between 2-AP and α-bromoketone which was intramolecularly attacked by the nucleophilic nitrogen of the pyridine. The intermediate **133** thus formed was aromatized by KOAc to form the IP nucleus and final C3–H arylation of this moiety in the presence of Pd NPs resulted in the final compound (Scheme 91).

**Scheme 92:**
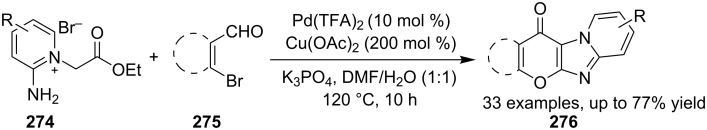
Synthesis of chromenoannulated imidazo[1,2-*a*]pyridines.

Annulated aromatic systems containing two or more heterocycles are widely present in natural and non-natural drug-like compounds. Very recently a palladium triflate [Pd(TFA)_2_]-catalyzed the synthesis of chromeno-annulated imidazo[1,2-*a*]pyridines **276** was reported by the group of K. Pandey in a one-pot fashion [161]. Previously this kind of synthesis was reported by many groups which suffered from certain bottlenecks like limited substrate scope, moderate yields and longer reaction times [162–164]. The reported one-pot strategy thought to be a multi-reaction system involving amidation, Knoevenagel condensation, Pd-catalyzed Wacker-type oxidation and C–O coupling (Scheme 92). The activity of tripotassium phosphate (K_3_PO_4_) as the base was found to be superior to bases like K_2_CO_3_/KOH/Cs_2_CO_3_/*t*-BuOK with a yield of 61% for the reaction between 2-amino-1-(2-ethoxy-2-oxoethyl)pyridi-1-ium bromide **274** and 2-bromobenzaldehyde **275**. The use of organic bases like DBU has decreased the yield to 15%. Among different Pd catalyst and oxidants optimized, a combination of Pd(TFA)_2_ and Cu(OAc)_2_ was found to give the best results under air atmosphere. In absence of Pd catalyst no product was formed whereas only 10% yield was obtained in the absence of copper oxidant (that was required for reoxidising Pd(0) to Pd(II) for completing the catalytic cycle).

**Scheme 93:**
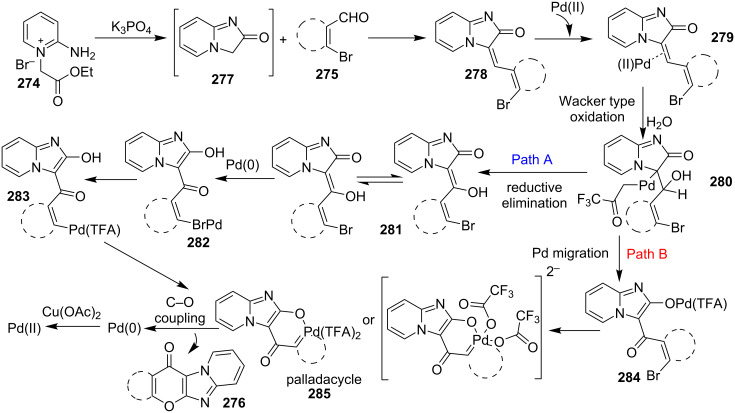
Mechanism for the synthesis of chromeno-annulated IPs.

Along with the catalytic system an 1:1 ratio of DMF and water as a solvent and air atmosphere were required for the reaction as use of anhyd. DMF or molecular sieves and inert atmosphere of nitrogen resulted in decreased yield. The reaction conditions tolerated differently substituted aromatic and heteroaromatic aldehydes except 1-bromo-3,4-dihydronaphthalene-2-carbaldehyde that gave a lower yield of 35%. On the other hand, C-5-substituted pyridinium salts gave lower yields as compared to unsubstituted or C-3 substituted salts. C-5-substituted pyridinium salts resulted in the formation of *N*-acetyl-2-aminopyridines as a byproduct along with the final compound. Moreover, 5-nitro-substituted pyridinium salt did not give the desired product under optimized conditions. The reaction mechanism involved an initial base-mediated intramolecular amidation and Knoevenagel condensation of two reactants to form an intermediate that coordinated to Pd(II) **279**. This complex on reaction with water via Wacker-type oxidation gave intermediate **280** that could form palladacylce **285** via two possible pathways. Finally, reductive elimination of this palladacycle complex led to the formation of the C–O bond and gave the desired cyclized product **276** (Scheme 93).

#### Zinc-catalyzed synthetic protocols

**Scheme 94:**
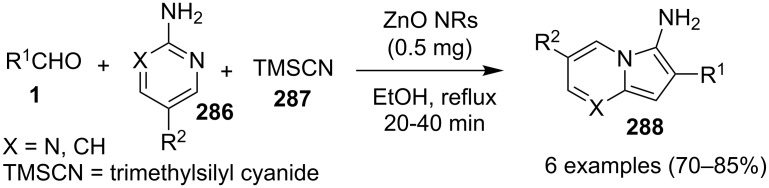
Zinc oxide NRs-catalyzed synthesis of imidazo[1,2-*a*]azines/diazines.

Zinc oxide nanorods (ZnO NRs) have been used successfully by Sadjadi and Eskandari in synthesizing imidazo[1,2-*a*]azines and diazines via a three-component one-pot reaction [165]. They have employed a novel, solvent-free procedure for synthesizing NRs, and used them as a heterogeneous catalytic system with a minimum loading of 0.5 mg. They have employed benzaldehydes, 2-aminoazines **286** and trimethylsilyl cyanide (TMSCN, **287**) as reacting substrates (Scheme 94).

These NRs found to posses more activity than bulk ZnO. Table 11 represents six differently substituted azines and diazines synthesized by the reported procedure. The results clearly indicated that these reaction conditions were compatible with both electron donating (ED) as well as electron withdrawing groups (EWGs) on reacting compounds.

**Table 11:** Effect of substitutions in the synthesis of azines/diazines.

Entry	R^1^	R^2^	X	Yield (%)

1	4-NO_2_-C_6_H_4_	H	N	81
2	4-OMe-C_6_H_4_	H	N	70
3	C_6_H_5_	Br	CH	82
4	3-NO_2_-C_6_H_4_	Me	CH	85
5	4-Cl-C_6_H_4_	Me	CH	85
6	4-Me-C_6_H_4_	Me	CH	85

**Scheme 95:**
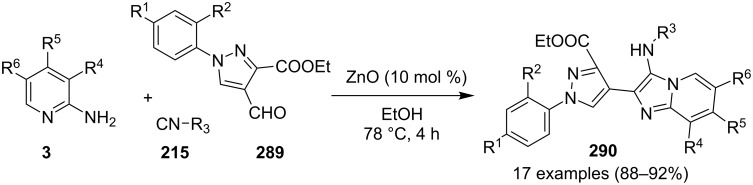
Zinc oxide-catalyzed isocyanide based GBB reaction.

Owing to the medicinal importance of imidazo[1,2-*a*]pyridines and pyrazoles, the group of Swami have coupled these entities by isocyanide based GBB 3-component reaction (Scheme 95) [166]. This type of condensation approach was initially catalyzed by a number of acid catalysts viz., ZnCl_2_, MgCl_2_, ZrCl_2_, Sc(OTf)_3_, AcOH, *p*-TSA, etc. [18,167–171]. Most of these methods have certain limitations like low product yield, toxicity, longer reaction time and absence of reusability of the catalyst. The use of ZnO NPs provided a greener and atom economic approach for the synthesis of pyrazole-coupled imidazo[1,2-*a*]pyridines **290**. The group has chosen alkyl-4-formyl-1-substituted phenyl-1*H*-pyrazole-3-carboxylates **289**, 2-aminopyridines **3** and isocyanides **215** as reactant for the syntheses of IP and pyrazole fused product.

**Scheme 96:**
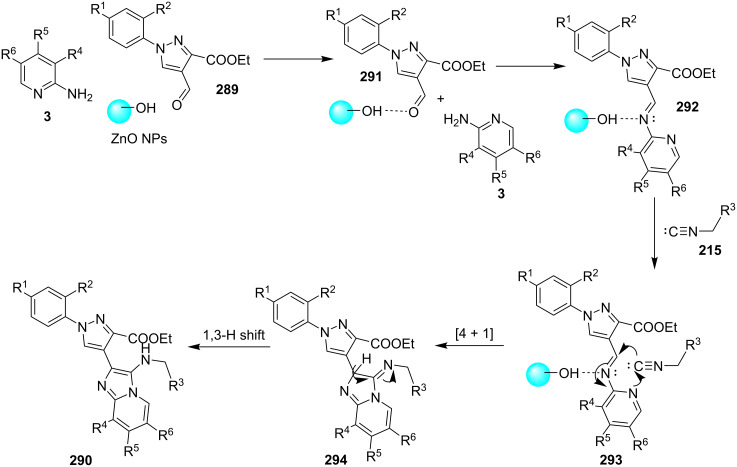
Reaction pathway for ZnO-catalyzed GBB reaction.

**Scheme 97:**
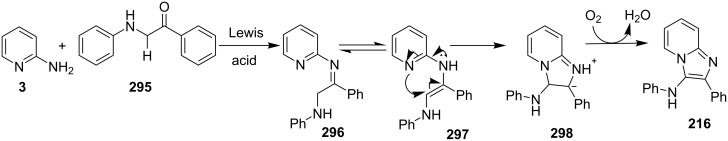
Mechanistic pathway.

The greener aspect of the procedure lied in the recyclability and reusability of the used catalyst. The synthetic protocol enjoyed a rich library of synthesized compounds with the negligible effect of directing groups on yield of the final compounds. The beauty of the process lied in wide variation among all the three reacting substrates. The proposed mechanism of the reaction involved initial formation of imine **292**, which was further activated by ZnO NPs by increasing the electrophilicity of imine carbon. Which was further attacked by isocyanide, following [4 + 1] cycloaddition to give cyclic adduct **294**. The so formed adduct finally underwent 1,3-H shift to furnish the target compound **290** (Scheme 96). Thus, ZnO played the role of a reusable and recyclable acid catalyst by initiating the formation of imine and enhancing the elecrophillic nature of imine carbon. Further exploration of zinc to act as a catalyst for this type of synthesis was made by Haan et al*.* who have applied zinc iodide (ZnI_2_) as a Lewis acid catalyst for the synthesis of 3-aminoimidazo[1,2-*a*]pyridines **216** [24]. The synthesis involved a two component one-pot reaction between 2-APs and α-amino carbonyl compounds under milder conditions (Table 12). In order to increase the yield of the desired product molecular sieves have been used to remove the water generated during the reaction that might have affected the reaction outcome. This protocol resulted in an appreciable yield of the product generated with differently substituted α-aminocarbonyl compounds **295** (Table 12). However in the case of 2-APs **3** groups like methyl were well tolerated at different positions viz., *ortho*/*meta*/*para*, also 74% yield with *para*-methoxy-2-AP was quite appreciable. However, 3-benzyloxy and 4-chloro-substituted 2-AP gave lesser yields of about 52 and 46%, respectively, the reaction did not result in the formation of the product with 4-Br substituent (entry 8, Table 13). The reaction was expected to proceed via in situ formation of imine **296** catalyzed by Lewis acid, followed by cyclization, H transfer and oxidation which led to the formation of the final product **216** (Scheme 97). The reaction was also applicable for the synthesis of many pharmaceutically relevant imidazopyridines (IPs).

**Table 12:** Formation of 3-aminoimidazo[1,2-*a*]pyridines from α-aminocarbonyl compounds.

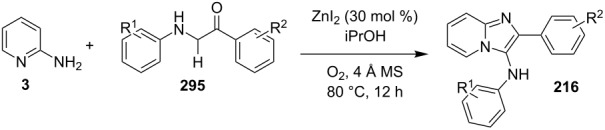							

Entry	R^1^	R^2^	Yield (%)	Entry	R^1^	R^2^	Yield (%)

1	H	H	79	6	4-F	H	74
2	4-CH_3_	H	89	7	H	4-Cl	82
3	3,4-(CH_3_)_2_	H	85	8	H	4-Br	77
4	H	4-CH_3_	92	9	4-Br	H	72
5	*t*-Bu	H	70	10	4-OMe	H	79

**Table 13:** Synthetic approach with differently substituted 2-APs.

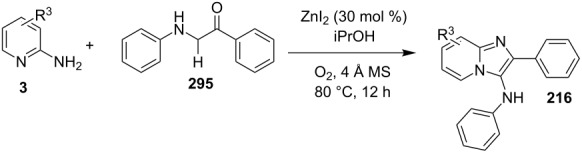					

Entry	R^3^	Yield (%)	Entry	R^3^	Yield (%)

1	3-Me	80	5	4-OMe	74
2	4-Me	70	6	3-OBn	52
3	5-Me	78	7	4-Cl	46
4	6-Me	62	8	4-Br	NR

**Scheme 98:**
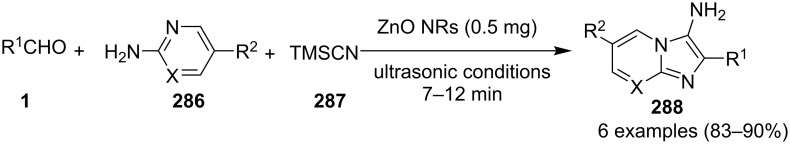
ZnO NRs-catalyzed MCR for the synthesis of imidazo[1,2-*a*]azines.

Sadjadi and Eskandari, in continuation of their own work reported in 2012 [167], have reported a similar three-component one-pot reaction of benzaldehydes **1**, 2-aminoazines **286** and trimethylsilyl cyanides **287** for the synthesis of imidazo[1,2-*a*]azines [172]. The reaction was catalyzed by ZnO NRs as they have used in their previous work. The difference was the use of ultrasonic conditions in the present protocol thus, following the principles of green chemistry (Scheme 98). Benzaldehydes and azines substituted with both EW as well as EDGs gave an excellent yield of the targeted compounds in a range of 83–95%.

#### Iron-catalyzed synthetic protocols

**Scheme 99:**
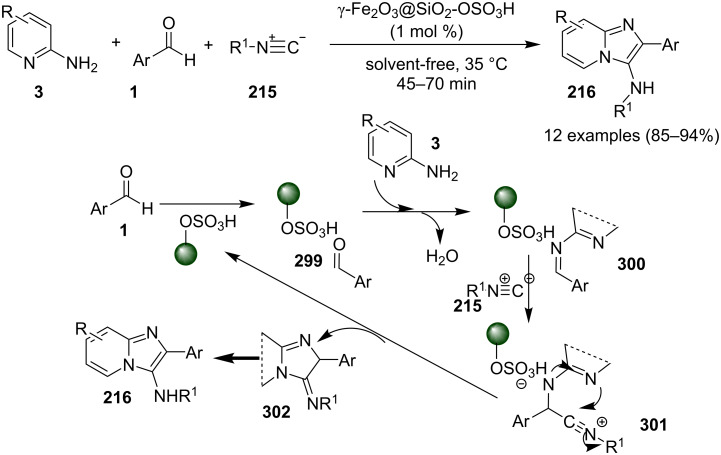
Ugi type GBB three-component reaction.

Rostamnia et al. in 2012 have developed a green and rapid synthesis of 3-aminoimidazopyridine skeletons via the Ugi like GBB three-component reaction (Scheme 99) [173]. This skeleton exhibits a wide spectrum of biological activities. They have used superparamagnetic NPs on modified sulfuric acid (γ-Fe_2_O_3_@SiO_2_-OSO_3_H) as a simple, economical, easily recoverable and reusable catalyst. The added advantage was the feasibility of the reaction with just 1 mol % of the catalyst under solvent and additive-free conditions at 35 °C. The catalyst was reusable up to 5 synthetic cycles with negligible effect on the product yield after every cycle. The reaction has tolerated both *ortho*/*meta-*methyl-substituted 2-APs and variedly substituted benzaldehydes with aliphatic isocyanides.

**Scheme 100:**
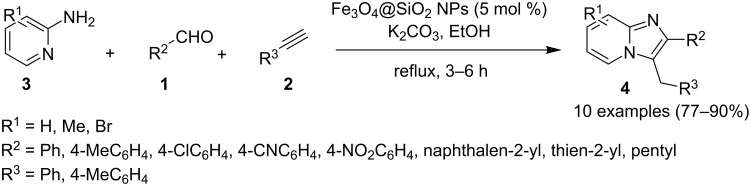
Magnetic NPs-catalyzed synthesis of imidazo[1,2-*a*]pyridines.

Owing to the easy recovery of magnetic NPs from the reaction system, Maleki has reported silica-supported iron oxide (Fe_3_O_4_@SiO_2_) NPs as efficient catalysts for the synthesis of imidazo[1,2-*a*]pyridines in good to excellent yields [174]. Substituted 2-APs **3**, aldehydes **1** and terminal alkynes **2** were used as substrates under refluxing conditions (Scheme 100). The catalyst used was easily recovered at the end of the reaction and was separated by magnetic filtration, washed with water and air dried to make it ready for subsequent reactions. This MCR was characterized by high product purity and a good product library.

**Scheme 101:**
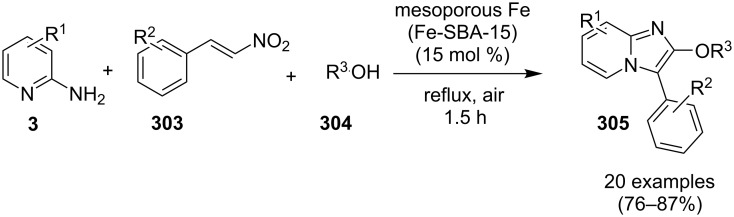
Regioselective synthesis of 2-alkoxyimidazo[1,2-*a*]pyridines catalyzed by Fe-SBA-15.

The regioselective synthesis of novel 2-alkoxyimidazopyridine derivatives **305** via an one-pot three-component reaction of 2-AP, nitrostyrene **303** and alcohol **304** using Fe-SBA-15 as a catalyst was reported by Payra et al. (Scheme 101) [175]. Various iron catalysts viz., FeCl_3_, FeSO_4,_ and nano-Fe_3_O_4_ have been employed for this reaction but they resulted in a poor yield of about 37, 19 and 41%, respectively. Metals other than iron supported on SiO_2_ were also tried among which Cu-SiO_2_ resulted in 44% of the product whereas with Ru and Pd-SiO_2_ the reaction was not feasible. Moreover, a reaction with the active catalyst was not possible at room temperature even after 12 h of reaction time whereas at refluxing temperature a completion of the reaction takes place in 1.5 h reaction time.

**Scheme 102:**
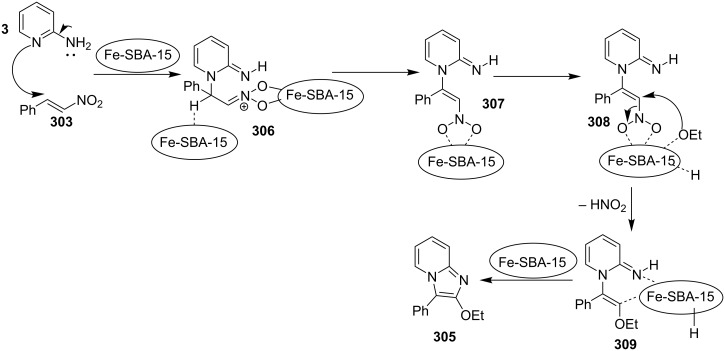
Plausible mechanistic pathway for the synthesis of 2-alkoxyimidazopyridine.

2-AP substituted with a methyl group, nitrostyrene substituted with both ED and EWGs, primary alcohols such as ethanol, *n*-propanol, and *n*-butanol were found to be effective substrates in generating a library of 20 compounds. However, secondary and tertiary alcohols did not participate in this protocol. The use of Fe-SBA-15 has increased the yield to an appreciable extent. Fe^3+^ sites of the catalyst played a vital role by accelerating the aza-Michael addition of nitrostyrene to the endocyclic nitrogen of aminopyridine. This was followed by substitution of the NO_2_ group by an ethoxy group that resulted in the formation of the final product **305** by oxidative addition of intermediate **152** (Scheme 102).

**Scheme 103:**
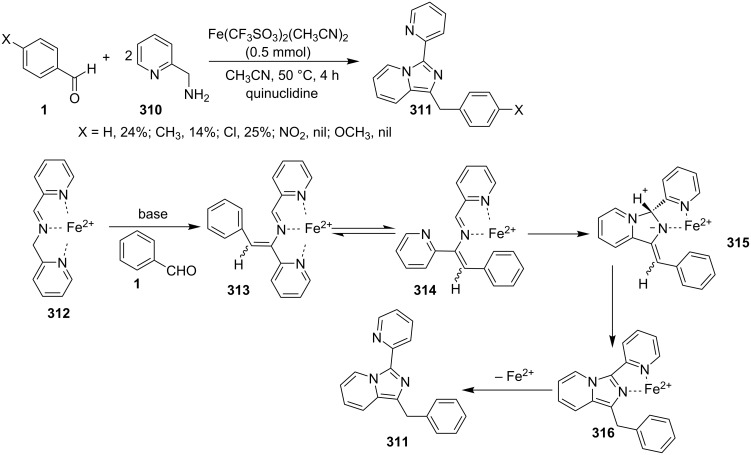
Iron-catalyzed synthetic approach.

Albano et al. have reported an unexpected synthesis of novel imidazo[1,5-*a*]pyridine derivatives **311** in the reaction between benzaldehyde and 2-picolylamine **310** [176]. The synthesis has been achieved through the action of Fe^2+^ ion on a dynamic library of imines generated in situ via condensation of benzaldehydes and 2-picolylamines (in 1:2 molar ratio). Evaluation of electronic effects has shown that aldehydes with moderate EW and EDGs gave good yields whereas *p*-OCH_3_- and *p*-NO_2_-substituted benzaldehydes did not give the desired compounds (Scheme 103). Fe(CF_3_SO_3_)_2_(CH_3_CN)_2_ has been used as the source of Fe^2+^ ions in this reaction. The suggested mechanism clearly defined the role of Fe^2+^ ions in carrying forward the reaction as depicted in Scheme 103.

**Scheme 104:**
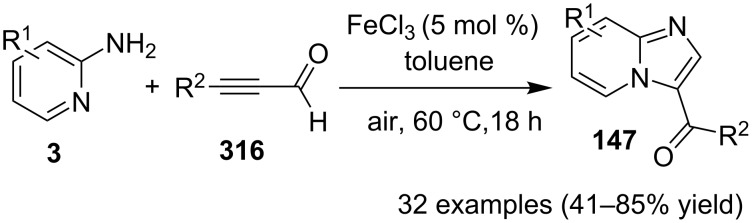
Iron-catalyzed aminooxygenation reaction.

Chen et al. have demonstrated an efficient iron-catalyzed intermolecular aminooxygenation reaction to synthesize 3-aroylimidazo[1,2-*a*]pyridines **147** [177]. The reaction was successful with ferric chloride at 60 °C for a time period of 18 h (Scheme 104). The role of oxygen was indispensable for this procedure as an only a trace amount of product was obtained under argon atmosphere. The variety of 2-APs with both EW and EDGs like aryl, alkyl, nitro, halides, etc. was well tolerated.

**Scheme 105:**
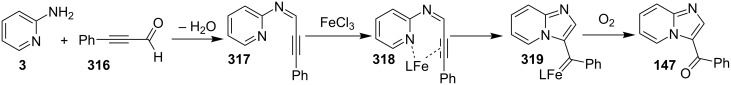
Mechanistic pathway.

The steric effect was also negligible as bulky *tert*-butyl, alkyl naphthalenes and heteroaromatic substituted ynals **316** also gave the good results. Application of TEMPO and BHT did not affect the reaction thus depicting it to be an ionic pathway. Mechanistically, imine intermediate **317** was formed by the reaction of aldehyde and amine. This imine formed a complex with an iron catalyst, which was then cyclized to form carbine **319** that on oxidation resulted in the desired product (Scheme 105).

#### Rhodium-catalyzed synthetic protocols

**Scheme 106:**
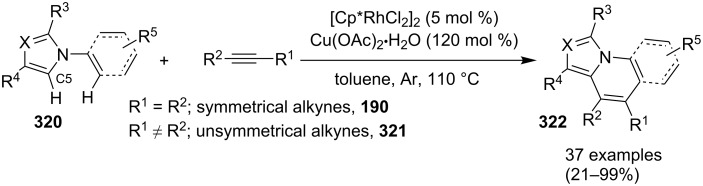
Rh(III)-catalyzed double C–H activation of 2-substituted imidazoles and alkynes.

**Scheme 107:**
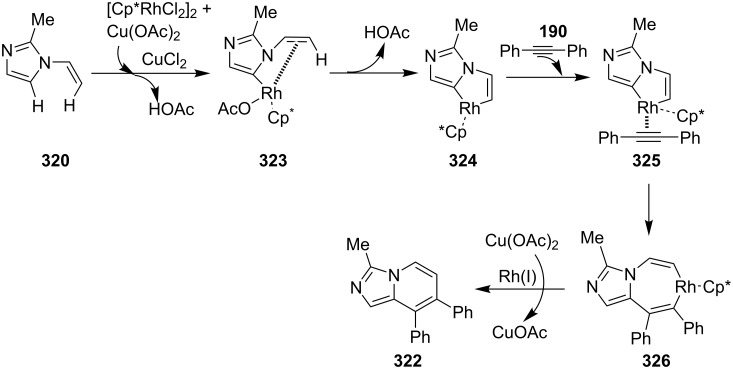
Plausible reaction mechanism.

Inspired by the work of Shi and co-workers for oxidative cycloaromatization of biaryls with alkynes without directing groups [178], Huang et al. have reported a direct, double C–H activation of vinylic C(sp^2^)–H and C(5)–H of 2-substituted imidazole **320** by a Rh(III)-catalytic system, followed by its coupling with alkynes. Also, Rh-catalyzed direct C–H activation of nitrogen heterocycles without directing groups was a huge challenge which was successfully overcome by this group. They have used [Cp^*^RhCl_2_]_2_ as a catalyst and Cu(OAc)_2_ as an oxidant (Scheme 106) [27]. The reaction was not compatible with other TMs such as [RuCl_2_(*p*-cymene)]_2_, (PPh_3_)_3_-RhCl and Pd(OAc)_2_ which showed much less or negative catalytic activity. This system has well tolerated symmetrical and unsymmetrical alkynes, rather than terminal alkynes along with a differently substituted imidazole moiety. Moreover, *N*-phenylimidazole bearing either ED or EWGs at the *para*-position of the *N*-aryl ring showed good reactivity. However, *ortho*-substitution gave only a 25% yield, probably because of steric hindrance. Imidazoles with *N*-heteroaryl group were also compatible under the reported conditions, thus producing a large variety of compounds. In this reaction, Rh(III) undergo insertion at C(5a)–H, presumably facilitated by the assistance of adjacent π-system that was followed by vinylic C(sp^2^)–H activation to afford the five-membered rhodacycle intermediate **325**. Subsequently, alkyne coordinates to the Rh(III) complex with subsequent reductive elimination to yield the final product (Scheme 107).

A non-aromatic C(sp^2^)–H bond activation-functionalization of imidazolium salts **327** with internal alkynes catalyzed by Rh(III) was reported by Thenarukandiyil et al*.* to synthesize imidazo[1,2-*a*]pyridinium salts **328** [179]. The same group has initially reported reactivity of N-heterocyclic carbenes (NHCs) as directing group for aromatic/heteroaromatic C−H activation with alkynes to construct positively charged π-conjugated organic materials. However, non-aromatic C(sp^2^)–H bond activation suffered from few challenges like stabilization of the generated positive charge by delocalization and hinderance in the activation of the non-aromatic C(sp^2^)–H bond due to the presence of substituents on the double bond, thus they have envisioned extending the strategy to non-aromatic C(sp^2^)−H bonds as well.

**Scheme 108:**
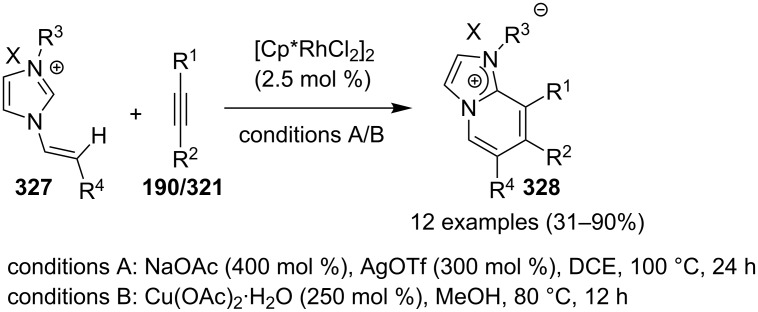
Rh(III)-catalyzed non-aromatic C(sp^2^)–H bond activation–functionalization for the synthesis of imidazo[1,2-*a*]pyridinium salts.

However, the design and development of novel synthetic pathways through the exploration of non-aromatic C(sp^2^)−H containing organic substrates are of greater demand. Therefore, in the Rh(III)-catalyzed non-aromantic C(sp^2^)–H activation functionalization reaction, the role of NHC of N-heterocyclic motifs as a directing group along with the crucial involvement of copper/silver salts in this transformation has been envisaged (Scheme 108) [179]. Moreover, the annulation was substantially feasible with various substituents such as methyl, butyl, and benzyl in the imidazolium substrates in good yields. In case of alkynes, diaryl acetylenes bearing EW and EDGs and aryl–alkyl substituents gave a good yield. Diarylacetylenes bearing either EWG (NO_2_) or EDG (OMe) on one of the phenyl rings produced regioisomeric products in 74% and 87% overall yields, respectively.

**Scheme 109:**
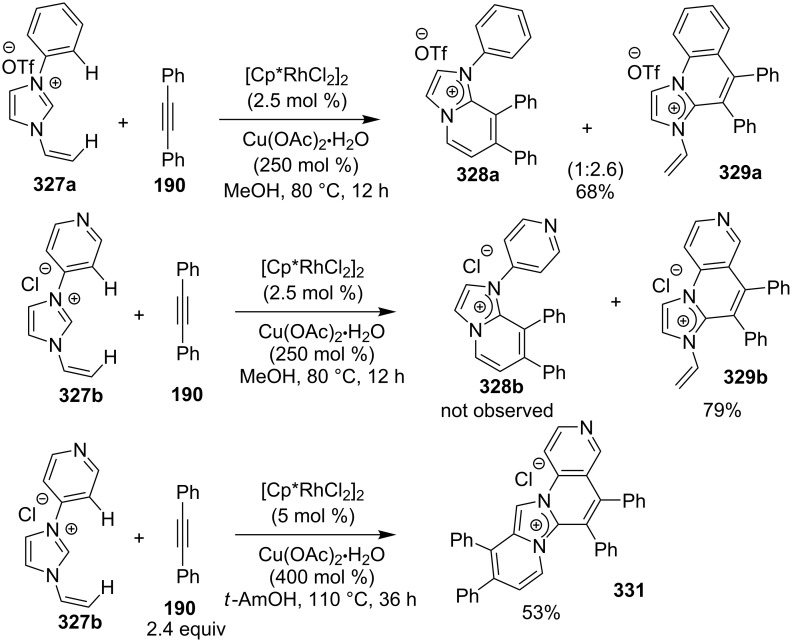
Reactivity and selectivity of different substrates.

Interestingly an imidazolium salt with an iodine anion gave only 31% of the product with dipropylacetylene whereas the salt of the triflate anion gave 86% yield with a similar alkyne. In this process, C–H activation led to the formation of a five-membered cyclometalated complex by the in-built NHC ligand coordination. The alkyne then coordinated to the so formed complex followed by reductive elimination leading to the final product in appreciable yields. This protocol provided a comparison between the reactivity of non-aromatic vs aromatic C(sp^2^)–H bond within a single molecule. The results have shown higher reactivity of the aryl C(sp^2^)–H bond (Scheme 109, **329a**). Moreover, the use of pyridine substituent has resulted in pyridyl C(sp^2^)–H functionalized moiety as the only product, interestingly excess of alkyne at higher temperature provided a double C(sp^2^)–H activated product (Scheme 109, **329b** and **331**).

**Scheme 110:**
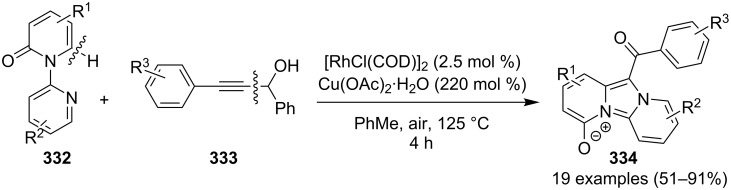
Rh-catalyzed direct C–H alkynylation by Li et al.

Inspired by the limitations associated with the use of terminal alkynes in C–H alkynylation like its polymerization, the use of alkynes with bulky silyl groups etc., Li et al. have designed a Rh-catalyzed direct C–H alkynylation of 2-pyridones **332** utilizing propargyl alcohols **333** as the coupling partner to synthesize 11-acylated-imidazo[1,2-*a*:3,4-*d*]dipyridin-5-ium-4-olate **334** (Scheme 110) [180].

**Scheme 111:**
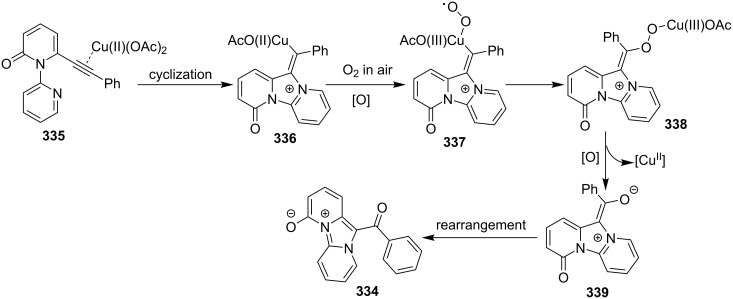
Suggested radical mechanism.

Propargyl alcohols containing ED/EWGs such as -OMe, -Et, -Cl and -F at the *para* as well as *meta*-position were well tolerated as compared to the *ortho*-position due to steric hindrance. However, the use of aliphatic alkynes was not compatible with this transformation and gave only a trace amount of the product. Moreover, the groups like -Cl, -CF_3_, -F or -OBn were well tolerated at C-3 and C-4 positions of the 2-pyridone ring whereas no product was obtained with 5-Me substitution. The use of TEMPO resulted in traces of the final compound suggesting a radical mechanism for the reaction (Scheme 111).

#### Ruthenium/lanthanum/scandium-catalyzed and miscellaneous synthetic protocols

**Scheme 112:**
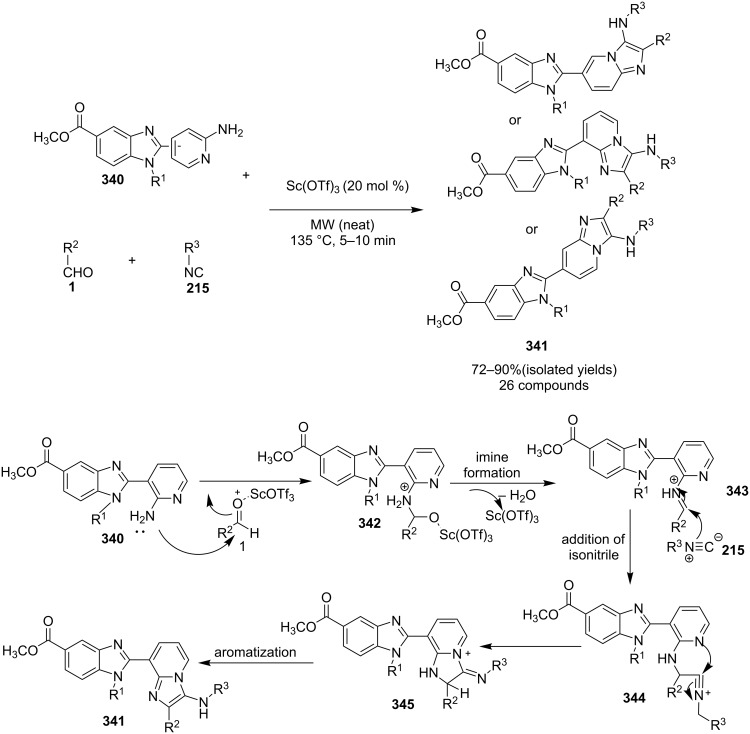
Scandium(III)triflate-catalyzed one-pot reaction and its mechanism for the synthesis of benzimidazolylimidazo[1,2-*a*]pyridines.

Owing to the catalytic applications of scandium, Maiti et al. have reported a new synthetic methodology for rapid synthesis of the hybrid molecule by integration of two different pharmacophores. This domino protocol involved GBB reaction as a key transformation in the reaction of substituted benzimidazole-linked aminopyridine **340**, aldehydes and isonitriles [181]. This reaction was catalyzed by scandium(III) triflate under solvent-free microwave irradiation and afforded a library of benzimidazolylimidazo[1,2-*a*]pyridines **341** in excellent yields in a one-pot mechanism (Scheme 112). Scheme 112 also depicts a probable scandium(III) triflate-driven mechanism. In this regard, the microwave-assisted, solvent-free strategy was proven as the environmentally benign, powerful approach for the efficient and quick formation of the desired products. However, protic acids (TFA and *p*-TSA) and the Lewis acid ZnCl_2_ as catalysts failed to deliver the desired product in significant yield. Moreover, the absence of scandium(III) triflate did not generate the required compound under the reported conditions. Further exploration of substituents has shown that aldehydes containing EWGs tend to react much faster than EDGs and neutral aldehydes. Also, the aliphatic aldehydes were nonresponsive under theses conditions. The probable reason for their inertness might be the facile imine formation of the electron-deficient aldehydes with benzimidazole-linked aminopyridine under Sc(OTf)_3_ catalysis and faster addition of isocyanides onto the in situ formed iminium ion **343** possessing electron-rich substrates.

**Scheme 113:**
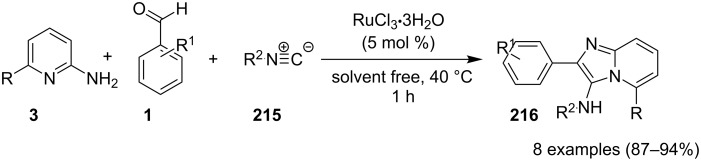
RuCl_3_-assisted Ugi-type Groebke–Blackburn condensation reaction.

Rostamnia and Hassankhani have reported a RuCl_3_-catalyzed Ugi-type Groebke–Blackburn condensation reaction of compounds **1** and **3** with **215** under neat reaction conditions [182]. The reaction was successfully carried out in the absence of ligand, solvent, and additive (Scheme 113).

**Scheme 114:**
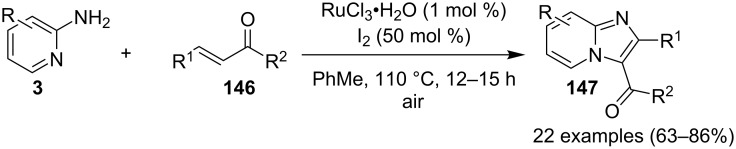
C-3 aroylation via Ru-catalyzed two-component reaction.

Furthermore, the reaction was carried out at 40 °C over a period of 1 h and resulted in the desired products in good yield (87–94%). ED and EWGs on both 2-AP and aldehydes well suited to the reaction also the use of cyclohexyl and *tert*-butyl isocyanide did well in this synthesis. Although being a homogeneous catalyst, commercial availability of RuCl_3_·3H_2_O as compared to other Ru sources made this reaction more compatible among another similar kind of Ru-catalyzed reactions. Reduction in the costs of ligands, solvents and other additives made this protocol more economical. Ramya et al. have reported an efficient and facile synthetic strategy for **147** from easily available starting materials viz., 2-APs **3** and substituted chalcones **146** (Scheme 114) [183]. The reaction was catalyzed by RuCl_3_ and co-catalyzed by molecular iodine.

**Scheme 115:**
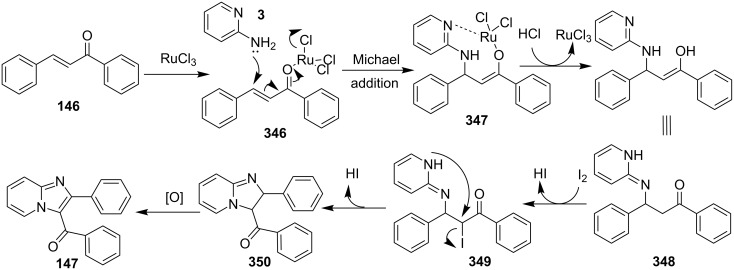
Regioselective synthetic mechanism.

**Scheme 116:**
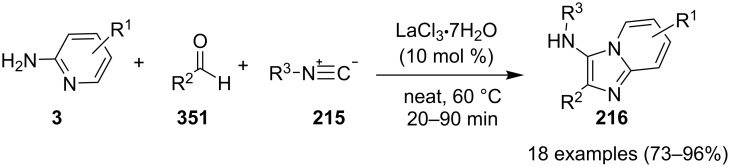
La(III)-catalyzed one-pot GBB reaction.

Iodine was mandatory in this protocol as its absence resulted in a poor yield of the product, whereas 50 mol % loading resulted in an excellent yield of the product (maximum of 86%). The reaction was not compatible with other catalysts like Ag(OAc)_2_, Bi(OTf)_3_ and Yb(OTf)_3_ as they led to a maximum yield of only 20% and in case of Ag_2_CO_3_ and Zn(OTf)_2_ no product was formed. Absence of catalyst or co-catalyst, in this case, proved to be futile. This one-pot tandem oxidative cyclization procedure has well tolerated a wide variety of substituted chalcones/heteroaryl chalcones and 2-APs. The steric effect was found to be ineffective for this transformation as 3-methyl and 6-methyl substituted 2-AP gave a good yield. The beauty of this process lies in very little loading of the catalyst and co-catalyst. The reaction conditions were also compatible with gram-scale production of the target compounds. The reaction was supposed to proceed via Michael addition because when the reaction was performed under nitrogen atmosphere only Michael adduct **348** was obtained. This adduct when subjected to optimized conditions, i.e., under air it undergoes intramolecular cyclization and oxidation to yield the final moiety **147** (Scheme 115). In this mechanism, the Ru catalyst helped in activation of chalcone and iodine promoted the intramolecular cyclization step. The mechanism has proved air atmosphere to be mandatory for this conversion because even on retaining the catalytic and solvent conditions N_2_ atmosphere failed to produce the targeted moiety. In order to exploit the catalytic functionality of lanthanum Shinde et al. have reported a one-pot GBB reaction for imidazo[1,2-*a*]pyridines **216** by an unprecedented solvent-free, hydrated lanthanum chloride-catalyzed synthetic protocol [184]. The methodology enjoyed wider applicability with differently substituted aromatic/heteroaromatic, aliphatic, as well as metal-containing aldehydes **351** (ferrocene-2-carboxaldehyde) along with various 2-APs **3** and aromatic/non-aromatic/aliphatic isocyanides **215** (Scheme 116).

**Scheme 117:**
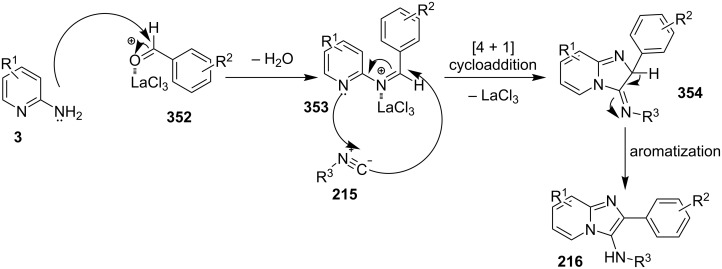
Mechanistic approach for the synthesis of imidazo[1,2-*a*]pyridines.

**Scheme 118:**
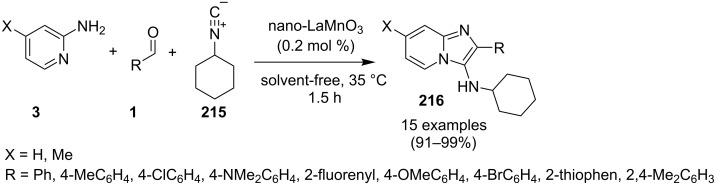
Synthesis of imidazo[1,2-*a*]pyridine using LaMnO_3_ NPs under neat conditions.

**Scheme 119:**
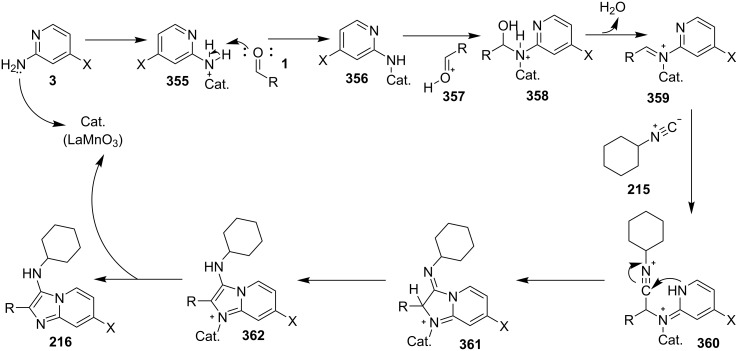
Mechanistic approach.

The mechanistic approach (Scheme 117) suggested that lanthanum chloride polarize the carbonyl bond and increased the electrophilicity of carbonyl carbon to form imine with 2-AP. The imine carbon was further attacked by isocyanide, followed by [4 + 1] cycloaddition and aromatization via 1,3-H shift to yield the final product. Owing to the efficiency of perovskite-type metal oxide in catalyzing many organic reactions Sanaeishoara et al. have reported that LaMnO_3_ NPs catalyzed the synthesis of imidazo[1,2-*a*]pyridine under neat conditions [185]. The reaction used only 0.2 mol % of the catalyst for successful conversion of 2-AP, benzaldehyde and cyclohexyl isocyanide **215** to the required product at 35 °C within 1.5 h of the reaction time (Scheme 118). However, in the absence of catalyst a very low amount of the product was formed. Non-hazardous neat reaction conditions, low catalyst loading, excellent yields with easy recoverability and reusability of the catalyst to 5 synthetic cycles made it an efficient procedure. Mechanistically, 2-AP and aldehyde led to the formation of iminium salt **359** on the surface of the catalyst **III**. This then undergoes nucleophilic addition with isocyanide **360** followed by cyclization/tautomerization to afford the target compound **216** (Scheme 119).

**Scheme 120:**
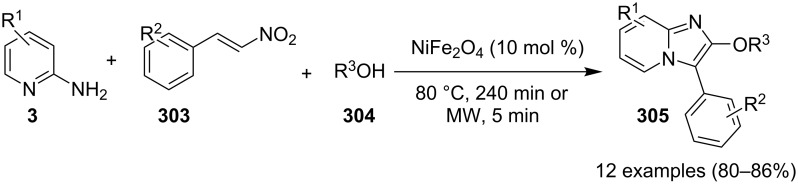
One-pot 3-CR for regioselective synthesis of 2-alkoxy-3-arylimidazo[1,2-*a*]pyridines.

Payra et al. have unprecedently reported a highly regioselective, sequential one-pot three-component synthesis of 2-alkoxy-3-arylimidazo[1,2-*a*]pyridines **305** [30]. This microwave-assisted aerobic reaction was catalyzed by nano-NiFe_2_O_4_. Compound **3**, **303**, and **304** were used as reaction substrates with alcohol **304** as one of the primary reactants (Scheme 120).

**Scheme 121:**
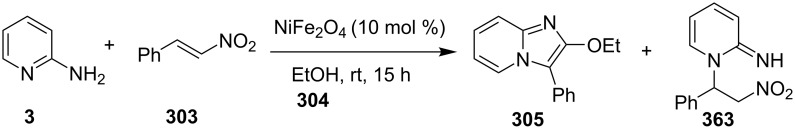
Formation of two possible products under optimization of the catalysts.

The alcohol helped in the formation of 2-alkoxy derivative, however, the introduction of alkoxy group is challenging due to poor nucleophilicity of alcohols. This was observed when the reaction was carried out in DCM in the absence of alcohol, only 2-nitroimidazopyridine was formed which does not undergo any substitution at the NO_2_ group at the later stage of the reaction. The addition of an alkoxy group and displacement of the NO_2_ group took place at an intermediate stage. The reaction was also tried with Cu-/Mn-/Co-ferrites, nano-Fe_3_O_4_, and NiO NPs but appreciable results were obtained with nano-NiFe_2_O_4_. Probably, basic Ni-sites and Fe-sites provided a synergistic effect for the production of 2-ethoxyimidazopyridines. The reaction was not feasible with the targeted outcome at room temperature as only acyclized derivative **363** mentioned in Scheme 121, was formed.

**Scheme 122:**
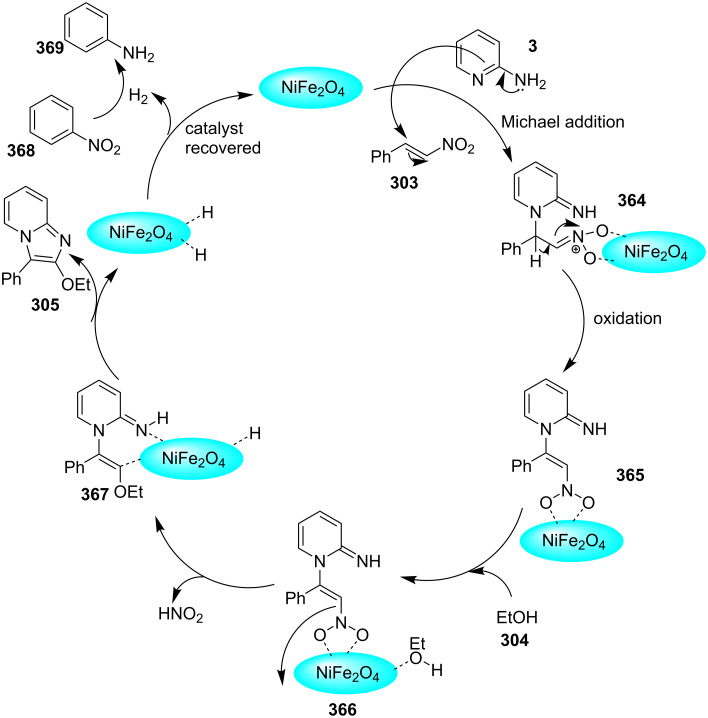
Mechanistic strategy for NiFe_2_O_4_-catalyzed reaction.

The reaction was supposed to proceed via aza-Michael addition of compound **3** to **303** that was promoted by basic Ni-sites of NiFe_2_O_4_ (Scheme 122). This was followed by Fe-promoted subsequent oxidative imination and nucleophilic substitution of the NO_2_ group and its removal as HNO_2_. At the end of the reaction, the catalyst was regenerated after the liberation of H_2_ (which reduced nitrobenzene **368** to aniline **369**). The advantage associated with NiFe_2_O_4_ was its dual behavior of catalyst as well as an oxidant.

**Scheme 123:**
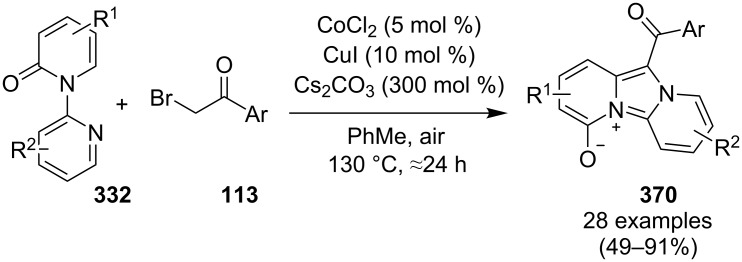
Two-component reaction for synthesizing imidazodipyridiniums.

A facile synthesis with readily available substrates for imidazo[1,2-*a*;3,4-*a*']dipyridiniums **370** was reported by Li et al., the synthesis was simple and facile involving cobalt/copper co-catalyzed system [186]. This was the only report after first-ever synthesis of this moiety in 1985. The reported strategy used 2*H*-[1,2'-bipyridin]-2-ones **332** and 2-bromoacetophenones **113** as reacting substrates (Scheme 123). Moiety **332** was the substrate for regioselective functionalization and construction of useful N-fused structures. Replacement of bromoacetophenone with a chloro or iodo derivative has revealed the 2-bromo compound to be the best substrate for this reaction.

**Scheme 124:**
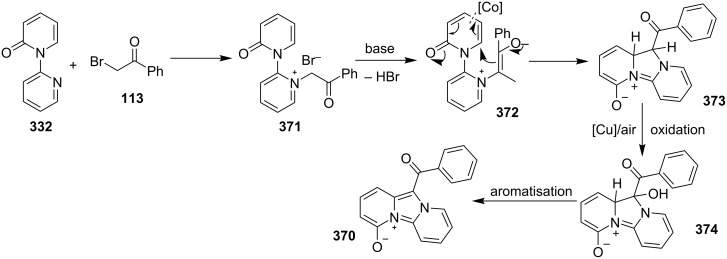
Mechanistic scheme for the synthesis of imidazodipyridiniums.

Operation of the reaction in the absence of CoCl_2_ or CuI led to substrate decomposition and either low or no yield of the product. Along with the catalyst, the presence of Cs_2_CO_3_ as a base and air atmosphere was equally important for reaction feasibility. A wide library of compounds was produced by differently substituted reacting compounds. The pyridine ring has shown good tolerance towards different groups at C-3 or C-4 position but the yield was completely suppressed with C-5 substitution due to steric hinderance. EW and EDGs at C-2 or C-3 position of the pyridyl ring were equally well tolerated. Gratifyingly groups like F, Cl, OMe and Me present on the *para*-position of the phenyl ring in aryl halides were well tolerated compared to those at *meta* or *ortho*-positions. Apart from the phenyl ring in halides thiophene afforded the product in 75% yield, but furan and aliphatic groups did not work well. The reaction followed a radical pathway that was clear by the mechanistic Scheme 124. The reaction was so robust that it worked well at 10 mmol reactant scale.

### Derivatization of imidazopyridines by C–H activation/functionalization and coupling reactions

Direct C−H functionalization/activation reactions of readily available starting materials for the construction of C–C as well as C–O and C–N bonds were highly step- and atom-economical [187]. In recent decades, intensive research efforts have been carried out for the development of feasible conditions for challenging C−H bond activation/functionalization and coupling reactions. Among many such studies, TM-catalyzed transformations constitute a far most valuable tool for efficient and economic syntheses of many complex molecules [188–190]. Such transformations embrace the step-economical construction of C–C, C–O, and C–N bonds formation from hydrocarbons or their fragments without the involvement of prior non-catalytic oxidation steps.

#### Copper-catalyzed derivatizations

**Scheme 125:**
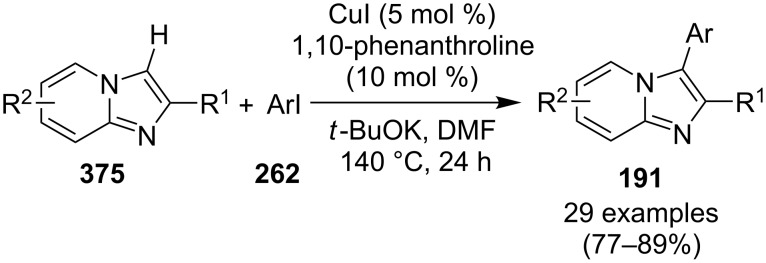
CuI-catalyzed arylation of imidazo[1,2-*a*]pyridines.

Cao and Zhao et al. have described a Cu(I)-catalyzed C–C coupling reaction for arylation of C3-unsubstituted imidazo[1,2-*a*]pyridines **375** [191]. Among different ligands and bases examined the use of 1,10-phenanthroline and *t*-BuOK as ligand and base, respectively, resulted in a significant improvement in catalytic efficiency. The reaction was carried out at 140 °C for 24 h using DMF as solvent and CuI as catalyst (Scheme 125). The reaction has well tolerated both EW and EDGs on compound **180** and aryl iodides **262**. This arylation was less sensitive to steric factors as *ortho-*, *meta-* and *para-*occupied derivatives gave the products in good yield.

**Scheme 126:**
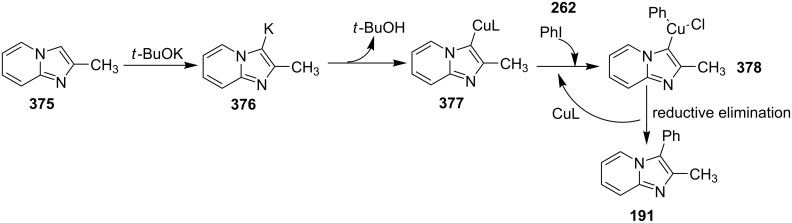
Mechanism for arylation reaction.

Apart from aryl iodides, the reaction was feasible with bromides and triflates giving a yield of the derivatized product of above 70%. Moreover, 1,4-dibromo- or diiodobenzene resulted in bis-imidazopyridine whereas 1,2-dihalobenzene did not form any bis-product and the mono-substituted product was formed in trace amounts only. The mechanism for this reaction is depicted in Scheme 126. The reaction was initiated by base-promoted deprotonation of **376**. This was followed by a number of steps and finally reductive elimination that resulted in the final product regenerating the Cu(I) catalyst to participate in the next cycle.

**Scheme 127:**
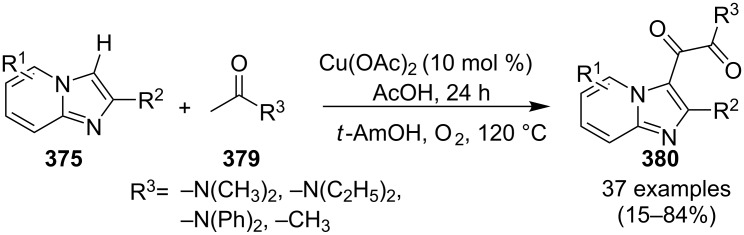
Cupric acetate-catalyzed double carbonylation approach.

Considering the immense potential associated with 1,2-dicarbonylimidazo[1,2-*a*]pyridine derivatives in the preparation of fine chemicals, Wang et al. have reported a novel approach for double carbonylation of imidazo[1,2-*a*]pyridines **375** with *N*,*N*-disubstituted acetamide or acetone **379** (Scheme 127) [192]. The reaction was catalyzed by Cu(OAc)_2_ in the presence of molecular oxygen in a regioselective fashion. The use of different additives to improve the yield has shown a beneficial effect of the acidic system. The combined use of *t*-AmOH and acetic acid (AcOH) proved to be the best additive in the presence of catalyst and O_2_ at 120 °C. The reaction was also optimized for the best Cu(II) catalyst and Cu(OAc)_2_ was found optimal among CuCl_2_, CuF_2_, CuBr_2_ and Cu(CF_3_SO_3_)_2_, also its absence was unable to carry forward the reaction.

**Scheme 128:**
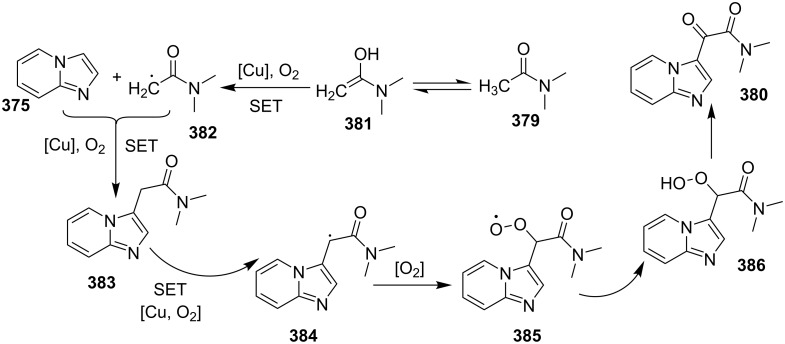
Radical mechanism for double carbonylation of IP.

The methodology was successful with various 2-unsubstituted IPs having methyl groups at different positions of the pyridine ring, whereas with iodine group at 6-position the yield was <5%. IPs substituted at 2-position with CH_3_, Ph or C(CH_3_)_3_ gave the corresponding product in 51–78% yield showing the reaction feasibility with aryl as well as alkyl groups. However, in the case of acetamides, phenylacetamide gave only traces of the product whereas, diethylacetamide and acetone were well tolerated. Mechanistic studies have revealed that oxygen of the carbonyl group was derived from molecular oxygen rather than a water molecule. The reaction was expected to proceed via radical formation from the acetamide moiety by SET **382**. The radical formed was added directly to IP, which was followed by another SET, proton transfer and SET oxidation to generate a radical intermediate **384**. This was trapped by dioxygen to give radical **385** that captured the hydrogen from reaction mixture followed by dehydration to give final compound **380** (Scheme 128).

**Scheme 129:**
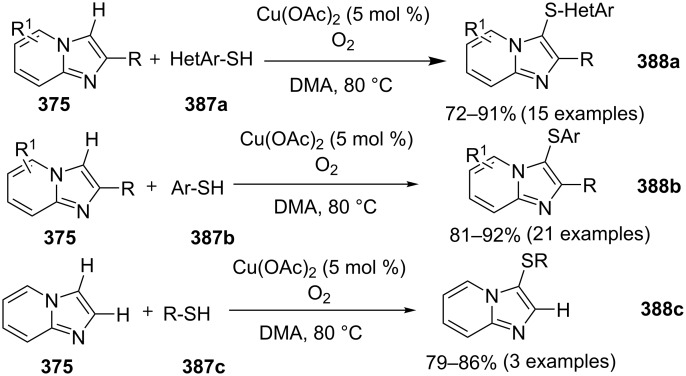
C–S bond formation reaction catalyzed by cupric acetate.

A novel approach for C–S bond formation using molecular oxygen as a greener oxidant was put forth by the group of Zheng and Qi. The reaction involved cross dehydrogenative coupling between compound **375** and heteroaryl/aryl/alkyl thiols **387a**, **387b** and **387c** under base-free conditions (Scheme 129) [193]. Optimization of various copper salts has shown Cu(OAc)_2_ to be more efficient than others. Application of different oxidants viz., air, oxone, oxygen, BQ, K_2_S_2_O_8_, and AgOAc has revealed oxygen to be the best under these reaction conditions. The protocol has well tolerated heteroaromatic/aromatic/aliphatic thiols to give 72–92% yield of the compounds.

Also, the reaction was unaffected by steric hinderance of *ortho*-substituted thiols, giving the products in good yields. Further examination of the reaction has shown it to be regioselective as thiolation took place selectively at C-3 position of the unsubstituted imidazo[1,2-*a*]pyridine. Furthermore, the reaction conditions were also feasible for 3-methyl-6-phenylimidazo[2,1-*b*]thiazole that resulted in a moderate yield of the products with 4-methyl and 4-methoxy-substituted arylthiols (39 and 46%, respectively).

**Scheme 130:**
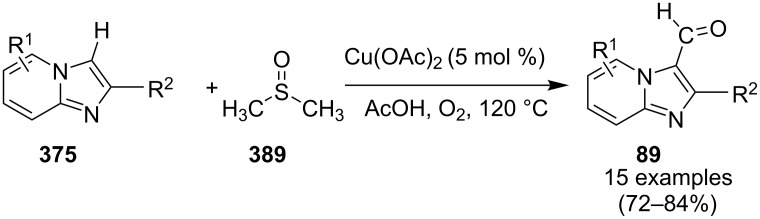
Cupric acetate-catalyzed C-3 formylation approach.

After reporting a palladium-catalyzed arylation of IP Cao et al. have reported a novel and an efficient route for C-3 formylation of imidazo[1,2-*a*]pyridines catalyzed by cupric acetate (Scheme 130) [127]. The group has exploited the ability of dimethyl sulfoxide (DMSO, **389**) to be used as both a carbon source as well as solvent. Compound **389** was found to act as an important carbon source for C=O, Me, SMe, SO_2_CH_3,_ and CN formation.

**Scheme 131:**
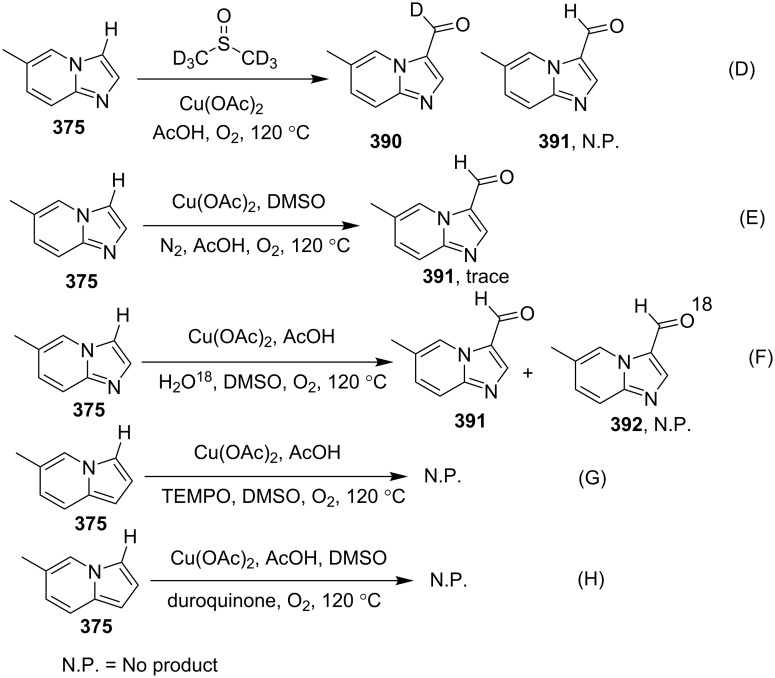
Control experiments for signifying the role of DMSO and oxygen.

**Scheme 132:**
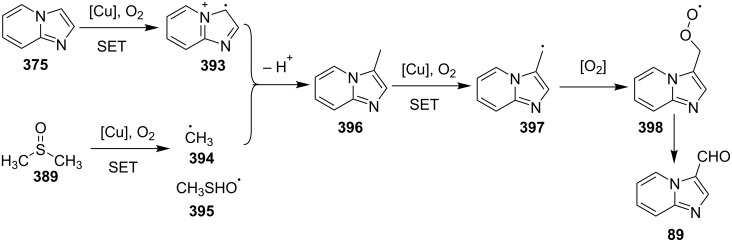
Mechanism pathway.

**Scheme 133:**
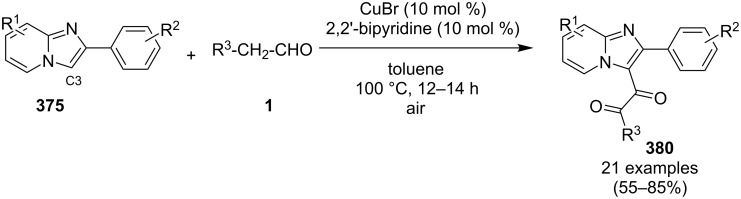
Copper bromide-catalyzed CDC reaction.

The group has initially tried the reaction of 2-*tert*-butylimidazo[1,2-*a*]pyridine using Cu(OAc)_2_ as a catalyst under an oxygen atmosphere at 120 °C and obtained the formylated product in 46% yield. In order to improve the yield, different additives were used and AcOH was found to increase the yield up to 82%. However, in the absence of Cu(OAc)_2_ no product was detected proving the essentiality of a copper catalyst. The protocol has well tolerated a variety of 2-substituted imidazo[1,2-*a*]pyridines with a phenyl ring, a sterically bulky C(CH_3_)_3_ group and methyl groups at different positions (like 2, 6, 7 and 8). This formylation is regioselective as the C-3 position was functionalized even by using 2-unsubstituted IPs (D and E, Scheme 131). The control experiments performed by the group revealed compound **389** and oxygen to be the carbon-hydrogen and oxygen source of the aldehyde respectively (F, Scheme 131). The reaction proceeded by a radical pathway which was delineated by the use of TEMPO and duroquinone (radical inhibitors) that did not form any product (G and H, Scheme 131). Mechanistically, simultaneous SET oxidation of **389** and **375** generated two radicals which were coupled together to generate methylated intermediate **396**. This intermediate was again converted to a radical by SET [O] which was trapped by oxygen to give a peroxy radical that finally get converted in the desired product **89** (Scheme 132). Shakoor et al*.* have demonstrated a copper-catalyzed double-oxidative cross-dehydrogenative procedure for the synthesis of C-3 coupled imidazo[1,2-*a*]pyridine systems [194]. This work was significant in terms of direct cross-dehydrogenative coupling (CDC) which has not been reported earlier. The reaction was carried out between C(sp^2^)–H of aldehyde **1** and C3(sp^2^)–H of **375** (Scheme 133).

**Scheme 134:**
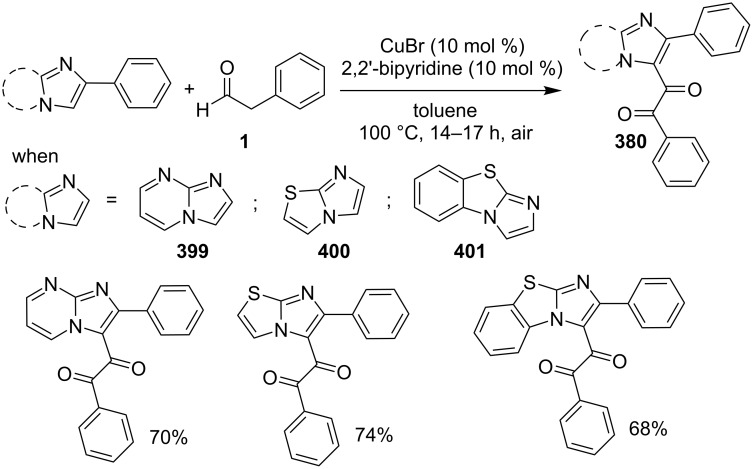
Extension of the substrate scope.

The reaction was studied with different metal salts but the best results were obtained with CuBr. Among the different oxidants and ligands studied, air and 2,2-bipyridine were found to be optimal giving the best results. The control experiments performed necessitate the aerobic condition along with copper bromide, as in the absence of any of them reaction did not proceed. Exploration of reaction scope interestingly resulted in the use of aryl aldehydes over aliphatic ones which did not result in product formation, also the presence of both EW as well as EDGs on **375** have well tolerated the reaction. Moreover, the scope was extended to many other imidazo heterocycles like imidazo[1,2-*a*]pyrimidine **399**, imidazo[2,1-*b*]thiazole **400** and benzo[*d*]imidazo[2,1-*b*]thiazole **401** which gave appreciable yield with longer reaction time (Scheme 134).

**Scheme 135:**
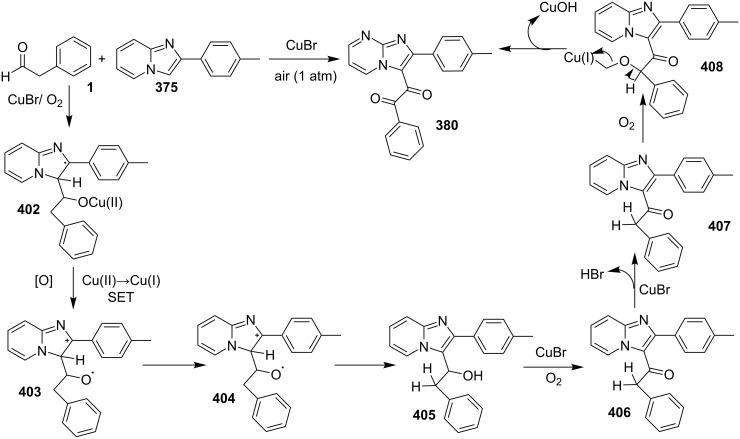
Plausible radical pathway.

**Scheme 136:**
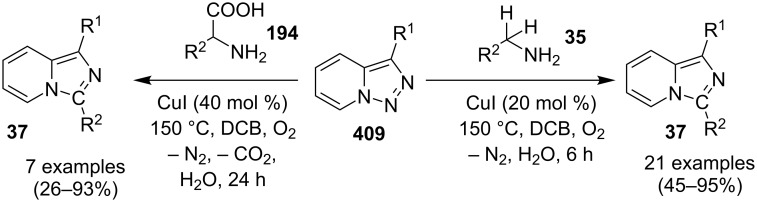
Transannulation reaction for the synthesis of imidazo[1,5-*a*]pyridines.

**Scheme 137:**
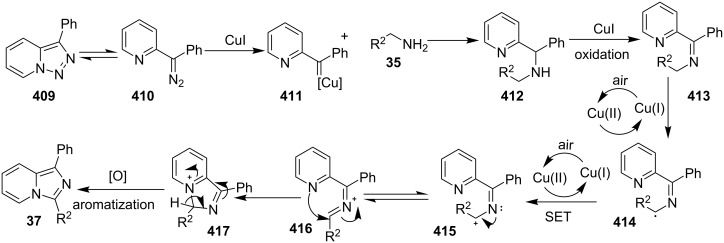
Plausible reaction pathway for denitrogenative transannulation.

Furthermore in order to explore reaction mechanism TEMPO was used which indicated the radical pathway of the reaction, as the application of TEMPO inhibited the reaction. The reaction was thought to be initiated by the attack of imidazo[1,2-*a*]pyridine on carbonyl of phenylacetaldehyde by a radical mechanism via a single electron transfer (SET) route. This was followed by oxidation/oxygen insertion giving the final dicarbonylated product (Scheme 135). Joshi et al. have developed a synthetic strategy for imidazo[1,5-*a*]pyridines **37** via denitrogenative transannulation reaction [195]. They have reported the use of a copper salt (CuI) as a catalyst at elevated temperature in a closed tube. The reaction represents good functional group tolerance for the starting materials with both amines **35** and amino acid derivatives **194** (Scheme 136). The reaction was also found to be successful with α-amino acids, like phenyl glycine, alanine, and leucin via decarboxylation and transannulation. Though, the strategy was not applicable for aza-heterocyclic and simple aliphatic amines as they were decomposed during the reaction. The formation of the product did not occur in the absence of a catalyst. Also, under neat conditions and an atmosphere of oxygen and nitrogen a drastic decrease in the product yield took place. The reaction proceeds via denitrogenation followed by the formation of Cu–carbene complex **411** which underwent migratory insertion/oxidative dehydrogenation/aromatization to yield the final product **37**. Oxidation is carried out by the molecular oxygen from the air (Scheme 137).

**Scheme 138:**
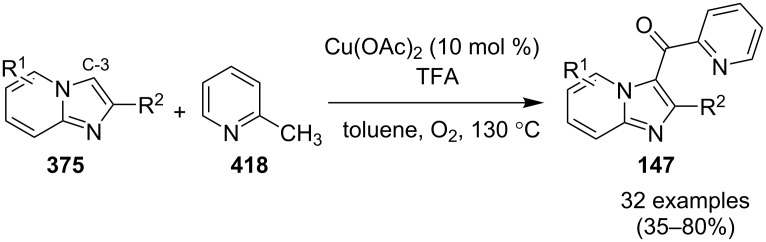
Cupric acetate-catalyzed C-3 carbonylation reaction.

In continuation of the work towards C(sp^2^)–H bond activation of IPs Lei et al. have reported a novel copper-catalyzed cross coupling of **180** with methylheteroarenes **418** [196]. This reaction involved regioselective C-3 carbonylation of IP. The conditions used were found to be inspired by their previous work. Cu(OAc)_2_ was used as a catalyst in the presence of trifluoroacetic acid (TFA) in a sealed tube with oxygen atmosphere to gave a good yield of products **147** (Scheme 138). The application of different oxidants like TBHP, K_2_S_2_O_8_, DDQ, and AgOAc has shown O_2_ to be the best for this reaction.

**Scheme 139:**
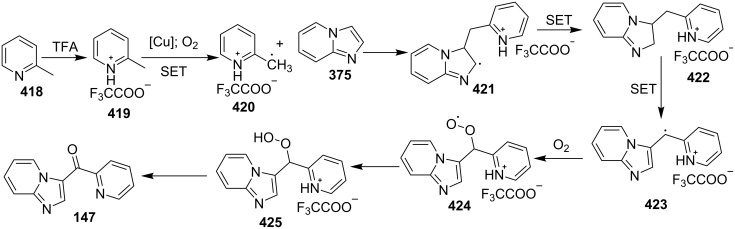
Plausible mechanism for regioselective C-3 carbonylation.

The pyridine ring of IP has well tolerated both EW and EDGs. Moreover, the 2-position of IP, substituted with methyl, phenyl, and *t*-Bu worked well, whereas in the presence of a CF_3_ group the reaction could not be performed. On the other hand methyl heteroarenes substituted with both EW and EDGs along with 2-methylquinoline, 2-methylpyrazine, 2-chloro-3-methylpyrazine and 2,5-dimethylthiazole reacted well to afford good yields of the desired compounds. Further exploration of the reaction mechanism has revealed a radical-driven pathway with the initial coupling of both the reactants rather than the formation of benzaldehyde from methylheteroarenes (Scheme 139).

**Scheme 140:**
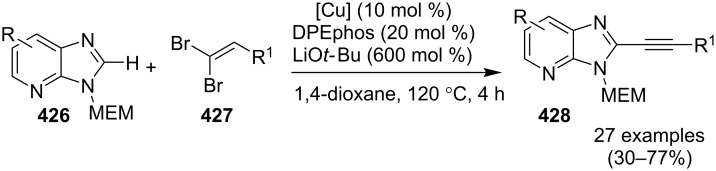
Alkynylation reaction at C-2 of 3*H*-imidazo[4,5-*b*]pyridines.

A direct C-2 alkynylation of 3*H*-imidazo[4,5-*b*]pyridines **426** was reported by the group of J. Aziz. The group has used Cu(OAc)_2_ or CuBr·SMe_2_ (copper bromide in a dimethyl sulfide complex) as catalyst depending upon the nature of substituents present on the reacting substrates [197]. The reaction involved DPEphos as a phosphine ligand and LiO*t-*Bu as a base in 1,4-dioxane at 120 °C (Scheme 140). The protecting group present at the N-3 position of the IP moiety was coupled with gem-dihaloalkenes **427** via C–H alkynylation. Initially, *p*-methoxybenzyl (PMB) was used as a protecting group but its deprotection led to degradation of the formed product.

**Scheme 141:**
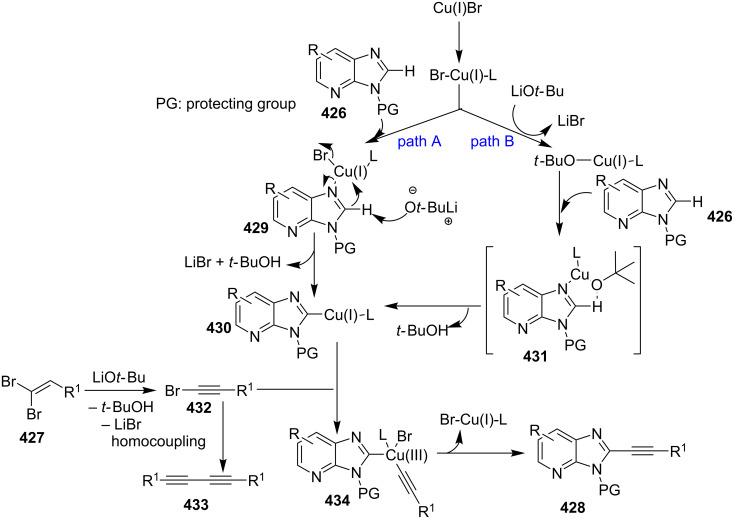
Two-way mechanism for C-2 alkynylation of 3*H*-imidazo[4,5-*b*]pyridines.

Thus, it was changed to 2-methoxyethoxymethyl ether (MEM) as a protecting group which could be easily deprotected to give a moderate yield of the final compound without degradation. Although the reported methodology was not compatible with the nitrile functional group it has tolerated a wide variety of other EW and EDGs. The reaction was supposed to proceed via either of the two pathways reported by the group based on literature reports; the mechanism is depicted in Scheme 141.

#### Palladium-catalyzed derivatizations

**Scheme 142:**
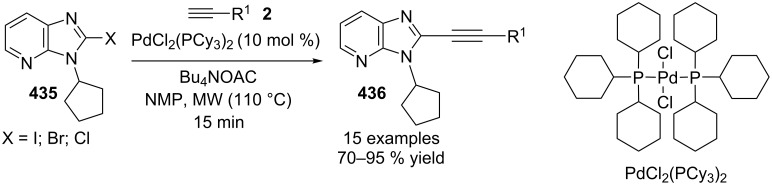
Palladium-catalyzed SCCR approach.

Sajith and Muralidharan have put forth the very first Sonogashira cross-coupling reaction (SCCR) using 3-alkyl-2-haloimidazo[4,5-*b*]pyridine **435** as one of the heteroaromatic substrates [198]. The advantageous aspect of their methodology lied in the development of an efficient protocol involving copper- and amine-free SCCR using a palladium catalyst (Scheme 142). The formation of homocoupled product from copper acetylide was a major drawback that led them to modify the reaction to copper-free conditions and used PdCl_2_(PCy_3_)_2_ as an efficient catalyst. This catalyst was found to be more effective than Pd(PPh_3_)_4_, PdCl_2_(PPh_3_)_2_, PdCl_2_(CH_3_CN)_2_ and Pd(dba)_3_ with NMP (*N*-methylpyrrolidinone) as solvent under copper-free and MW (110 °C) conditions. Furthermore, they have used tetrabutylammonium acetate (Bu_4_NOAc) as a mild base to deprotonate the acidic proton of the alkyne **2** and for the formation of the Pd(0) species.

**Scheme 143:**
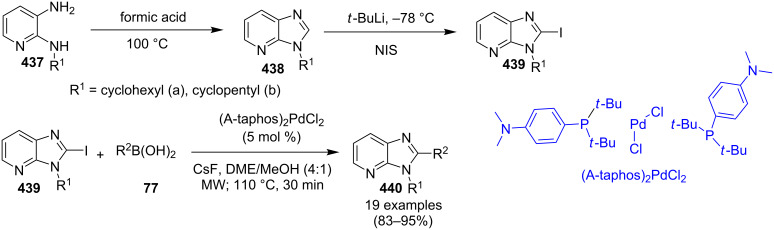
Palladium-catalyzed Suzuki coupling reaction.

The reaction has represented a wide functional group tolerance. The iodo-substituted imidazo[4,5-*b*]pyridine derivatives were found to react in a much faster way as compared to bromo and chloro-substituted ones. This might be due to the easy removal of the iodide species. On the other hand, the reaction proceeds more efficiently with aromatic alkynes than with the aliphatic ones. The combination of electronic and steric properties associated with the reported catalyst was found to enhance its activity. The reaction was expected to follow oxidative addition, transmetallation and reductive elimination steps which were easier to perform due to the presence of less bulky ligands on the metal center. Furthermore, the reaction involved the formation of a monoligated species which was stabilized by electron-rich ligands attached to the Pd center. They have simultaneously developed a MW-assisted palladium-catalyzed Suzuki coupling reaction between substituted aryl/heteroarylboronic acids **77** and 3-substituted-2-iodo-3*H*-imidazo[4,5-*b*]pyridine derivatives **439** to synthesize 3-substituted-2-aryl/heteroaryl imidazo[4,5-*b*]pyridines [199]. The iodo derivative used in this procedure was initially synthesized at −78 °C (Scheme 143). This compound **439** was used as a key intermediate in the Suzuki type reaction in the presence of (A-taphos)_2_PdCl_2_ (as a catalyst) along with CsF as a base under nitrogen atmosphere.

**Scheme 144:**
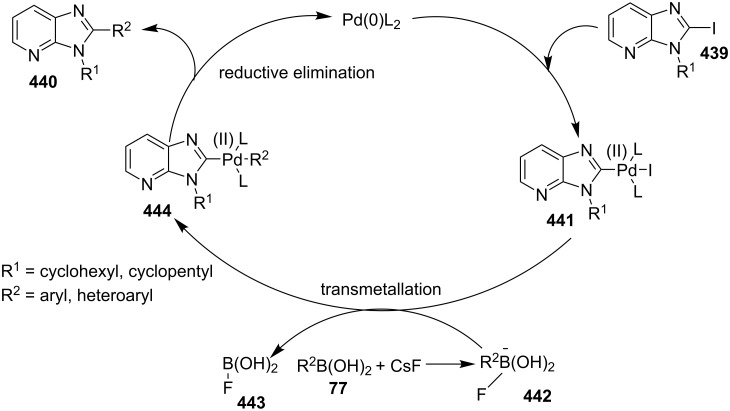
Reaction mechanism.

The dimethyl amino groups present in the catalyst increased the basicity of the phosphine ligand attached to the palladium center thus facilitating the synthesis of the coupled product by reducing or avoiding the formation of dehalogenated byproducts. The use of a strong base helped in the formation of a highly reactive borate species which again helped in the reduction of dehalogenated side products by enhancing the rate of the transmetallation step. Mechanistically, the reaction involved an oxidative addition of **439** to the Pd(0) complex and form an organo-Pd(II) species **441**. This step was followed by a transmetallation in the presence of base–borate complex **442**. Finally, reductive elimination of this intermediate led to the formation of the coupled product (Scheme 144).

**Scheme 145:**
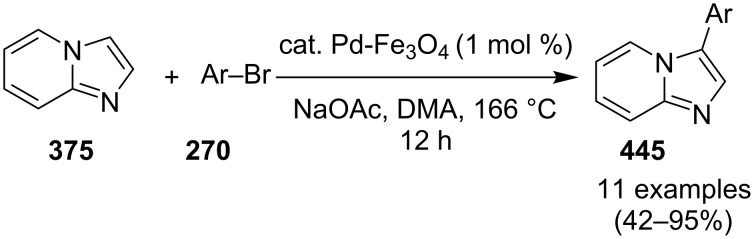
A phosphine free palladium-catalyzed synthesis of C-3 arylated imidazopyridines.

An efficient, phosphine-free methodology for C–H arylation of imidazo[1,2-*a*]pyridine derivatives **375** was reported by the group of Lee and Chung. They have used Pd-Fe_3_O_4_ nanocrystals as a heterogeneous catalyst that was synthesized by a two-step thermal decomposition process. The reaction was highly regioselective with exclusive arylation at the C-3 position of the heteroarene (**375**, Scheme 145) [200].

**Scheme 146:**
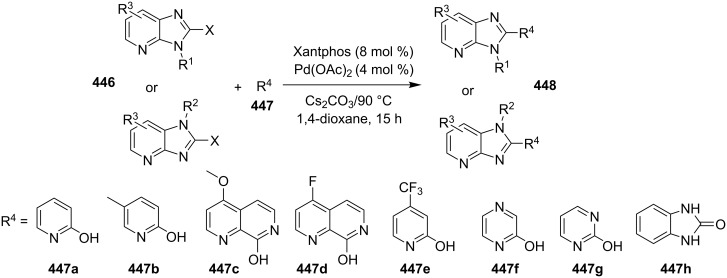
Palladium-mediated Buchwald–Hartwig cross-coupling reaction.

The method resulted in a good yield of the product with simple and easy recovery of magnetic NPs. The arylation of heteroarenes by this methodology required a bimetallic catalytic system as the absence of catalyst or the presence of only Fe_3_O_4_ did not result in product formation. The bromo arenes **270** used in this process have wider electronic and steric tolerance. Moreover, polycyclic bromides including naphthalenes and anthracenes were found to be suitable for this regioselective synthesis. The most advantageous part of the protocol was recyclability of the used catalyst up to 10 synthetic cycles without significant loss in its activity. The catalytic ability of palladium for C–N bond formation reactions made it a catalyst of choice for the construction of (hetero)arylamines. Encouraged by this activity and pharmaceutical importance of imidazo[4,5-*b*]pyridines (I[4,5-*b*]Ps) Khader and Sajith et al. have demonstrated a palladium-mediated Buchwald–Hartwig cross coupling reaction [201]. This approach involved cross coupling of a wide range of enolizable heterocycles **447** with I[4,5-*b*]Ps **446** (Scheme 146). This methodology involved the introduction of pyridines at C-2 position of 2-halo-substituted I[4,5-*b*]Ps. Initially, they have tried this coupling reaction via aromatic nucleophilic substitution (S_N_Ar) but it did not result in product formation. Different halo-substituted I[4,5-*b*]Ps were examined (viz., chloro, bromo, and iodo) for this coupling which revealed the iodo substituent to be highly reactive and thus happened to be dehalogenated rather than coupling at the C-2 position. Furthermore, substitution with other two halogens resulted in product formation although in lesser amount using Pd(OAc)_2_/BINAP (catalyst/ligand) and Cs_2_CO_3_ as a base in 1,4-dioxane.

**Figure 7:**
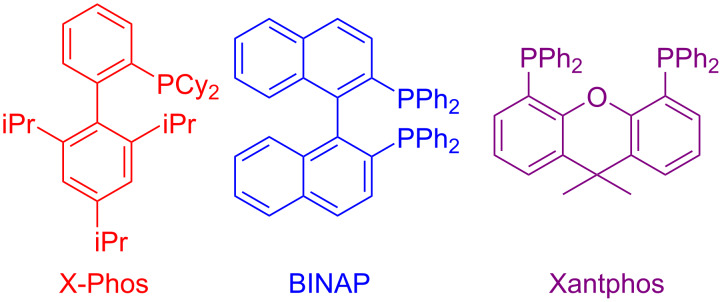
Structure of the ligands optimized.

In order to increase the overall yield exploration of different ligands (X-Phos, BINAP, and Xantphos, Figure 7) was carried out which showed that appropriate steric and electronic properties of ligand played a crucial role in this conversion. Finally, the use of Xantphos has resulted in a regioselective C-2 amination of I[4,5-*b*]Ps using various enolizable heterocycles in appreciable yield.

**Scheme 147:**
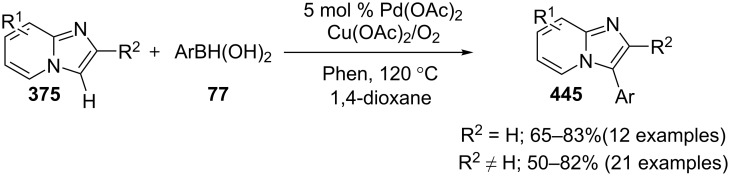
Palladium acetate-catalyzed direct arylation of imidazo[1,2-*a*]pyridines.

Zhao et al. have reported a regioselective arylation of compound **375** by a direct method catalyzed by palladium acetate [202]. The process ruled out any requirement for a pre-functionalisation of the reaction substrates. Successful arylation at the C-3 position of unsubstituted imidazo[1,2-*a*]pyridine with **77** makes this protocol highly regioselective. Application of Cu(OAc)_2_ as oxidant and oxygen as co-oxidant in the presence of palladium acetate proved to be ideal for this synthesis (Scheme 147). However, the reaction did not result in product formation by using BQ and oxone as oxidant.

**Scheme 148:**
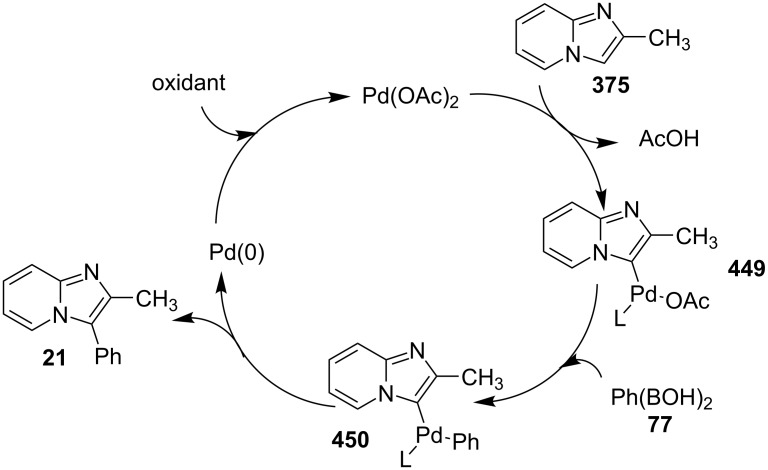
Palladium acetate-catalyzed mechanistic pathway.

**Scheme 149:**
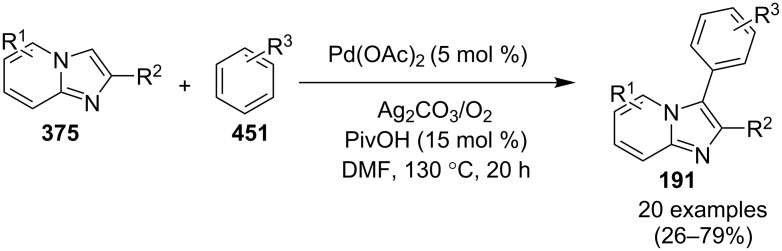
Palladium acetate-catalyzed regioselective arylation reported by Liu and Zhan.

The reaction was depicted to be compatible with differently substituted arylboronic acids and a variety of 2-substituted imidazo[1,2-*a*]pyridines having both EW and EDGs, though the reaction yield was affected by steric factors as *ortho*-substituents gave moderate yields. The reaction was supposed to proceed via C–H cleavage at the 3-position of **375** followed by coordination from **77** and reductive elimination to yield the final product (Scheme 148). The group of Wang and Liu [203] has developed a more facile and versatile approach than that of Cao et al. [204] for the regioselective arylation of **375**. The noteworthy aspect of their methodology was Pd(OAc)_2_-catalyzed cross coupling with unactivated arenes **451**. This kind of direct dehydrogenative cross coupling has gained much attention due to the reduced cost of pre-functionalization and increased atom economy. The reaction has utilized a combination of Ag_2_CO_3_/O_2_ as an effective oxidant for reoxidation of Pd(0) during the course of reaction (Scheme 149). Moreover, use of pivalic acid as additive and DMF as solvent was found to create the best reaction conditions.

**Scheme 150:**
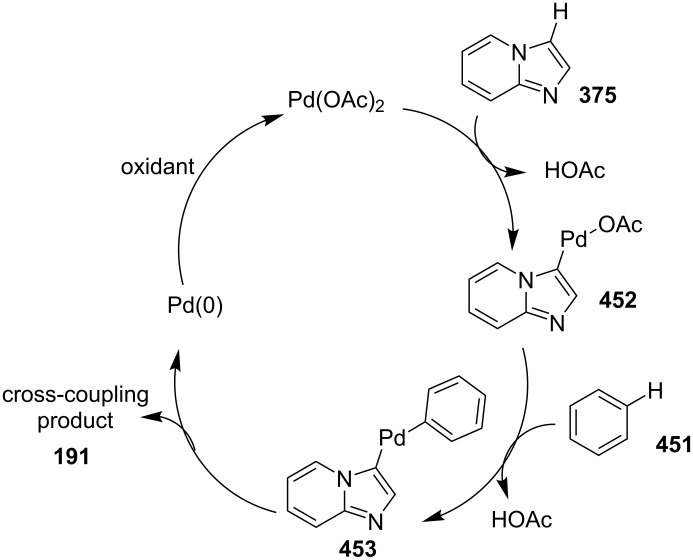
Mechanism for selective C-3 arylation of IP.

The method appeared to have good functional group tolerance on both IPs and benzene with a maximum yield of about 79%. However, the reaction was affected by steric hinderance as the reaction of *ortho* and *meta*-xylene occurred at less hindered position and that of mesitylene resulted in sluggish rate with low yield (26% and 29%) of the product. This protocol was also regioselective as it gave C3-substituted product over C2-arylated product. The reaction was thought to proceed by easy abstraction of hydrogen from IP by Pd(OAc)_2_ followed by concerted metallation and deprotonation of arene which might be the rate determination step of the reaction. The intermediate **453** thus formed undergoes reductive elimination to give the final product. Finally, Pd(0) was reoxidized to Pd(II) for completion of the reaction and beginning the new reaction cycle (Scheme 150).

**Scheme 151:**
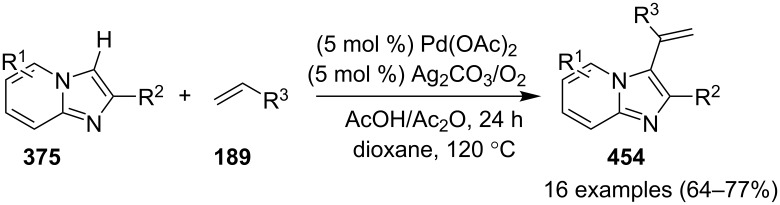
Pd(II)-catalyzed alkenylation reaction with styrenes.

Cao et al. in continuation of their work, have developed Pd(II)-catalyzed stereo- and regioselective approach for the oxidative C-3 alkenylation of **375** (Scheme 151) [204]. In order to optimize the reaction conditions, different oxidants were tried such as BQ, DDQ, *t*-BuOOBz, etc. but the combination of O_2_ and Ag_2_CO_3_ was found to be more effective. Thus the reaction has avoided the formation of environmentally hazardous byproducts and also reduced the use of silver salts as the sole oxidant.

**Scheme 152:**
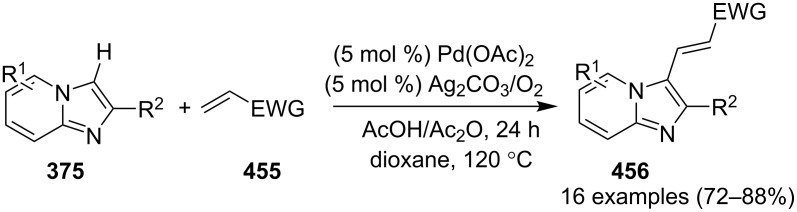
Pd(II)-catalyzed alkenylation reaction with acrylates.

The reaction yield was further increased by the use of the additives AcOH/Ac_2_O. The protocol was highly regioselective, even when carried out with 2,3-dihydroimidazopyridines it gave the C-3-substituted product. This alkenylation has tolerated both styrenes as well as acrylates **455**, however, styrenes gave the α-product and the β-product was obtained with the later one **456** (Scheme 152).

**Scheme 153:**
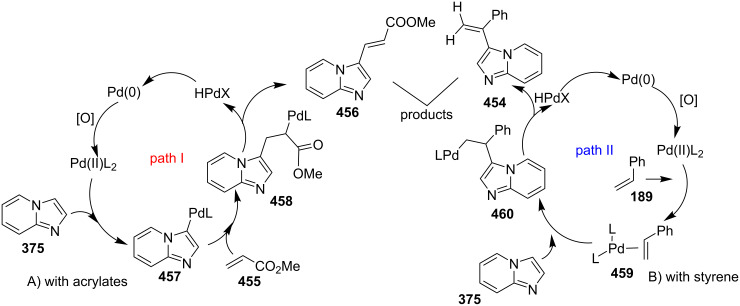
A two way mechanism.

For the formation of two different types of product, two mechanistic pathways were reported for the reaction. Reaction with **455** involved initial palladation at C-3 position of the IPs **375** (Scheme 153). This intermediate **457** then reacted with **455** followed by β-elimination to give desired β-product **456**. On the other hand, the reaction with styrene involved formation of Pd-styrene complex **459** which then underwent a nucleophilic attack by IP (**375**) followed by β-elimination and resulted in the formation of α-product **454** (Scheme 153).

**Scheme 154:**
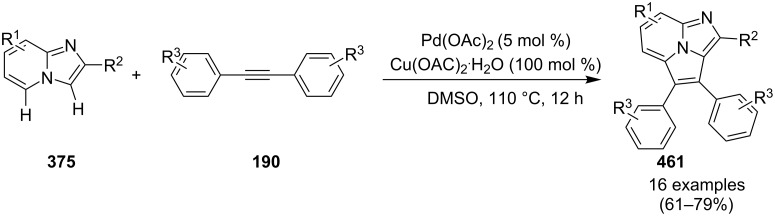
Double C–H activation reaction catalyzed by Pd(OAc)_2_.

Synthesis of polycyclic aromatic hydrocarbons (PAHs) by direct C–H activation without using any directing group has encouraged the group of M. Ghosh to develop a palladium-catalyzed double C–H bond activation of **375** [205]. Among different palladium salts used, Pd(OAc)_2_ was found to be most effective and the absence of catalyst resulted in failure of the reaction (Scheme 154). The group has also examined the effects of different oxidants viz. BQ, DDQ, Ag_2_CO_3_, AgOAc, O_2,_ etc. along with various additives (like pivalic acid, TBAB). The reaction was found to proceed well by using copper acetate as oxidant whereas the effect of additive was negligible on the reaction yield.

**Scheme 155:**
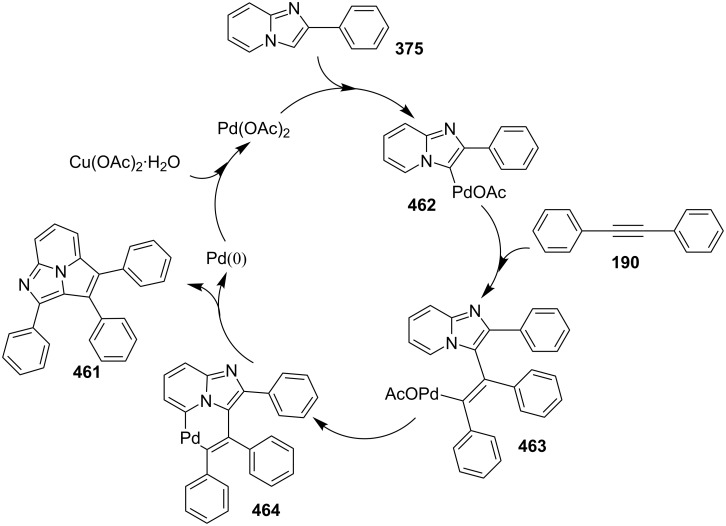
Probable mechanism.

IP substrates with a phenyl ring at the 2-position and substituted with both EW and EDGs were found to give a good yield of the products. Also, unsubstituted and *para*-substituted diphenylacetylenes resulted in a moderate to good yield of the reaction product. However, internal alkynes like 1-phenyl-1-propyne, methyl phenylpropiolate, dimethyl acetylenedicarboxylate, and 4-octyne did not result in desired product formation showing an incompatibility of the unsymmetrical alkynes. This reaction involved the formation of vinylic palladium(II) intermediate **463** which undergoes C–H activation and resulted in the 6-membered palladacycle **464**. Reductive elimination of this palladacycle synthesized the final compound **461** along with Pd(0) species. The so formed Pd(0) was reoxidized to Pd(II) thereby completing the catalytic cycle (Scheme 155).

**Scheme 156:**
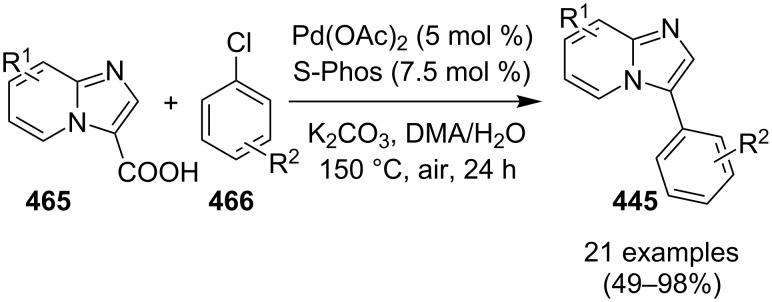
Palladium-catalyzed decarboxylative coupling.

Mu et al. have reported a palladium-catalyzed decarboxylative coupling of heteroaromatic carboxylic acid **465** with (hetero)aryl chloride **466** under aerobic conditions [206]. Decarboxylative reactions were found to be important due to their access to various valuable product classes. Arylation of imidazo[1,2-*a*]pyridines by this method was reported initially only by the group of Nandi [207]. Their methodology suffered from certain limitations like moisture and air sensitivity along with limited substrate scope for aryl bromides. The method reported by Mu et al. was successfully carried out under aerobic conditions using DMA/H_2_O (40:1) as solvent media in the presence of K_2_CO_3_ as a base (Scheme 156). Furthermore, the reaction found to give a maximum yield of 96% of coupling product in the presence of the ligand S-Phos in contrast to other ligands like X-Phos, Ru-Phos, and PCy_3_. The reaction has wider substrate compatibility ranging from that of electron neutral/rich or poor reactants along with sterically hindered aryl/heteroaryl chlorides.

**Scheme 157:**
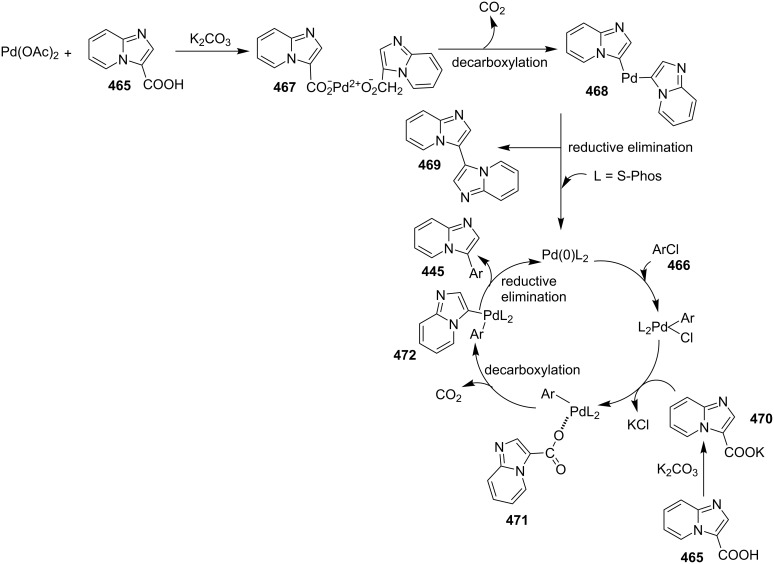
Mechanistic cycle for decarboxylative arylation reaction.

**Scheme 158:**
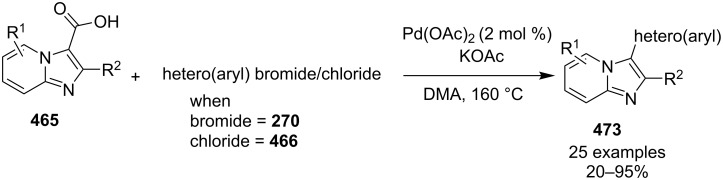
Ligand-free approach for arylation of imidazo[1,2-*a*]pyridine-3-carboxylic acids.

**Scheme 159:**
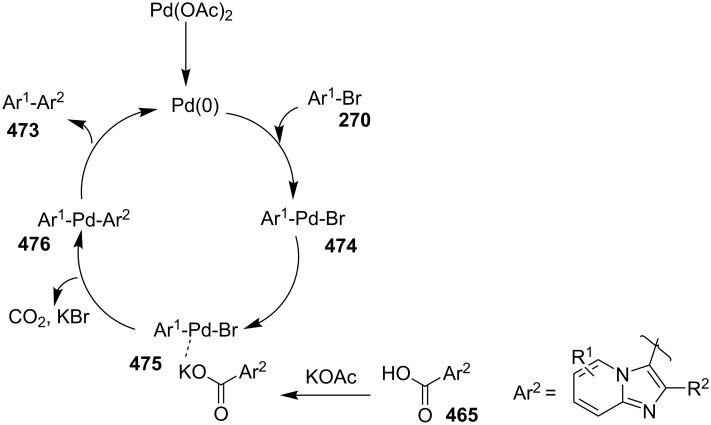
Mechanism for ligandless arylation reaction.

**Scheme 160:**
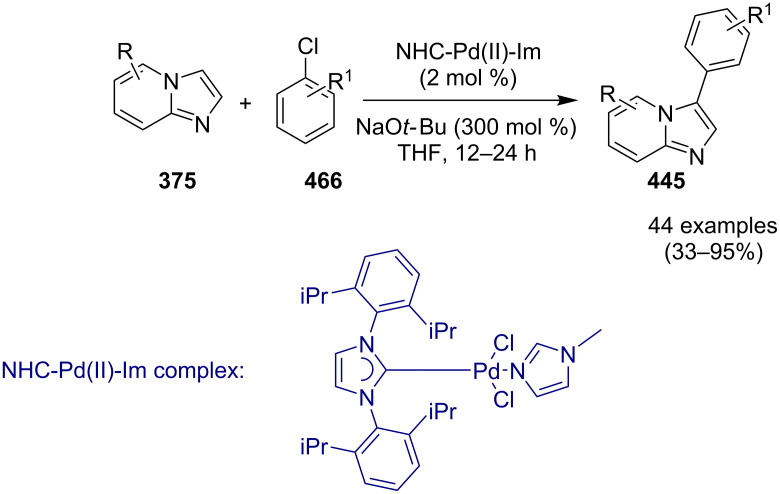
NHC-Pd(II) complex assisted arylation reaction.

Addition of a small amounts of water to the solvent media has efficiently promoted the product yield. The reaction was promoted by a decarboxylative reaction and reductive elimination of IP to afford homocoupling product **469** and catalytically active Pd(0)L_2_ with the assistance of the S-Phos ligand. This active catalyst was then combined with aryl chloride by oxidative addition followed by a ligand exchange reaction with carboxylic acid anion. This was followed by decarboxylation and reductive elimination reaction to generate the final arylated product with regeneration of Pd(0)L_2_ to complete the catalytic cycle (Scheme 157). The Group of Karale has unprecedentedly reported a general protocol for ligand-free Pd(OAc)_2_-catalyzed arylation of **465** (Scheme 158) [208]. Palladium-catalyzed arylation reactions suffered from limitations of substrate scope and use of C-2-substituted imidazo[1,2-*a*]pyridines. The ligandless approach found to be widely used in the industries thus it became a method of choice in synthetic chemistry. The method developed by the group did not require any additive or ligand for the success of this reaction. However, the presence of a base (KOAc) resulted in the cross-coupled product in good yield with only negligible homocoupled byproduct. A decrease in the catalyst loading from 5 to 2 mol % resulted in an increased yield of the desired product with complete suppression of byproducts. The reason for this might be the formation of insoluble Pd black with higher amount of loading that resulted in decreased efficiency of the Heck reaction. The yield of reaction was also affected by the stoichiometry of the reactants as variation in the loading of IP from 1.4 to 1.5 or 1.6 equiv resulted in decreased homodimerization of the reactant. The reaction has shown good functional group tolerance with both EW and EDGs, the result was moderate to good and the yield of the products varied with reaction time. The reaction was also compatible with aryl chloride/bromide and with aryl/heteroaryl bromides. Despite wider applicability, the time consumed by this process with some substituents was very large which varies from several hours to days. The reaction involved an oxidative addition of Pd(0) to aryl bromide which was further coordinated with the carboxylate species. Decarboxylation of this intermediate followed by reductive elimination led to the formation of the 3-arylated product along with the reduction of Pd(II) to Pd(0) (Scheme 159). The group of Liu and He have reported a direct C–H bond arylation reaction of **375** with aryl chloride **466** [209]. The group has developed an N-heterocyclic carbine–Pd(II) [NHC-Pd(II)] complex which was found to be effective towards C–C, C–N coupling and C–H arylation reactions (Scheme 160). The NHC–Pd(II) complex developed has air and moisture stability and resulted in a direct arylation reaction without the use of any directing group. The reaction was found to proceed with efficiency in the presence of NaO*t*-Bu as compared to other bases examined for the reaction. The amount of base played a crucial role in the reaction yield, as decreasing the loading from 3 equiv to 2 equiv resulted in a decreased yield of the product.

Furthermore, the use of Pd(OAc)_2_ or PdCl_2_ did not form any product. The substrate effect was very interesting in the protocol as the reaction of unsubstituted IPs with aryl chlorides resulted in best yield when 2-/3-/4-fluorophenyl chloride were used rather than those with EDGs like methoxy and methyl. Aryl chlorides substituted with strong EWGs like NO_2_, CN and acetyl did not lead to the formation of the desired compounds. However, with substitution on IPs ary chlorides bearing methoxy and methyl substituents resulted in excellent product yield.

**Scheme 161:**
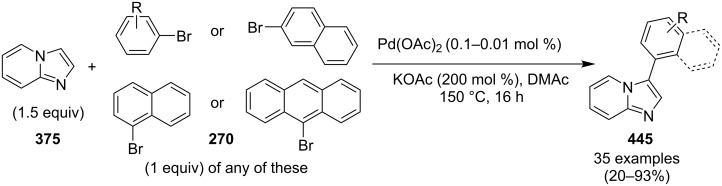
C-3 arylation of imidazo[1,2-*a*]pyridines with aryl bromides catalyzed by Pd(OAc)_2_.

To decrease the loading of palladium as a catalyst and to avoid the use of the phosphine ligand Hai Y. Fu et al. have reported a ligand-free Pd(OAc)_2_-catalyzed C-3 arylation of **375** with **270** [210]. The reaction also required potassium acetate as a base with DMAc as the solvent system. The use of other acetate bases viz., CsOAc and NaOAc were also found to be compatible with the reported conditions as the acetates were in consistence with a concerted metalation–deprotonation (CMD) pathway. The reaction was successful with only 0.1–0.01 mol % loading of the catalyst with broadly substituted aryl bromides having an EW as well as EDGs at *ortho*, *meta* and *para*-positions (Scheme 161). Use of bromonaphthalene/anthracene was also well tolerated as the product was obtained in >50% yield. Moreover, the use of heteroaryl bromides and arylpoly bromides resulted in high product yield of up to 92%. The reported protocol was not limited to imidazo[1,2-*a*]pyridine but 6-carbonitrile substituted compounds also gave the coupled product in 64 and 72% yield, with 4-bromobenzaldehyde or 2-bromonaphthalene, respectively. The advantageous point of the reaction was multi-solvent compatibility as it tolerated petan-1-ol, diethyl carbonate, and cyclopentyl methyl ether as well.

**Scheme 162:**
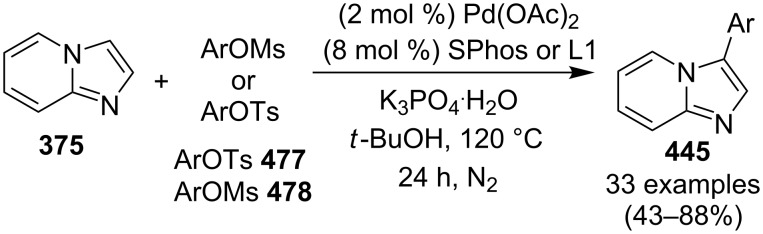
Pd(II)-catalyzed C-3 arylations with aryl tosylates and mesylates.

Choy and Luk have reported a Pd(II)-catalyzed direct C-3 arylation of **375** with aryl tosylates **477** and mesylates **478** [211]. This was the very first report on Pd-catalyzed direct arylation using tosylates and mesylates. A limited number of reports on direct C-3 arylation has paved the way for further development in this field. Combination of Pd(OAc)_2_ with 2-dicyclohexylphosphino-2',6'-dimethoxybiphenyl (SPhos) provided a good catalytic system for tosylate coupling and Pd(OAc)_2_ with 2-(2-(diisopropylphosphino)phenyl)-1-methyl-1*H*-indole (L1) found to be optimal in the case of mesylate coupling (Scheme 162). This reaction also required K_3_PO_4_·H_2_O as a base with *t*-BuOH as a polar-protic solvent.

**Scheme 163:**
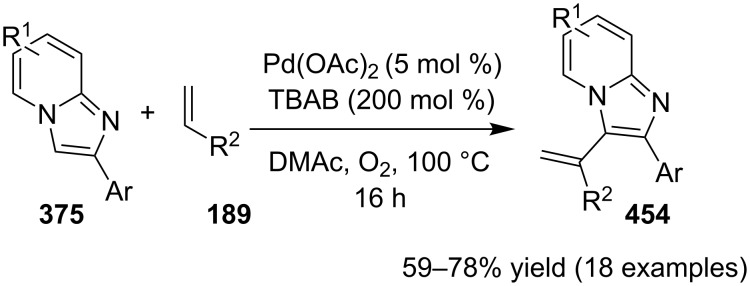
CDC reaction for the synthesis of imidazo[1,2-*a*]pyridines.

Apart from these requirements the loading ratio of catalyst to ligand (L) also played a decisive role in this conversion. As a Pd/L ratio of 1:4 gave a good yield whereas the ratio of 1:3 provided lower conversion. Variously substituted aryl tosylates and monoheteroaromatic tosylates gave the expected product in good yield. Only 3-cyano and 4-cyanophenyl tosylates gave the product under Pd-L1 catalytic system rather than Pd-SPhos. Also, the electronically neutral aryl mesylates gave a good yield of the products but the reaction was not feasible with sterically hindered mesylates. In 2015, group of M. Ghosh has designed a regioselective alkenylation of **375** with vinylarenes **189** through a CDC reaction [212]. The attractive feature of this report were the ligand-free conditions and use of molecular oxygen as green oxidant. The coupling was catalyzed by palladium acetate (Pd(OAc)_2_) in the presence of ammonium salt (TBAB) as an additive that was thought to stabilize the active palladium species formed during the reaction in the form of nanoparticles (Scheme 163). The reaction conditions developed were applicable enough for producing 18 compounds from differently substituted reactants in appreciable yield.

**Scheme 164:**
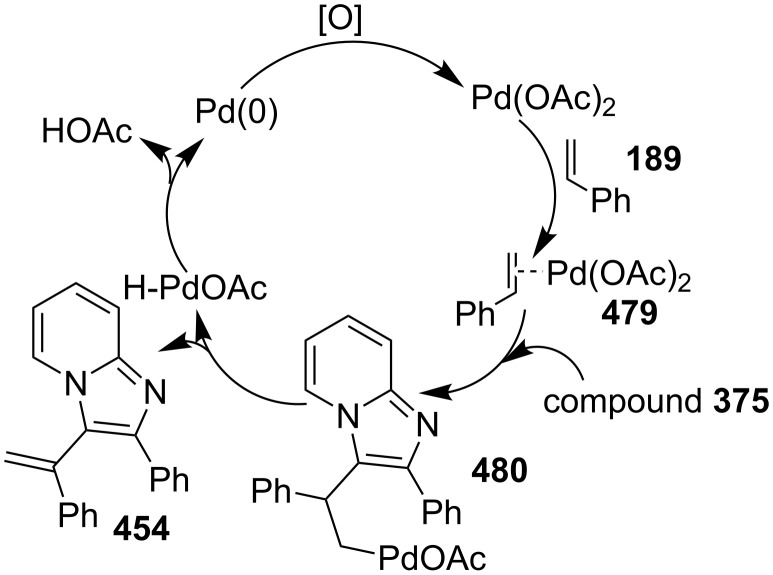
Plausible reaction mechanism for Pd(OAc)_2_-catalyzed synthesis of imidazo[1,2-*a*]pyridines.

Mechanistically, the reaction was thought to proceed via a styrene-Pd(OAc)_2_ intermediate. The intermediate was attacked by the nucleophilic C-3 position of the imidazopyridine, followed by β-hydride elimination that resulted in the final product **454**. Finally, palladium hydride (HPdOAc) formed in the reaction undergoes reductive elimination and oxidation by molecular oxygen to regenerate the Pd(II) catalyst (Scheme 164).

**Scheme 165:**
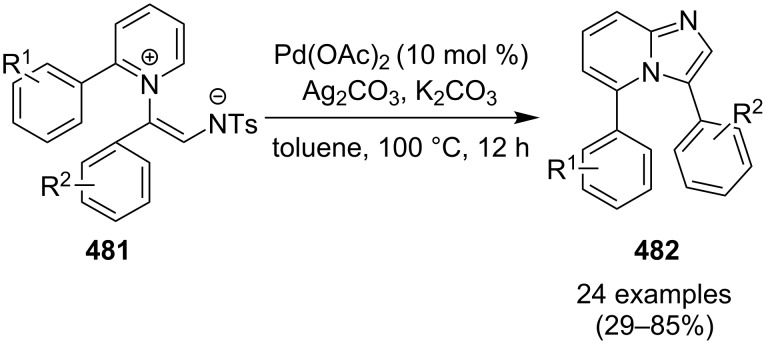
Pd-catalyzed C–H amination reaction.

The group of Lee and Shim have demonstrated a Pd-catalyzed C–H amination reaction for the synthesis of 3,5-diarylimidazo[1,2-*a*]pyridines **482** [213]. The synthesized compounds were further analyzed for their fluorescent properties by both experimental and theoretical studies. Thermal stability of the pyridinium zwitter ion **481** used as reactant led to the use of catalyst rather than depending on temperature and additives only for the formation of products by 1,5-electrocyclization. The reaction was successful with the application of Pd(OAc)_2_ as a catalyst, Ag_2_CO_3,_ and K_2_CO_3_ as oxidant and base respectively, at 100 °C (Scheme 165). Use of other inorganic bases was not found to be suitable for this transformation.

**Scheme 166:**
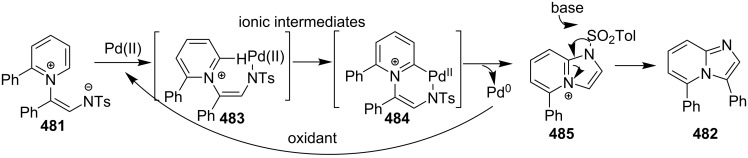
Mechanism for C–H amination reaction.

The reaction has a wider substituent tolerance with both EW as well as EDGs. The condition was applicable to several quinolinium zwitter ions producing the desired compound in 37–48% of yield. Mechanistically, Pd(II) form an ionic intermediate via C–H activation. The intermediate thus formed underwent reductive elimination and subsequent desulfonylation to give the final compound. The Pd(0) then reoxidized to form Pd(II) that participated in the next catalytic cycle (Scheme 166).

**Scheme 167:**
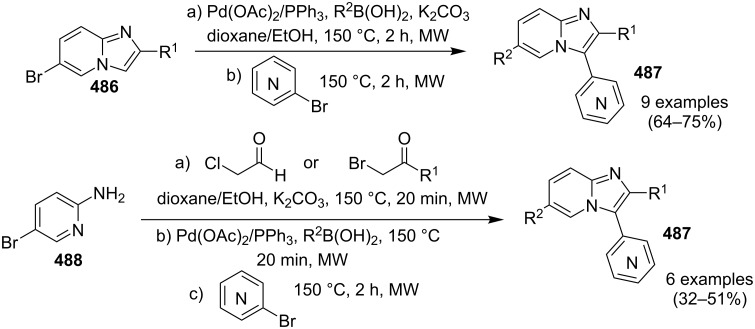
One-pot synthesis for 3,6-di- or 2,3,6-tri(hetero)arylimidazo[1,2-*a*]pyridines.

Koubachi et al. have discovered an interesting one-pot methodology for synthesizing 3,6-di- or 2,3,6-tri(hetero)arylimidazo[1,2-*a*]pyridines **487** [214]. The synthesis described two methodologies, first was a one-pot, two-step Suzuki coupling/heteroarylation of 2-substituted-6-bromoimidazo[1,2-*a*]pyridines **486** with arylboronic acid and heteroaryl bromide, second was a tandem cyclization/Suzuki cross-coupling/heteroarylation reaction starting with 2-amino-5-bromopyridine (**488**, Scheme 167).

**Scheme 168:**
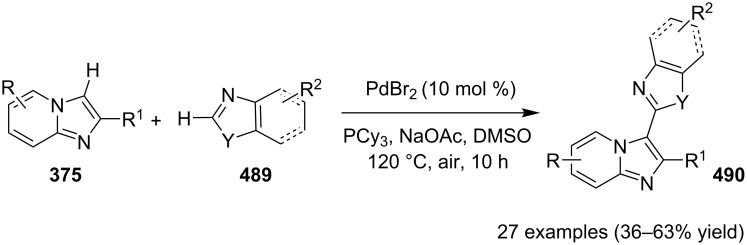
C–H/C–H cross-coupling reaction of IPs and azoles catalyzed by Pd(II).

**Scheme 169:**
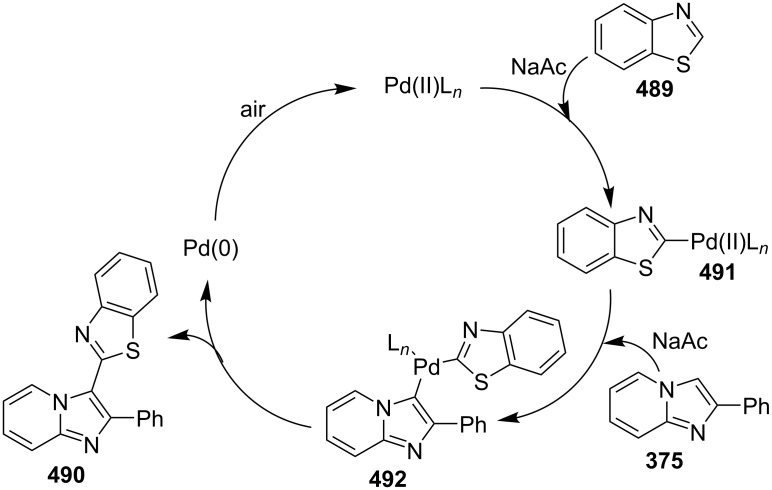
Mechanistic cycle.

This synthesis was carried out at 150 °C under microwave-assisted conditions. Pd(OAc)_2_/PPh_3_ was used as catalyst whereas the use of Pd(PPh)_3_ was not optimal. The Suzuki coupling and heteroarylation of the bromoimidazopyridine moiety led to product formation in good yield, however, initially when the reaction was performed with the 6-chloro derivative rather than the 6-bromo derivative of IP a poor yield was obtained. Another one-pot 3-step methodology involving a cyclization of 2-amino-5-bromopyridine with α-halogenocarbonyl derivatives followed by Suzuki coupling and heteroarylation reaction gave trisubstituted IP in moderate yields. Guo et al. have reported an efficient and effective C–H/C–H cross coupling of IPs **375** with azoles **489** catalyzed by palladium salt (Scheme 168) [215]. The protocol has offered a straightforward and simple methodology for the synthesis of 3-azolylimidazopyridines **490**. Inspired from a number of direct cross-coupling reactions of heteroarenes and versatile applications associated with IP and the azole core nucleus, the authors have carried out this direct cross-coupling reaction of IPs and azoles. Among different Pd and Cu salts tried for this conversion, PdBr_2_ was found to be optimal. Additionally, in order to increase the product yield, PCy_3_ and NaAc were used as ligand and base, respectively, under air atmosphere. The reaction was very versatile as it could be successfully carried out with a number of ED and EWGs on the phenyl ring at 2-position of the IP nucleus, also the presence of thienyl, naphthyl, and aliphatic chains gave a moderate yield of desired compounds. The presence of a methyl and a methoxy group on the pyridine nucleus also served well. Furthermore, substituted benzothiazoles and oxazoles have a good tolerance in this series without any steric hinderance. Mechanistically, the reaction was thought to be initiated by the coordination of Pd to the benzothiazole moiety aided by PCy_3_ ligand. The intermediate **491** thus formed reacted with **375** through a CMD pathway followed by reductive elimination to yield the final product and Pd(0). The Pd(0) was reoxidised by air and once again participated in the reaction cycle (Scheme 169). This protocol was step and atom economic thus opened up new pathways for similar exploration.

#### Rhodium-catalyzed derivatizations

**Scheme 170:**
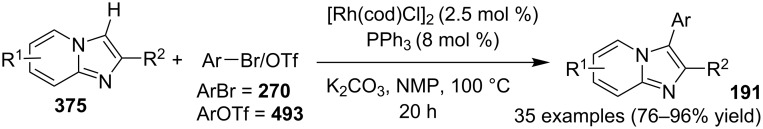
Rh-catalyzed C–H arylation reaction.

A C–H arylation reaction of **375** with aryl bromides or triflates **493** was reported by the group of Liu and He [216]. The synthesis was catalyzed by [Rh(cod)Cl]_2_ in the presence of K_2_CO_3_ as a base. Moreover, PPh_3_ was used as a ligand in order to increase the product yield (Scheme 170).

**Scheme 171:**
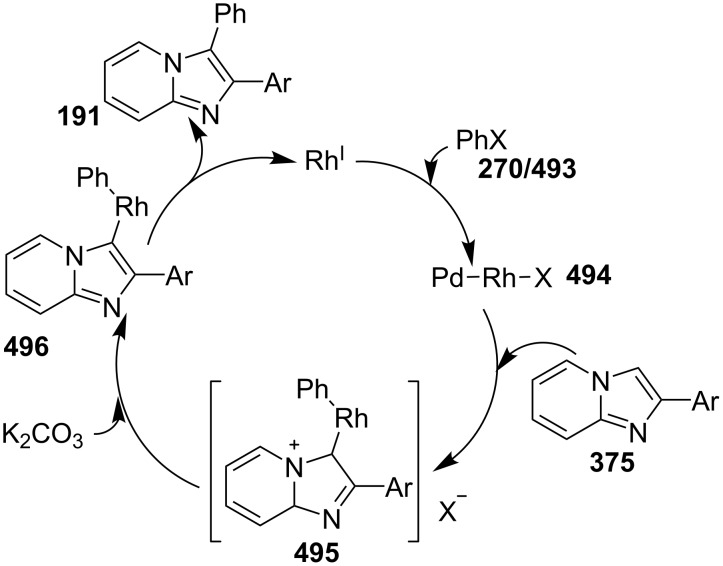
Mechanistic pathway for C–H arylation of imidazo[1,2-*a*]pyridine.

Imidazopyridines substituted at C-1, C-5 and C-6 positions found to be compatible under the reported reaction conditions generating a big library of arylated compounds. The reaction involved the formation of aryl-Rh halide species that was attached to IP **375** in an electrophilic manner. The intermediate **495** thus formed undergoes a hydrogen abstraction with the help of a base, followed by reductive elimination to give **191** with reproduction of the Rh catalyst (Scheme 171).

**Scheme 172:**
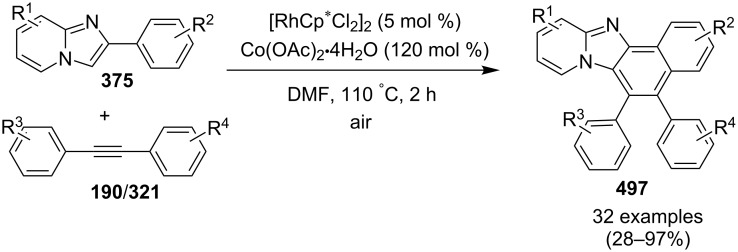
Rh(III)-catalyzed double C–H activation of 2-phenylimidazo[1,2-*a*]pyridines and alkynes.

The group of Wang and Niu have reported a Rh(III)-catalyzed and Co(II) co-catalyzed C–H bond activation reaction for synthesizing a series of naphtho[1’,2’:4,5]imidazo[1,2-*a*]pyridines **498** [217]. There were many reports which demonstrated the use of a Rh(III) catalyst for annulations between arenes and alkynes. Inspired by the pre-existing protocols a Rh(III) catalyst has been employed by this group for direct double C–H activation of 2-phenylimidazo[1,2-*a*]pyridines with alkynes (Scheme 172).

**Scheme 173:**
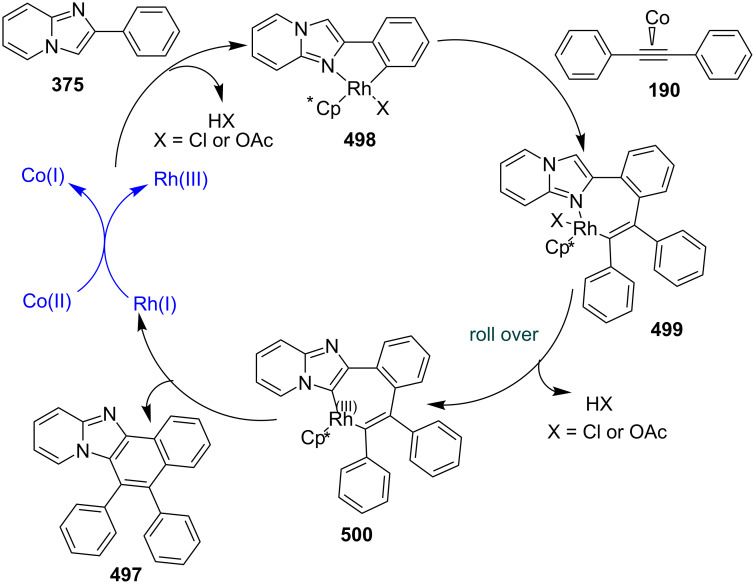
Rh(III)-catalyzed mechanistic pathway.

Optimization studies have clearly revealed that on decreasing the loading of the catalyst from 5 mol % and co-catalyst from 120 mol % produced a remarkable decrease in the product yield. Application of symmetrical alkynes with EWGs seemed to be more reactive than EDGs. On the other hand differently substituted unsymmetrical alkynes also well tolerated the reaction whereas alkyl-substituted alkynes resulted in a poor yield of the product. Furthermore, IPs substituted with EDGs found to be more reactive than EWGs. The reaction was thought to proceed by the formation of a 5-membered ring intermediate **498** with Rh(III) species involving the imidazo-nitrogen and the C-2 of the phenyl ring (C2-H has shown a remarkable D/H exchange of 96%). Further, insertion of a diarylalkyne resulted in the 7-membered rhodacycle intermediate **499**. This was followed by roll over C–H activation at C3 position and reductive elimination to afford the final compound. At the end of the reaction Co(Ac)_2_·4H_2_O reoxidized Rh(I) to Rh(III) as an active catalyst for further catalysis (Scheme 173). Application of other salts like copper(II) nitrate, copper(II) chloride, copper triflate, etc. did not result in better activity than cobalt acetate.

**Scheme 174:**
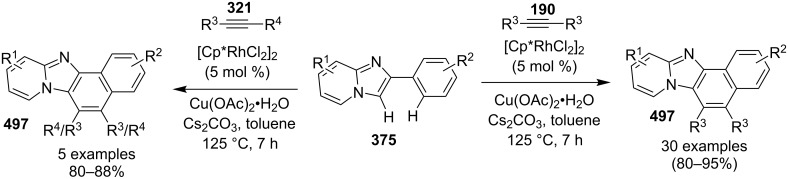
Rh(III)-mediated oxidative coupling reaction.

In the same year Kotla et al. have reported a Rh(III)-catalyzed oxidative coupling reaction of 2-arylimidazopyridines with internal alkynes **190** and **321** via double C–H activation [218] similar to that reported by Wang and Niu (Scheme 174) [217]. The difference between two reports was in terms of the oxidants and additives used, Cu(OAc)_2_·H_2_O was used as an oxidant and Cs_2_CO_3_ as an additive in this protocol, whereas Co(Ac)_2_·4H_2_O was used as oxidant without any additive in the former process.

**Scheme 175:**
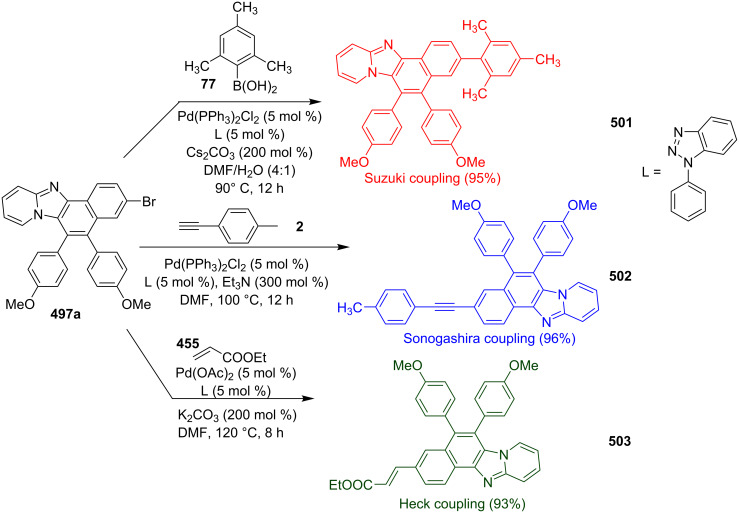
Reactions showing functionalization of the product obtained by the group of Kotla.

**Scheme 176:**
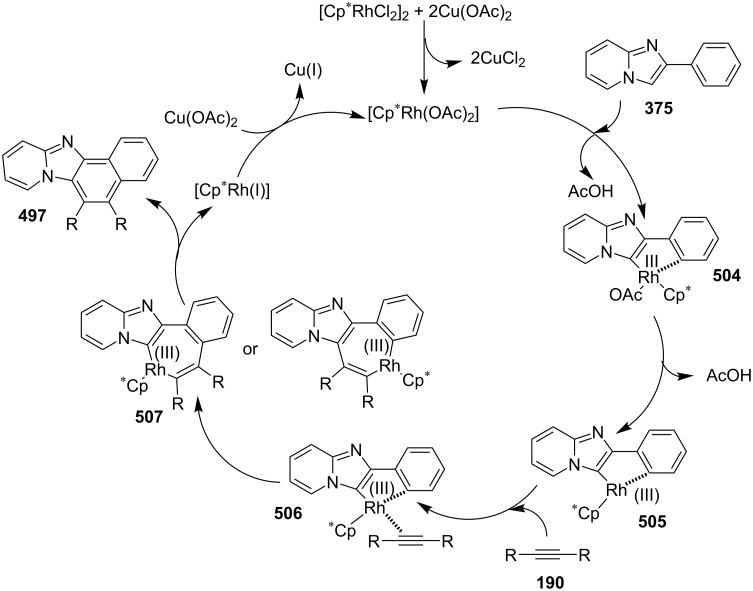
Mechanism for Rh(III)-catalyzed oxidative coupling reaction.

The reaction has a wider applicability as it has well tolerated both symmetrical as well as unsymmetrical alkynes; however, the reaction with unsymmetrical alkynes led to the formation of regioisomers. On the other hand, EDGs on both the pyridine ring and phenyl ring resulted in excellent yields of the desired products. The added advantage of the procedure was further functionalization of the bromo-substituted product by Heck (**503**), Suzuki (**501**) and Sonogashira (**502**) coupling reactions (Scheme 175). Mechanistically, the five-membered Rh(III) complex **241** was formed by double C–H activation of the IP nucleus. This complex then underwent π-complexation and alkyne insertion to form a seven-membered rhodacycle intermediate. This intermediate was assisted by cyclopentadiene ligand followed by reductive elimination to furnish the final product (Scheme 176). The synthesis has enjoyed a wide variety of functional groups on the reactants. Qi et al. have reported a selective mono versus 2-fold C–H activation catalyzed by Rh(III) under controlled conditions [219].

**Scheme 177:**
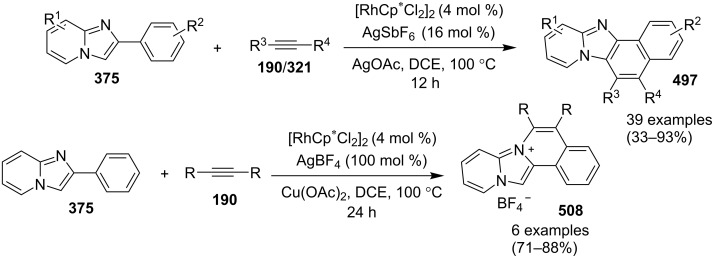
Rh(III)-catalyzed C–H activation reaction.

By using AgOAc as an oxidant naphthoimidazopyridines as the double C–H activation products were obtained, however, by replacing AgOAc with Cu(OAc)_2_ and using AgBF_4_ as co-oxidant the mono C–H activation product isoquinolinium salt **508** was obtained. The reaction was catalyzed by [RhCp*Cl_2_]_2_ and AgSbF_6_ as catalysts at 100 °C in DCE (dichloroethane) using silver salt as oxidant (Scheme 177). Control experiments have revealed that omission of AgSbF_6_ resulted in lower yields whereas by the removal of both the Rh complex and AgSbF_6_ the reaction became infeasible. Further examination of the reaction scope with 2-arylimidazo[1,2-*a*]pyridines and alkynes has revealed that the presence of both ED as well as EWGs at the *para*-position of the benzene ring was well tolerated with moderate to a good yield of the product. Also, –CH_3_, –OMe, halide and –CN groups on both the benzene and pyridine ring gave a good yield of the reaction products, however, with *meta* substitution two regioisomers were obtained. Moreover, symmetrical diarylacetylenes bearing different substituents resulted in an appreciable yield except for *m*-methyl and *o*-fluoro substitutions, though the coupling efficiency of unsymmetrical alkynes was high but a mixture of regioisomers was obtained that could not be separated chromatographically.

**Scheme 178:**
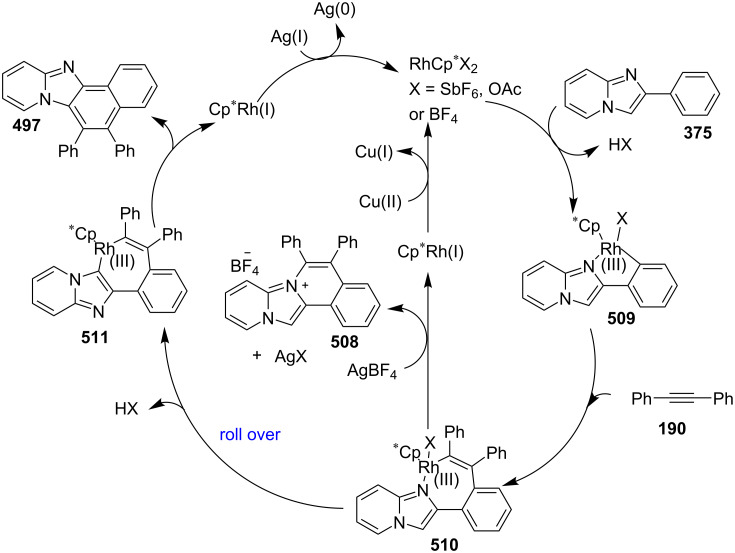
Mechanistic cycle.

**Scheme 179:**
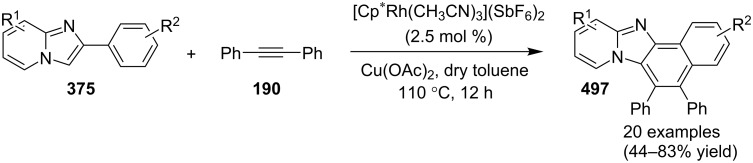
Annulation reactions of 2-arylimidazo[1,2-*a*]pyridines and alkynes.

Several experiments performed to explore the mechanism have indicated that in both single and double C–H activation initial N-coordination was followed by rollover C–H activation to yield double C–H activation product. Firstly IP was coordinated with Rh to form a rhodacycle intermediate which get converted to seven-membered intermediate **510** on insertion of alkyne. Nitrogen decoordination and rollover C–H activation resulted in the final intermediate that was followed by reductive elimination to yield the final compound and Rh(I) which was re-oxidized by AgOAc to the active catalytic species Rh(III). Alternatively, a reductive elimination of the C–N bond and anion exchange of the seven-membered intermediate resulted in a fused isoquinolinium salt. Reoxidation of Rh(I) to Rh(III) in this pathway was carried out by Cu(OAc)_2_ (Scheme 178). Furthermore, in a similar fashion an annulation reaction of 2-arylimidazo[1,2-*a*]pyridines with alkynes was described by Peng et al. for synthesizing benzimidazole derivatives [220]. The reaction was catalyzed by rhodium complex [Cp*Rh(CH_3_CN)_3_](SbF_6_)_2_ along with copper acetate as an oxidant in dry toluene (Scheme 179). The use of palladium acetate for this reaction was totally ineffective as it led to only a trace amount of the product.

**Scheme 180:**
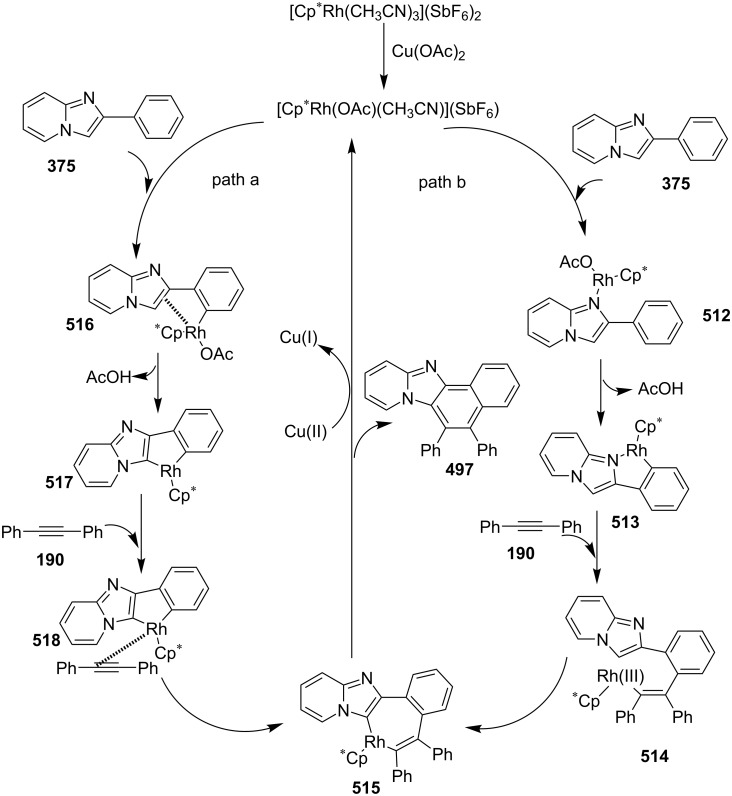
Two-way reaction mechanism for annulations reaction.

The synthesis has well tolerated different substituents on both the phenyl as well as pyridinyl ring in order to give excellent products. However, arylalkynes substituted with EWGs decreased the yield and use of aliphatic alkynes did not work under the reported conditions. In order to understand the reaction mechanism the kinetic isotopic effect (KIE) was studied which revealed the cleavage of the C–H bond in the phenyl ring to be the rate-limiting step. Two reaction pathways were reported by the authors both initiated by C–H bond cleavage (Scheme 180). At the end of the reaction a Rh(I) species was formed which was reoxidised by Cu(II) to Rh(III) to re-enter the catalytic cycle.

#### Ruthenium/iron/nickel/silver/vanadium/zinc-catalyzed derivatizations

**Scheme 181:**
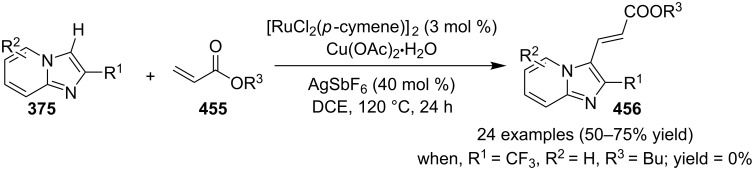
[RuCl_2_(*p*-cymene)]_2_-catalyzed C–C bond formation reaction.

Considering the tremendous interest of chemists in the Ru-catalyzed C–C bond formation reaction Zhan and Zhao et al. have proposed a highly regioselective oxidative olefination of **375** with **455** (Scheme 181) [221]. The catalytic activity of [RuCl_2_(*p*-cymene)]_2_ over Ru(acac)_2_ and RuCl_2_(PPh_3_)_2_ was remarkable in this olefination reaction. The use of copper(II) acetate and AgSbF_6_ as an oxidant and additive respectively has resulted in good to the excellent yield of the products in this transformation. The presence of the ruthenium catalyst was mandatory as its absence failed to promote this derivatization. Evaluation of the substrate scope has revealed that acrylates with both ED and EWGs were well tolerated in the reaction. However, in the case of IPs EDGs were well tolerated whereas groups like COOEt resulted in moderate yield and no product formation in case of CF_3_ as substituent. The regioselectivity of the reaction was explained by selective C-3 olefination even with 2,3-unsubstituted imidazopyridine.

**Scheme 182:**
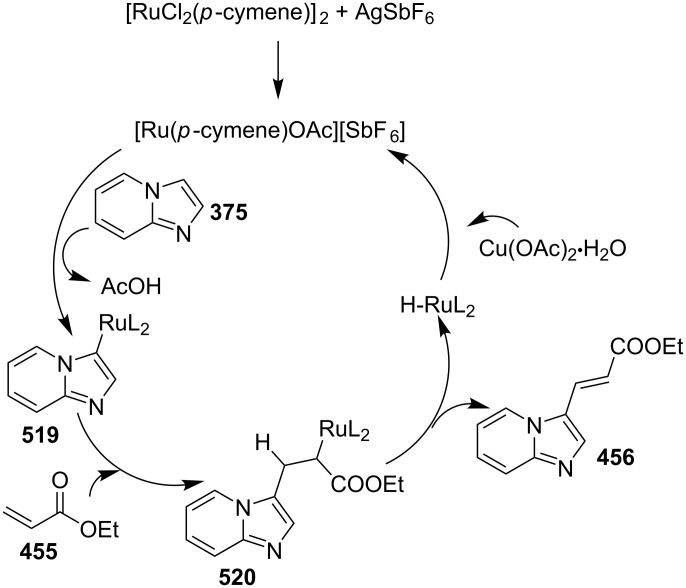
Reported reaction mechanism.

Mechanistically the reaction involved the migratory insertion of acrylate into the Ru–C bond of the intermediate formed between IP and the Ru complex. The so formed intermediate resulted in the final product formation by reductive elimination. Ru hydride produced at the end of this reaction was reoxidized by copper acetate to regenerate the active catalyst (Scheme 182).

**Scheme 183:**
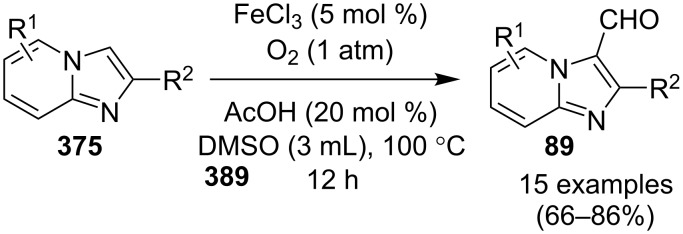
Fe(III) catalyzed C-3 formylation approach.

Ready availability, environmental compatibility, low price, and lower toxicity made iron-derived catalysts a chemist’s first choice. Although iron has been used in different forms to catalyze a number of organic reactions, reports on iron-catalyzed C–H functionalization are still scarce in the literature. Thus, from the scientific and environmental view point the group of Xiang and Chen have discovered a novel methodology for C-3 formylation of **375** catalyzed by iron(III) chloride (Scheme 183) [222]. The protocol did not require the use of any ligand and gave the product in moderate to good yield using oxygen as a greener oxidant. DMSO (**389**) used in this procedure acts as both the carbonyl carbon source as well as solvent. The optimization study of the reaction has revealed that the reaction was not viable in the presence of a base. Whereas the reaction proceeded under the acidic conditions especially in the presence of acetic acid, these results were similar to those reported by the group of Cao [127]. However, the absence of iron catalyst did not promote the reaction.

**Scheme 184:**
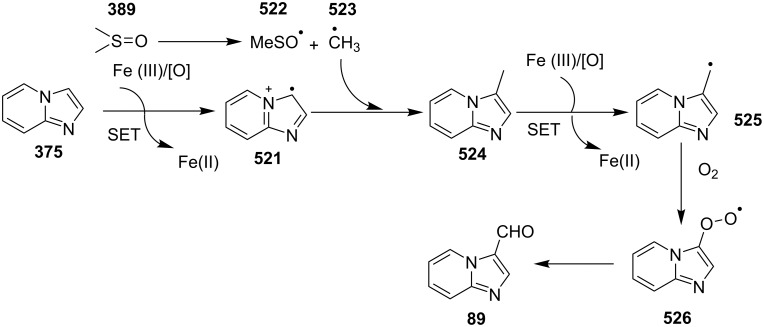
SET mechanism-catalyzed by Fe(III).

The reaction was suitable for a wide range of IPs substituted with both alkyl as well as phenyl groups. Moreover, IP substituted at C-2 position was found to give a slightly lower yield than the C-2-unsubstituted derivatives probably due to steric hinderance. The reaction was believed to involve a radical pathway as the use of radical inhibitors TEMPO and duroquinone inhibits the reaction. Mechanistically, the parent moiety **375** resulted in the formation of a radical intermediate **521** by SET oxidation assisted by ferric salt and oxygen. The intermediate thus formed was coupled with a methyl radical from DMSO to form methylated imidazo[1,2-*a*]pyridines **524**. SET oxidation of this methylated species followed by the formation of peroxy radical, resulted in final formylated product **89** (Scheme 184).

**Scheme 185:**
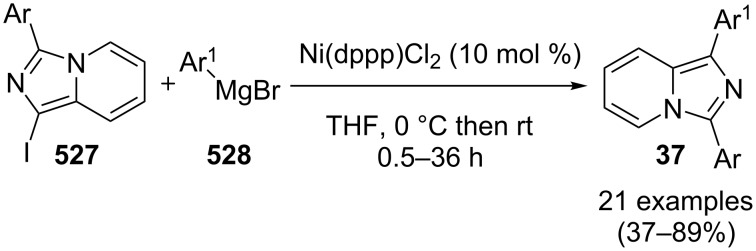
Ni(dpp)Cl_2_-catalyzed KTC coupling.

The group of Shibhara and Yamaguchi have reported Ni-catalyzed Kumada–Tamao–Corriu (KTC) and Pd-catalyzed Suzuki–Miyaura (SM) cross-coupling reactions for the arylation of 1-halo-3-arylimidazo[1,5-*a*]pyridines **527** [223]. In order to avoid the necessity of EWGs on electron-rich nitrogen-containing heteroaryl substrates for successful cross-coupling reaction, this protocol was investigated for efficient cross coupling even in the absence of EWGs. Halogenated imidazo[1,5-*a*]pyridines were synthesized by conventional electrophilic halogenations. Firstly, the KTC cross-coupling reaction was carried out between 1-iodo-substituted imidazo[1,5-*a*]pyridine and arylmagnesium bromide using NiCl_2_ at room temperature. However, this resulted in a satisfactory cross-coupled product (41%) along with a significant amount of homocoupled product from Grignard reagent (GR). Further optimization of nickel catalysts have shown that the use of Ni(dppp)Cl_2_ suppressed the homocoupled product and increased the cross-coupled yield up to 86% (Scheme 185). The reaction has well tolerated the substituted IPs and a variety of arylmagnesium bromides (GR). However, *p*-trifluoromethylimidazopyridine was proved to be a sluggish substrate with a slightly longer reaction time.

**Scheme 186:**
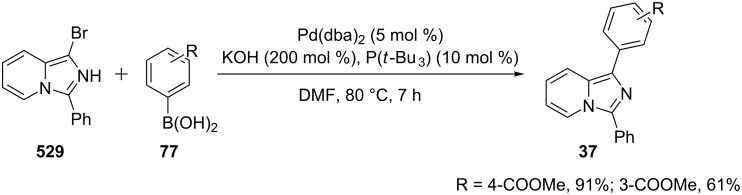
Pd-catalyzed SM coupling.

Further, in order to extend the substitution to ester, nitrile and nitro groups, a SM cross-coupling reaction was employed. Under the conditions of SM reaction, iodo-substituted IP **527** was not effective hence brominated IP **529** was used as a coupling partner with phenylboronic acids **77**. For the SM reaction Pd(dba)_2_ and Pd(*t*-Bu)_3_ were found to be an effective catalyst and KOH as a base to give a good yield of the cross-coupled product with *p*- or *m*-methoxycarbonylphenylboronic acids without the hydrolysis of the ester (Scheme 186).

**Scheme 187:**
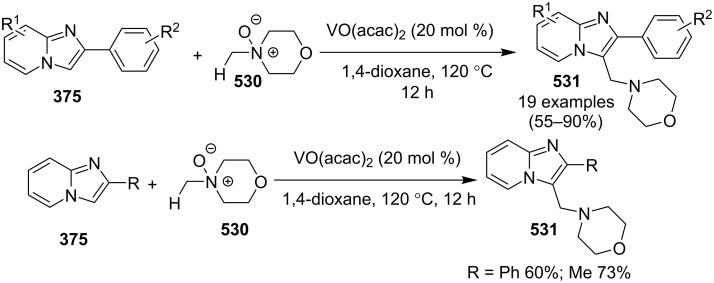
Vanadium-catalyzed coupling of IP and NMO.

The group of Kaswan and Porter have demonstrated vanadium-catalyzed orthogonal methodology as an alternative route to traditional Mannich type reaction for the introduction of tertiary amines into the heterocycles [224]. This methodology involved oxidative aminomethylation which has limited reports in the literature. The reaction involved a vanadium-catalyzed coupling of substituted 2-arylimidazo[1,2-*a*]pyridines with *N*-methylmorpholine oxide (NMO, **530**) at 3-position of IP in an efficient manner (Scheme 187). In this protocol, NMO served as both a coupling partner and as an oxidant. Initial use of vanadyl acetylacetonate (VO(acac)_2_) catalyst in the presence of DCM as solvent resulted in the formation of acetylacetonate as a byproduct that was difficult to remove from the product mixture. Thus, by changing the solvent to 1,4-dioxane and increasing the amount of catalyst up to 20 mol % the target compound was synthesized with high purity using 5 equiv of NMO.

**Scheme 188:**
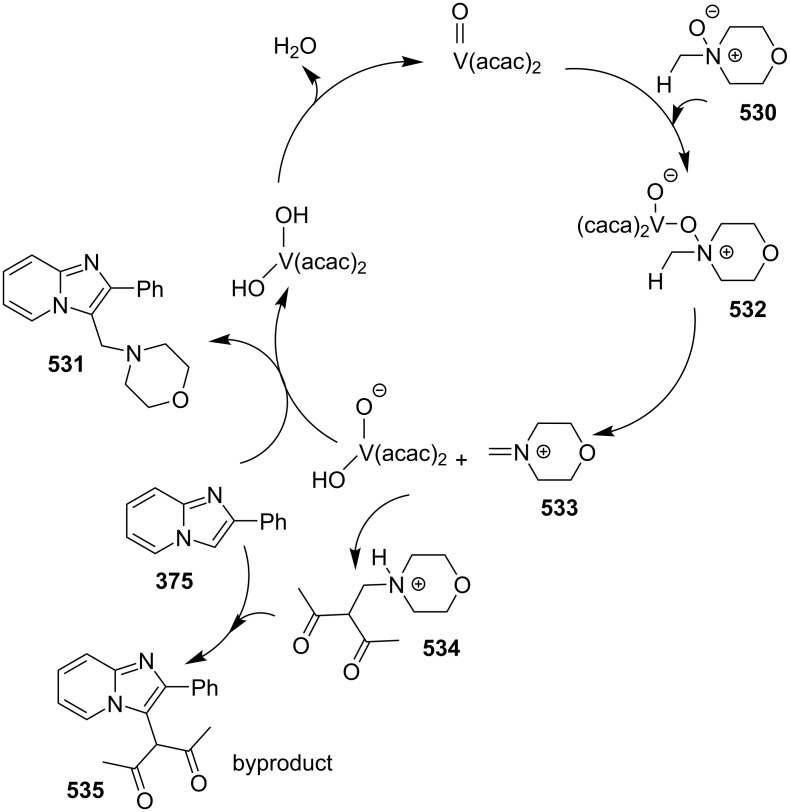
Mechanistic cycle.

The amount of NMO was also decisive as a decrease in its loading resulted in a decreased yield of the final product and increased the amount of unwanted byproduct. A study of substrates has revealed the favorable nature of EDGs over EWGs at the *para*-position of the phenyl ring in the imidazo[1,2-*a*]pyridine nucleus. No product was obtained with *para*-nitro substitution on the phenyl ring. The successful aminomethylation of 2-substituted indoles has shown the versatility of the protocol. The reaction was thought to proceed by Mannich-type mechanism via the ionic pathway, as the use of TEMPO could not affect the product yield. Mechanistically, the oxidation of VO(acac)_2_ by NMO resulted in the formation of an iminium ion. This was attacked by IP forming the final product with the elimination of a water molecule and regeneration of the catalyst. Also, a side product was thought to be formed from the reaction of iminium ion with one of the ligands from the catalyst (Scheme 188).

**Scheme 189:**
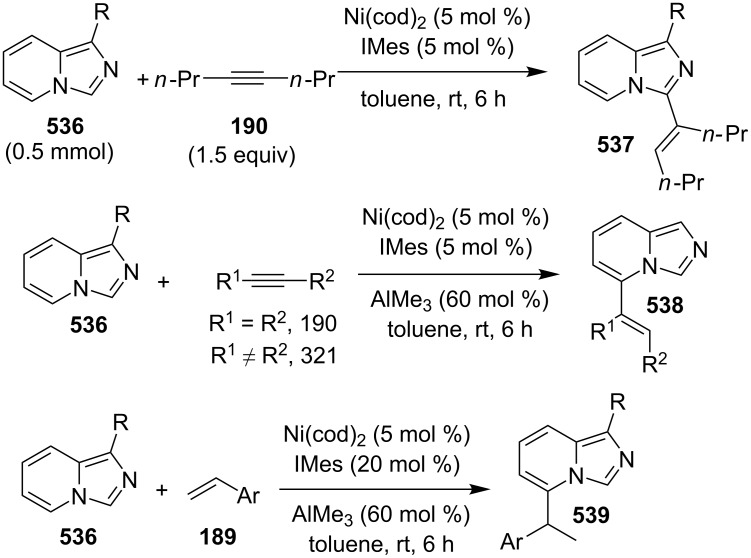
Selective C3/C5–H bond functionalizations by mono and bimetallic systems.

**Scheme 190:**
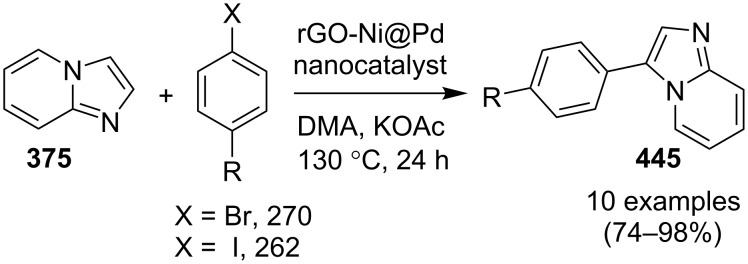
rGO-Ni@Pd-catalyzed C–H bond arylation of imidazo[1,2-*a*]pyridine.

The group of M.-S. Yu has reported a controlled C-3/C-5 functionalization of imidazo[1,5-*a*]pyridine with alkynes using the Ni/Al catalytic system [225]. The interesting part of their work was a selective C–H activation by wisely changing the mono metallic system to a bimetallic system. The use of Ni(cod)_2_ resulted in a C3–H bond activation whereas the use of a Ni-Al [Ni(cod)_2_ & AlMe_3_] bimetallic system resulted in C5–H functionalization of imidazo[1,5-*a*]pyridines (Scheme 189). The use of phosphine ligands was completely ineffective in this protocol whereas the use of IMeS and PCy_3_ gave the best yield. Encouraged by the group of Liu and co-workers [226] the use of AlMe_3_ along with the nickel complex resulted in selective C5–H activation and provided the products in good yields. Examination of the substrate scope has revealed different EW and EDGs to be well tolerated at the aryl ring at the C-1 site of the imidazo[1,5-*a*]pyridine nucleus for both C3/C5–H activation. This kind of C5-H activation was unexplored before this work. Further exploration of alkynes has revealed that symmetric (except diphenylacetylene), as well as asymmetric alkynes, were reactive enough to give a good yield with superior C5 selectivity. Moreover, the reaction has well tolerated the aliphatic olefins and styrenes with functional groups except for the sterically hindered *o*-CH_3_ and the strongly EW fluoro group. Successful C–H activation without using any directing group was an added advantage of this protocol. The group of Kilic and Turgut have developed an efficient direct C–H bond arylation reaction of imidazo[1,2-*a*]pyridines [227]. Aryl halides were used as an arylating agent and the reaction was catalyzed by monodispersed Ni@Pd core@shell NPs assembled on reduced graphene oxide (rGO) (rGO-Ni@Pd) as heterogeneous catalyst (Scheme 190).

This report unprecedently revealed the use of this catalyst for C–H bond activation. Although a large number of C–H activation reports were there but most of them relied upon homogeneous catalysis. Use of the bimetallic system gained attention due to the synergistic effect arising between the two distinct metals. rGO used as support enabled the close-range contact of the nanocatalysts and aromatic compounds of the reaction mixture via π–π interactions.

**Scheme 191:**
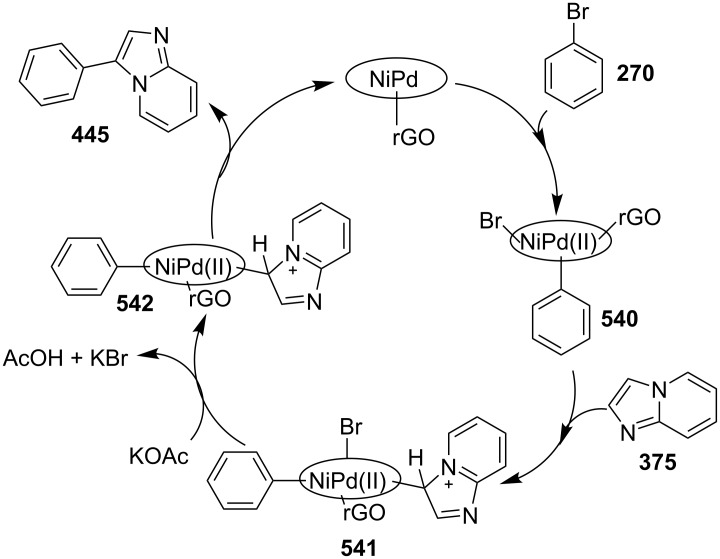
Mechanistic pathway for heterogeneously catalyzed arylation reaction.

The temperature in the reported reaction also proved to be a game changer, as lowering the temperature from 130 °C to 100 °C reduced the yield from 98% to only 10%. The use of the monometallic system of either Pd or Ni with rGO gave traces or 63% of the yield, respectively. This reaction was feasible with aryl iodides and bromides but not with chlorides. Halides with electron-rich as well as electron-deficient substituents and 3-bromopyridines gave the product in good yield. Mechanistically, aryl halide coordinated to the Pd shell of the catalyst by the oxidative addition to form an intermediate, which underwent electrophilic aromatic substitution to form the Wheland intermediate. This was followed by the loss of KBr, deprotonation and reductive elimination to give the final compound. The beauty of this protocol was the reusability of the catalyst up to 4 synthetic cylces (Scheme 191).

**Scheme 192:**
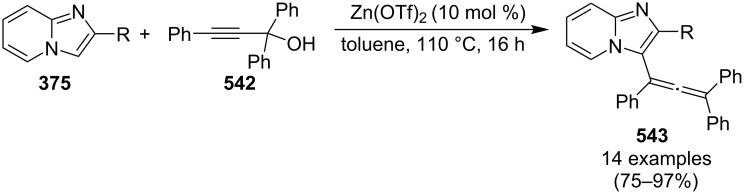
Zinc triflate-catalyzed coupling reaction of substituted propargyl alcohols.

In 2016 S. Jana et al. have reported a zinc triflate (Zn(OTf)_2_)-catalyzed coupling reaction of allenes at C-3 position of imidazo[1,2-*a*]pyridines (Scheme 192) [228]. This synthetic strategy was reported unprecedentedly using Zn(OTf)_2_, application of other Lewis acids including the triflates of silver, copper, and indium were also optimized but did not give a good yield of the product.

The reaction conditions developed were very versatile as it resulted in tetrasubstituted allenes with up to 97% yield. The reaction was feasible with imidazopyridines having aryl/heteroaryl moiety at C-2 position with different substitutions on both pyridine ring as well as on aromatic ring at the C-2 position. However, the presence of aliphatic groups on the imidazopyridine did not work well. The practical applicability of this method was in the successful production of desired compounds up to gram-scale level.

## Conclusion

A wider range of IP analogs has taken a lead role in the recent literature as pharmaceutical products and as reaction substrates in various organic transformations. Syntheses of several new derivatives of IPs have been reported considering either new synthetic routes or by performing modification in the pre-existing methodologies. In this review article, we have summarized an updated account of advances in the arena of TM-catalyzed syntheses of IPs. The reported reactions have been observed to explore the role of TMs as a catalyst in their various forms viz., salts, oxides, complexes or mixed oxides. The reactions described have demonstrated that incorporation of TMs both in heterogenous as well as homogenous forms, were leading to efficient and economic syntheses of IPs. Furthermore, many of these approaches have been applied successfully for the syntheses of commercial drugs like saripidem, zolimidine, and necopidem on gram scale in good yields. Conclusively it was inferred that the use of TMs not only increased the reaction yields but also avoided the formation of side products in many cases. We hope that the present review will create an impulse towards the development of new catalytic systems as heterogeneous, homogeneous and greener protocols generating feasible reaction methodologies for a number of variedly substituted IPs.
